# Mapping crown rust resistance at multiple time points in elite oat germplasm

**DOI:** 10.1002/tpg2.20007

**Published:** 2020-04-02

**Authors:** Ian G. McNish, Cristiano M. Zimmer, Alexander Q. Susko, D. Jo Heuschele, Tyler Tiede, Austin J. Case, Kevin P. Smith

**Affiliations:** ^1^ Dep. of Agronomy and Plant Genetics Univ. of Minnesota 1991 Upper Buford Circle, 411 Borlaug Hall St. Paul MN 55108; ^2^ Dep. of Crop Science Federal Univ. of Rio Grande do Sul Porto Alegre RS Brazil; ^3^ Pepsico Rhinelander WI 54501; ^4^ Anheuser‐Busch Fort Collins CO 80524

## Abstract

Crown rust, caused by *Puccinia coronata* f. sp. *avenae* Erikss., is the most important disease impacting cultivated oat (*Avena sativa* L.). Genetic resistance is the most desirable management strategy. The genetic architecture of crown rust resistance is not fully understood, and previous mapping investigations have mostly ignored temporal variation. A collection of elite oat lines sourced from oat breeding programs in the American Upper Midwest and Canada was genotyped using a high‐density genotyping‐by‐sequencing system and evaluated for crown rust disease severity at multiple time points throughout the growing season in three disease nursery environments. Genome‐wide association mapping was conducted for disease severity on each observation date of each trial, area under the disease progress curve for each trial, heading date for each trial, and area under the disease progress curve in a multi‐environment model. Crown rust resistance quantitative trait loci (QTL) were detected on linkage groups Mrg05, Mrg12, Mrg15, Mrg18, Mrg20, and Mrg33. None of these QTL were coincident with a days‐to‐heading QTL detected on Mrg02. Only the QTL detected on Mrg15 was detected in multiple mapping models. The QTL on Mrg05, Mrg12, Mrg18, Mrg20, and Mrg33 were detected on only a single observation date and were not detected on observations just days before and after. This result uncovers the importance of temporal variation in mapping experiments which is usually ignored. It is possible that high density temporal data could be used to more precisely characterize the nature of plant resistance in other systems.

**Abbreviations**
ACHAC: adjacency‐constrained hierarchical agglomerative clustering
AMOVA: analysis of molecular variance
AUDPC: area under the disease progress curve
CORE: Collaborative Oat Research Enterprise
GWA: genome‐wide association
LD: linkage disequilibrium
MAF: minor allele frequency
MAS: marker assisted selection
PCA: principal component analysis
POGI: Public Oat Genotyping Initiative
Q‐Q: quantile‐quantile
QTL: quantitative trait loci
SNP: single nucleotide polymorphism
UEOPN: Uniform Early Oat Performance Nursery
UMOPN: Uniform Midseason Oat Performance Nursery
UOPN: Uniform Oat Performance Nurseries

## 1 INTRODUCTION

Oat (*Avena sativa* L.) is a crop grown for grain, forage, and straw. Oat production in the United States has decreased from 19.1 million acres in 1973 (USDA Crop Reporting Board, 1975) to 2.5 million acres in 2017 (USDA Crop Reporting Board, 2017). This 89% decrease has strained the oat processing industry which has had some difficulty sourcing high quality milling oats from the United States in recent years. One strategy to increase the amount of oat production in the United States is to develop high quality oat cultivars that meet growers’ needs. Disease resistance is one of the most important characters that growers seek in oat cultivars making the improvement of disease resistance an important breeding objective. Crown rust, caused by the fungal pathogen *Puccinia coronata* f. sp. *avenae* Erikss., is the most important disease worldwide of cultivated oat (Nazareno et al., 2018). Losses due to crown rust are regularly 5% of yield in Minnesota and the American Upper Midwest, but losses have been observed to exceed 50% of yield in epidemic years (USDA‐ARS Cereal Disease Laboratory, 2016).

The crown rust disease cycle is complex and promotes a diverse *P. coronata* population. The disease cycle begins in the spring with a sexual stage on the alternate host buckthorn (*Rhamnus cathartica* L.) where novel combinations of pathogenicity genes are able to form (Simons, 1985). Buckthorn is widespread throughout the American Upper Midwest meaning locally adapted races from the previous season are able to successfully overwinter and infection is initiated early in the growing season. The sexual stage is followed by an asexual stage on *A. sativa* (Simons, 1985) where additional combinations of pathogenicity genes are able to form parasexually (Bartos, Fleischmann, Samborski, & Shipton, 1969; Chen et al., 2017). The asexual stage of the crown rust disease cycle is polycyclic (Simons, 1985), increasing the size of the *P. coronata* population many orders of magnitude and potentially resulting in an increased frequency of individuals with mutations to virulence (Groth & Roelfs, 1982).

Crown rust disease is managed by a combination of genetic resistance and foliar fungicide application. Crown rust dependably develops in the northern growing regions each year despite the use of fungicides (May et al., 2014). Genetic resistance is the more desirable management strategy because of the low cost to growers and because genetic resistance poses no risk to the environment. Two forms of genetic resistance to crown rust have been described in cultivated oat. The first form is race‐specific resistance in a gene‐for‐gene system (Jones & Dangl, 2006). Resistance in this system is qualitative and often complete. Unfortunately, race‐specific resistance is often not durable because it can be quickly defeated by a rapidly evolving pathogen population (McCallum, Fetch, & Chong, 2007). Many sources of race‐specific resistance have been described in the crown rust system (Carson, 2011; Chong, Gruenke, Dueck, Mayert, & Woods, 2008), but few of these sources have been mapped. Among the race‐specific resistance genes that have been mapped are *Pc38* (Wight, O'Donoughue, Chong, Tinker, & Molnar, 2004), *Pc39* (Wight et al., 2004), *Pc45*/*PcKM* (Kebede et al., 2019), *Pc48* (Wight et al., 2004), *Pc53* (Admassu‐Yimer, Bonman, & Klos, 2018), the *Pc58* gene complex (Hoffman, Chong, Jackson, & Obert, 2006), and *Pc91* (McCartney et al., 2011).

Another pattern of resistance, called quantitative resistance, is partial, not race‐specific, and is believed to be more durable than race‐specific resistance. No particular mode of action is attributed to quantitative resistance and it is likely that quantitative resistance is accomplished through a variety of mechanisms (Eloarce et al., 2016). Quantitative resistance is often correlated with plant maturity traits which should be considered in mapping investigations (Diaz‐Lago, Stuthman, & Abadie, 2002). Unlike resistance in the race‐specific system, quantitative resistance is influenced by many genes with small effects (Diaz‐Lago et al., 2002; Parlevliet, 1978). If genes with large effect size can be identified, then it is possible that quantitative crown rust resistance could be rapidly improved by marker assisted selection (MAS).

**Core Ideas**

- The University of Minnesota Oat Founder population is diverse and elite.
- Quantitative resistance to crown rust in elite oat germplasm is influenced by multiple genetic loci.
- Temporal variation impacts the detection of oat crown rust resistance loci within environments.
- Few oat crown rust resistance loci are detected at multiple time points within a season.

One notable source of durable quantitative resistance is the line MN841801 (Leonard, 2002). Portyanko et al. (2005) and Acevedo et al. (2010) identified eight QTL influencing quantitative resistance in MN841801. However, a single consistent and large effect size QTL was identified in three biparental crosses by Lin et al. (2014). Other sources of quantitative resistance have been identified by biparental mapping. Sunstrum et al. (2019) detected four QTL in a southern‐by‐northern oat population, Admassu‐Yimer, Gordon, Bonman, and Esvelt Klos (2019) described two QTL using a qPCR system to quantify the severity of crown rust resistance, and Babiker et al. (2015) described three QTL in another series of biparental crosses. Additional sources of quantitative and race‐specific resistance have been described by genome‐wide association (GWA) mapping in recent years. Esvelt Klos et al. (2017) characterized crown rust resistance in a collection of elite North American lines. Twenty‐two QTL were detected, but only three of the detected QTL were associated with quantitative resistance. Montilla‐Bascón et al. (2015) characterized crown rust resistance in a collection of European oat lines and identified five QTL influencing quantitative resistance. Finally, Winkler et al. (2016) conducted GWA mapping using a population of global landraces and historically cultivated lines which described six total QTL, but just one QTL associated with quantitative resistance.

Both Esvelt Klos et al. (2017) and Montilla‐Bascón et al. (2015) observed substantial genotype‐by‐environment interactions and the small number of QTL described so far are unlikely to explain the amount of genetic variance observed for this trait. These results suggest a single GWA or biparental mapping investigation is inadequate to characterize the genetic architecture of quantitative crown rust resistance and that there is value in evaluating additional populations in additional environments. Also, genetic mapping analyses of disease resistance traits are usually conducted using a single phenotypic observation made around the time of peak severity but before senescence has begun. Crown rust, however, is a polycyclic disease and disease severity varies throughout a growing season (Diaz‐Lago, Stuthman, & Leonard, 2003). It is possible that the genetic influence of crown rust resistance changes throughout the growing season as the host, environmental conditions, and the pathogen population change. Thus, analyses that incorporate measurements of disease severity at multiple time points may more fully describe the genetic architecture of crown rust resistance.

The University of Minnesota Oat Founder Population, hereafter referred to as the Founder population, is a collection of 256 elite northern oat lines sourced from eleven breeding programs in the American Upper Midwest and Canada. The original purpose of the Founder population was to serve as the base germplasm of a reinitiated milling oat breeding program at the University of Minnesota. The Founder population is currently being evaluated for a large number of important agronomic traits increasing its value as a genetic mapping resource.

The objectives of this investigation are to: (i) genetically and phenotypically characterize the Founder population, (ii) identify QTL associated with quantitative crown rust resistance, and (iii) explore the value of mapping crown rust resistance QTL at multiple time points during the growing season.

## 2 MATERIALS AND METHODS

### 2.1 Germplasm

The Founder population was assembled using data from the Uniform Oat Performance Nurseries (UOPN; formerly the Quaker Oat Performance Nurseries). Performance data for lines from the American Upper Midwest and Canada for the period 2006–2015 were downloaded from the T3/Oat database (https://triticeaetoolbox.org/oat/). Breeding programs submit lines for evaluation to this network of common nurseries throughout the oat growing region of North America. New lines are submitted each year. Selection was limited to lines from the Uniform Midseason Oat Performance Nursery (UMOPN) and Uniform Early Oat Performance Nursery (UEOPN). The traits of interest included grain yield, test weight, lodging severity, crown rust severity, beta‐glucan, barely yellow dwarf virus severity, and groat content. Line predictions were calculated using a mixed model with year, location, trial (e.g., UMOPN or UEOPN) within location, when applicable, and, when available, replication within trial fit as random effects. Of the 633 lines evaluated in the UOPN trials during this time period, 333 were identified as being ranked within the top fifty lines for at least one trait of interest and were requested from the corresponding breeders. Seed was ultimately received for 256 of these lines.

The Founder population was genotyped as part of the Public Oat Genotyping Initiative (POGI). In this initiative, oat breeding programs from the United States and Canada submitted lines in 2015, 2016, and 2017 for genotyping‐by‐sequencing (GBS). This collection of 1947 lines comprises the elite northern and southern oat germplasm of North America. Many of the lines genotyped through the POGI have also been evaluated in the UOPN. DNA preparation and sequencing were conducted in the lab of Dr. Shiaoman Chao at the USDA Agricultural Research Service laboratory in Fargo, ND as described in Saintenac, Jiang, Wang, and Akhu (2013). Bioinformatic analysis was conducted in the lab of Dr. Nick Tinker at the University of Ottawa using a custom SNP calling pipeline described in Tinker, Bekele, and Hatori (2016). This genotypic data was downloaded from the T3/Oat database without filtering for quality criteria. In the case of duplicate genotyping results, consensus genotypes were produced by accepting the most common non‐missing genotype at each locus for each line.

**Figure 1:**
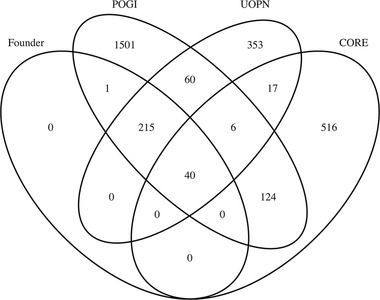
A Venn diagram describing the relationship of shared lines among the University of Minnesota Oat Founder Population (Founder), the Uniform Oat Performance Nursery Population (UOPN), the Public Oat Genotyping Initiative (POGI) population, and the Collaborative Oat Research Enterprise (CORE) population

Many of the lines in the Founder, UOPN, and POGI populations were also members of the CORE population (Tinker et al., 2016). The CORE was a multi‐institution project that ran from 2009 to 2013. The goals of this project were to genetically and phenotypically characterize elite oat germplasm, develop linkage maps, and develop high‐density genotyping technologies among others. The CORE population was evaluated for crown rust resistance (Esvelt Klos et al., 2017) and a small number of agronomic traits (Esvelt Klos et al., 2016). The relationship of shared lines among the Founder, UOPN, POGI, and CORE populations is described in Figure 1.

### 2.2 Population structure and linkage disequilibrium analyses

The *A.mat* function from the rrBLUP (Endelman, 2011) R (R Development Core Team, 2008) package was used to remove markers with >10% missing data and/or <2% minor allele frequency (MAF) and missing data points were imputed using the ‘mean’ method to reduce computation time. Principal component analysis (PCA) was conducted on the entire POGI population using the base R function *prcomp* and the first two principal components were visualized. K‐means clustering with k = 2 was then conducted which divided the population into northern and southern oat subpopulations based on the program of origin of each line. K‐means clustering with k = 3 was also conducted but failed to divide the population into more meaningful subdivisions. Analysis of molecular variance (AMOVA; Excoffier, Smouse, & Quattro, 1992) was conducted using the *pegas* (Paradis, 2010) R package to characterize the amount of genetic variance explained by the division of the POGI population into northern and southern subpopulations. The northern subpopulation was then isolated and the amount of genetic variance due to program of origin was characterized by AMOVA. Finally, the amount of genetic variance due to the division between Founder and non‐Founder lines in the northern oat subpopulation was characterized by AMOVA. Test statistic distributions for all AMOVA analyses were estimated by 200 permutations (nperm = 200).

The POGI genotypic data from the T3/Oat database was limited to lines in the Founder population and markers with map locations in the 2018 oat consensus map (Bekele, Wight, Chao, Howarth, & Tinker, 2018). The *A.mat* function from the rrBLUP R package was used to remove markers with >10% missing data and/or <5% MAF and missing data points were imputed using the ‘EM’ method. This genotypic data was used for all further analyses. PCA was conducted on the Founder population using the base R function *prcomp*. The first twenty‐five principal components were plotted in a scree plot and the first two principal components were visualized in a scatterplot. K‐means analysis was conducted to determine the number of clusters present in the PCA of the Founder population. Gap statistics (Tibshirani, Walther, & Hastie, 2001) were calculated for k = 1 to k = 10 using the *clusGap* function from the *cluster* (Maechler, Rousseeuw, Struyf, Hubert, & Hornik, 2019) R package with 100 bootstraps. The number of clusters used to describe the population was determined using the ‘1‐standard error rule’ (Tibshirani et al., 2001).

Linkage disequilibrium (LD) and linkage block analysis was conducted to identify groups of markers with common patterns of cosegregation for use in identifying groups of markers associated with crown rust resistance (Dehman, Ambroise, & Neuvial, 2015). Markers from each of the twenty‐one linkage groups in the 2018 oat consensus map were assumed to be independent from markers placed in other linkage groups. Squared allele‐frequency correlations (*r* ^2^) were calculated between all pairwise marker combinations for each linkage group. The *SNPclust* function from the *adjclust* R package was used to conduct adjacency‐constrained hierarchical agglomerative clustering (ACHAC) and linkage blocks were called within each linkage group by the *adjclust select* function using the ‘bstick’ method.

### 2.3 Field experiments

Crown rust disease severity was assessed at the University of Minnesota Matt Moore Memorial Buckthorn Nursery in St. Paul, MN. A full description of this nursery can be found in Al‐Kherb, Roelfs, and Groth (1987). Entries were planted in an augmented incomplete block design using primary and secondary checks in each incomplete block. Two crown rust trials were grown in 2017 and one crown rust trial was grown in 2018. Both crown rust trials in 2017 were planted in the same disease nursery. The 2017 early trial was planted on 5 May 2017 and the 2017 late trial was planted on 21 May 2017 to create two temporal environments. The 2018 trial was planted on 7 May 2018. Single row plots were 1.2 m long planted with 15 cm spacing and 3 g of seed. Trials were kept weed free by manual cultivation.

Crown rust infection is encouraged in this nursery by hedges of *R. cathartica* and spreader rows of a highly susceptible oat line. To ensure infection each season, a plot of a susceptible oat line is planted late in the growing season and allowed to become heavily infested by *P. coronata*. Straw from this plot is placed on the hedges of *R. cathartica* the following spring. As a result, heavy infection by a mixture of *P. coronata* races reliably develops each year. Disease severity was assessed on a whole plot basis by visually evaluating all available leaves on multiple plants using the modified Cobb scale (Peterson, Campbell, & Hannah, 1948) as a reference to produce a single disease severity score for each plot. All trials were rigorously observed during the early growth stages and disease severity observations began immediately at the first sign of crown rust infection (approximately Z14 in all trials; Zadoks, Chang, & Konzak, 1974) until crop maturity (Z94; Zadoks, Chang, & Konzak, 1974). Plots were scored two or three times each week. The scorer was blind to the previous scores, starting locations were randomized, and scoring direction was reversed each day so that each observation is independent. Area under the disease progress curve (AUDPC) was calculated by the trapezoid method using the *audpc* function from the *agricolae* R package (de Mendiburu, 2017). Relationships among the traits within each trial were explored using the *pairs.panels* R function.

### 2.4 Genome‐wide association mapping

Genome‐wide association mapping was conducted using the *GWAS* function from the *rrBLUP* R package for disease severity for each observation date for each trial, AUDPC in each trial, heading date for each of the 2017 trials, and for AUDPC in a multi‐environment model. Population structure was initially accounted for using the first three principal components fit as a fixed effect based on the cluster analysis result. However, the final models were run without accounting for population structure. Kinship was accounted for using a kinship matrix calculated using the *A.mat* function from the *rrBLUP* R package treated as a fixed effect. Trial was fit as a fixed effect in the multi‐environment model. Manhattan plots were produced for each model. Quantile‐quantile (Q‐Q) plots of the observed p‐values and the p‐values expected under the null hypothesis were visualized for each model to detect signs of test statistic inflation.

A modified Bonferroni threshold was calculated by determining the number of effective markers using the eigenvalues of a correlation matrix method (Li & Ji, 2005). In short, the genotypic data from each 2018 oat consensus map linkage group was analyzed separately to decrease the detection of spurious associations among markers placed on different linkage groups. A correlation matrix of the genotypic data was calculated for each linkage group and eigenvalue decomposition was conducted on each correlation matrix. The number of effective markers was then calculated as the number of eigenvalues greater than one plus the fractional value of each eigenvalue summed for all linkage groups. The modified Bonferroni threshold was then determined so that total a = 0.05

### 2.5 Post‐hoc QTL analysis

A mixed‐model was fit using the *mixed.solve* function from the *rrBLUP* R package. Trial and the marker avgbs_cluster_10965.1.20 were fit as fixed effects. Line was fit as a random effect with the covariance among the lines modeled using all other available markers by the *A.mat* rrBLUP function to account for the effect of background genetics.

## 3 RESULTS

### 3.1 Population structure

**Figure 2:**
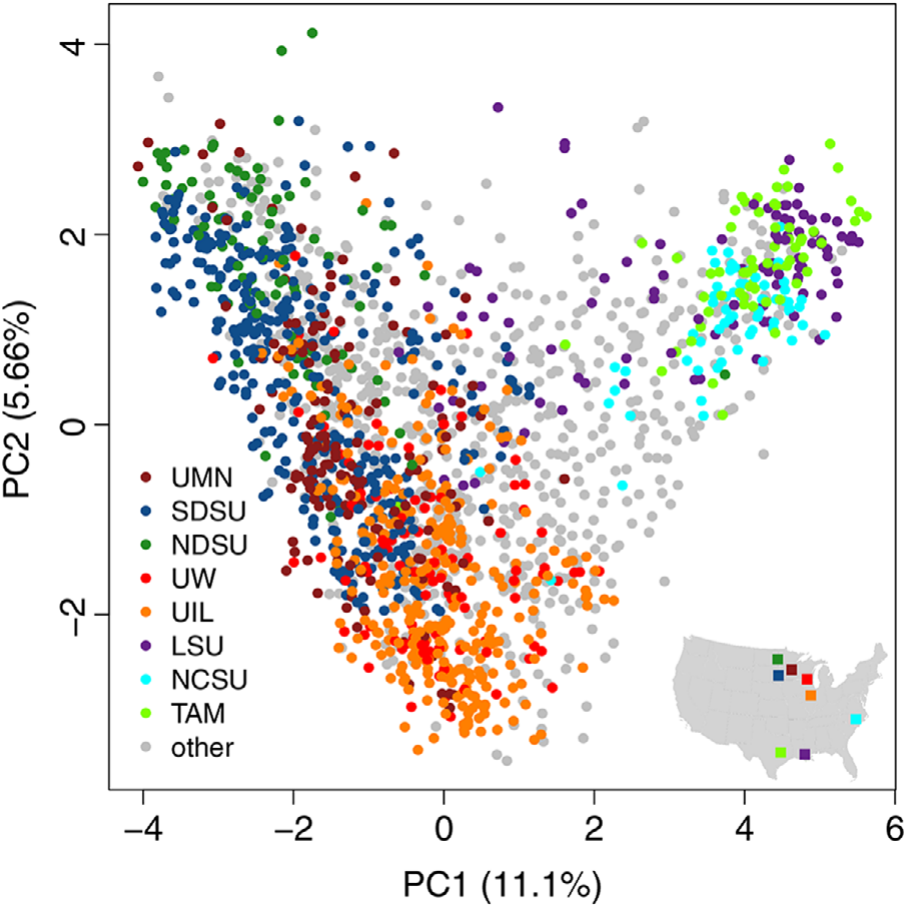
Population structure of elite North American oat lines genotyped by the Public Oat Genotyping Initiative as of December 2017 as revealed by principal component analysis. North American elite oat germplasm is divided into two groups. The northern oat group is composed of lines from the University of Minnesota–Twin Cities (UMN), South Dakota State University (SDSU), North Dakota State University (NDSU), the University of Wisconsin–Madison (UW), and the University of Illinois (UIL) programs while the southern oat group is composed of lines from the Louisiana State University (LSU), North Carolina State University (NCSU), and Texas A&M University (TAM) programs. The approximate location of each breeding program is shown in the lower right corner

**Figure 3:**
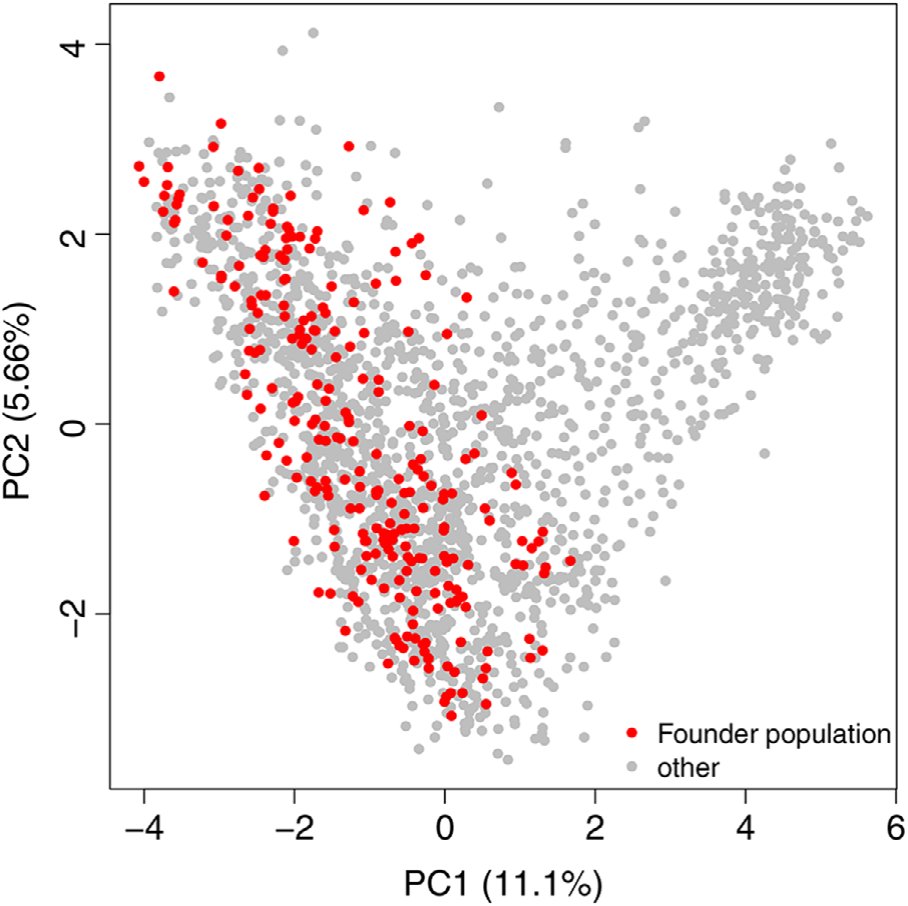
Population structure of the Founder population in relation to all Public Oat Genotyping Initiative lines as revealed by principal component analysis

The POGI population is currently the largest and most densely genotyped population of elite oat lines available; so, the population structure and genetic diversity of the POGI population and the relationship between the POGI and Founder populations was explored first. Genotypic data was downloaded from the T3/Oat database for 1946 POGI lines and 240,736 markers. A total of 32,256 markers and 1943 POGI lines remained after filtering for missing data and MAF. PCA of the POGI population reveals the North American elite oat germplasm is partitioned into two subpopulations (Figure 2). Division of the POGI population by k‐means analysis (k = 2) closely aligns with breeding program and region of origin. The northern subpopulation is composed of lines primarily from northern breeding programs such as the University of Minnesota‐ Twin Cities, North Dakota State University, South Dakota State University, and the University of Wisconsin‐ Madison while the southern subpopulation is mainly composed of lines from southern breeding programs such as Texas A&M University, North Carolina State University, and Louisiana State University. All Founder population lines are located within the northern oat subpopulation (Figure 3). AMOVA of the entire POGI population reveals that the division between the northern and southern oat subpopulations accounts for 20.27% (*p* < .001) of the genetic variance.

Some organization appears to exist within the northern oat subpopulation with the lines approximately arranged by breeding program moving from the most northern programs at one extreme of the cluster and the most southern programs at the other extreme of the cluster. AMOVA within the northern oat subpopulation reveals that the division among breeding programs accounts for 13.74% (*p* < .001) of the genetic variance. AMOVA also reveals the distinction between Founder and non‐Founder lines accounts for just 0.51% (*p* < .001) of the genetic variance within the northern oat subpopulation.

**Figure 4:**
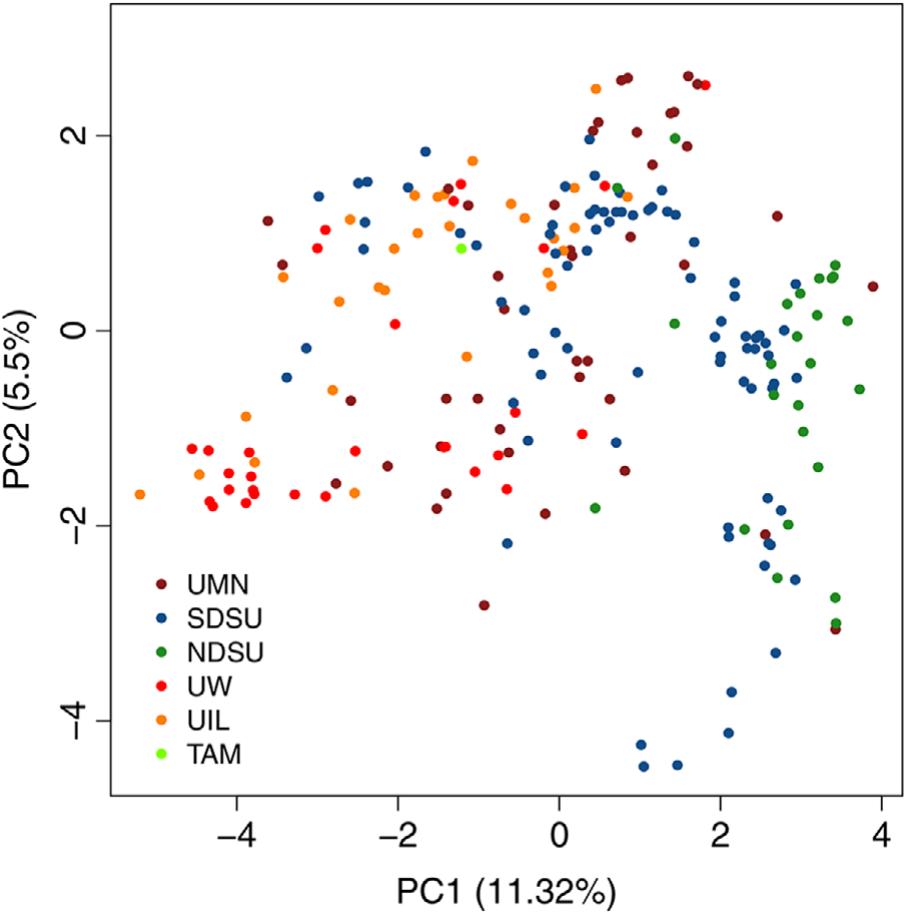
Principal component analysis of the University of Minnesota Oat Founder population

The PCA was also conducted on the Founder population alone to determine parameters for use in GWA mapping (Figure 4). The first three, five, and twenty‐five principal components of the Founder population account for 21.32, 28.87, and 62.52% of the genetic variation, respectively. A scree plot (Supplemental Figure S1) of the first twenty‐five principal components reveals a smooth decrease in the variance explained by the principal components with no obvious cutoff for the number of principal components to use to account for population structure during GWA mapping.

The plot of the gap statistic reveals that the gap statistic does not reach a maximum even when k becomes large (up to k = 25 not shown) (Supplemental Figure S2). The ‘1‐standard error’ rule suggests that the Founder population is best described by three clusters (Tibshirani et al., 2001).

The LD matrices for each 2018 oat consensus map linkage block are available in Supplemental Table S1. Linkage block assignments for each marker as determined by ACHAC are available in Supplemental Table S2. Heatmaps of pairwise *r* ^2^ and linkage block cluster analysis of each 2018 oat consensus map linkage group are available in Supplemental Figure S3. The 2018 oat consensus map measures 2964.6 cM (Bekele et al., 2018). The Founder population segregates at 13,519 high quality markers mapped to twenty‐one linkage groups on the 2018 oat consensus map producing 13,498 adjacent marker gaps. A total of 3347 of these gaps have an adjacent marker *r* ^2^ less than 0.1 covering 1614.69 cM or 54.46% of the 2018 oat consensus map length. This value is similar to association mapping populations for elite two‐row and six‐row barley that were found to have 61.9 and 51.7% of the genome represented by marker intervals with low *r* ^2^ (Falcon, Horsley, Hu, Blake, & Smith, 2018). The mean adjacent marker gap length is 0.20 cM and the maximum gap length is 28.20 cM and is found on Mrg28. The mean adjacent marker *r* ^2^ is 0.52.

### 3.2 Crown rust reaction

Disease severity early in the 2017 early trial was low, possibly due to a period of high heat and low precipitation in May that year. However, disease severity greatly increased after the conclusion of this dry period. All lines matured in mid to late July and were ready to harvest in August. Emergence of the 2017 early trial occurred before aecia were present on the adjacent rows of buckthorn; so, the primary inoculum of the 2017 early trial consisted largely of aeciospores. Growth in the 2017 late trial was slightly atypical. Lines headed relatively quickly after planting and reached maturity approximately 1 wk after the 2017 early trial. A small number of lines failed to head before the end of the season. Emergence of the 2017 late trial occurred after the nearby aecia stopped sporulating and uredinia (also called uredia) were present on the spreader rows; so, the primary inoculum in the 2017 late trial consisted mostly of urediniospores (also called uredospores or urediospores) originating from previously infected oat plants. Growth and development in the 2018 trial were normal in May, but hot and dry conditions accelerated the maturation of the crop in June. As a result, most lines reached physiological maturity in the first week of July which is approximately 2 wk earlier than normal. This decreased the length of the polycyclic phase of crown rust infection which resulted in lower than normal overall disease severity. Emergence in the 2018 trial occurred before aeciospores were present on the nearby buckthorn rows so the primary inoculum in the 2018 trial consisted primarily of aeciospores.

### 3.3 GWA mapping and genomic selection

Histograms of each trait within each trial and Pearson correlations (*r*) and scatter plots for each pairwise combination of traits within each trial are available in Supplemental Figure S4. Full GWA mapping results for all models are available in Supplemental Table S3. Manhattan and Q‐Q plots for all models are available in Supplemental Figure S5. The Q‐Q plots show that the relationship between the observed and expected p‐values is closely aligned with expectations under the null hypothesis without controlling for population structure in most of the models. Effective marker analysis detected a total of 2231 effective markers resulting in a modified Bonferroni threshold of *p* = 2.24 × 10^−5^ (−log_10_(*p*) = 4.65).

QTL associated with disease severity or AUDPC were detected on linkage groups Mrg05, Mrg12, Mrg15, Mrg20, and Mrg33 in at least one model (Table 1). The QTL detected on Mrg05, Mrg20, and Mrg33 are each associated with a single marker. The QTL detected on Mrg12 and Mrg18 are associated with multiple makers in perfect LD. Three markers were detected on Mrg15. Two of those markers are in perfect LD and placed in linkage block 2 and the third marker is placed in linkage block 6 by ACHAC.

**Table 1:** P‐values, as −log_10_(*p*), for markers at quantitative trait loci detected by genome‐wide association for crown rust disease severity and AUDPC in the University of Minnesota Oat Founder population. If multiple markers were detected in a QTL, then only one marker is displayed. Values where −log_10_(*p*) < 2 are omitted

Marker(s)	avgbs_120698.1.15	avgbs_cluster_15624.1.33	avgbs_cluster_10965.1.20	avgbs_63606.1.44	avgbs_cluster_33848.1.36	avgbs_51923.1.27	avgbs_200593.1.40
Linkage group^a^	Mrg05	Mrg12	Mrg15	Mrg15	Mrg18	Mrg20	Mrg33
Position (cM)^a^	10	95	3	24	37	63	48
Linkage block^b^	3	9	2	6	6	5	7
Resistant allele	C	C	T	A	A	A	C
Susceptible allele	T	G	G	G	G	G	T
Resistant allele frequency	0.91	0.35	0.06	0.41	0.21	0.06	0.07
2017 early AUDPC			3.25	2.27			
2017 early 7 June 2017							
2017 early 9 June 2017							
2017 early 12 June 2017	5.02^c^						
2017 early 14 June 2017						4.88^c^	
2017 early 16 June 2017							
2017 early 19 June 2017							
2017 early 21 June 2017			3.42				
2017 early 23 June 2017			3.01	4.71^c^	2.24		
2017 early 26 June 2017			3.74	2.12			
2017 early 30 June 2017			3.37				
2017 early 3 July 2017			2.76		2.34		
2017 early 5 July 2017			3.48		2.11		
2017 early 7 July 2017			2.13				
2017 early 10 July 2017							
2017 late AUDPC			5.16^c^		2.53		
2017 late 21 June 2017			2.82				
2017 late 23 June 2017			6.43^c^				
2017 late 26 June 2017			3.59				
2017 late 30 June 2017			3.79		2.08		
2017 late 3 July 2017			3.16		2.42		
2017 late 5 July 2017			3.20				
2017 late 7 July 2017			2.86				
2017 late 10 July 2017					5.01^c^		
2018 AUDPC		2.05	3.33				2.72
2018 11 June 2018			5.12^c^				
2018 13 June 2018							
2018 15 June 2018			2.40				2.20
2018 20 June 2018							
2018 22 June 2018		4.65^c^					5.01^c^
2018 25 June 2018			2.11	2.07			4.76^c^
2018 29 June 2018			2.68				
Multi‐environment AUDPC			4.82^c^	2.13			

a Linkage group and genetic positions are from Bekele et al. (2018).

b Linkage blocks are defined by adjacency‐constrained hierarchical agglomerative clustering.

c Markers significant at the modified Bonferroni threshold *p* < 2.24 × 10^−5^ (−log_10_(p) = 4.65) determined by effective marker analysis.

A single days‐to‐heading QTL associated with many markers at the 33 cM and 34 cM positions on Mrg02 was detected in both 2017 environments.

Mean AUDPC was 589.6 and the standard deviation was 342.2 in the multi‐environment model. *Post‐hoc* modeling of the Mrg15 linkage block 2 QTL estimates the effect size of the QTL is 69.8. So, this QTL is estimated to confer a 11.8% reduction in overall disease. The frequency of the resistant allele is 6.4% in the Founder population.

## 4 DISCUSSION

### 4.1 Founder population is elite, genetically diverse, and weakly structured

Analysis of the POGI population demonstrates that the Founder population is a representative sample of the genetic variation available within the elite northern oat subpopulation. The partitioning of genetic variance between the northern and southern oat subpopulations and among breeding programs within the northern subpopulation in the POGI population is concordant with observations of the CORE population (Esvelt Klos et al., 2016). The observation of low recombination on Mrg02 and Mrg28 in the POGI population is also concordant with observations of the CORE population and may be due to inversions in those linkage groups (Chaffin et al., 2016; Esvelt Klos et al., 2016). The representative nature of the Founder population makes it well suited to serve its primary objective as a source of germplasm for a milling oat breeding program at the University of Minnesota.

A population of elite oat lines representing both regional subpopulations and populations that included globally sourced landraces would likely have greater genetic diversity than a population of elite lines from the northern subpopulation alone. However, these more diverse populations would also have much more population structure and may be less desirable for use in GWA mapping. For example, the first five principal components of the Founder and oat landrace (Winkler et al., 2016) GWA populations account for 28.9% and 43.3% of the genetic variance, respectively. While the inclusion of a population structure component during GWA mapping is necessary to avoid spurious association, it also acts to erode signal from true positive associations that are correlated with population structure (Zhao et al., 2007). As a result, an ideal GWA mapping population would have very little population structure (e.g., Massman et al., 2011). Finally, researchers often develop relatively large mapping populations to increase power to detect QTL. Despite the relatively small population size (*n* = 256), the Founder population captures most of the genetic variation available in the elite northern oat subpopulation and therefore can be evaluated relatively inexpensively compared to larger mapping populations.

### 4.2 Several QTL are important for quantitative crown rust resistance

**Figure 5:**
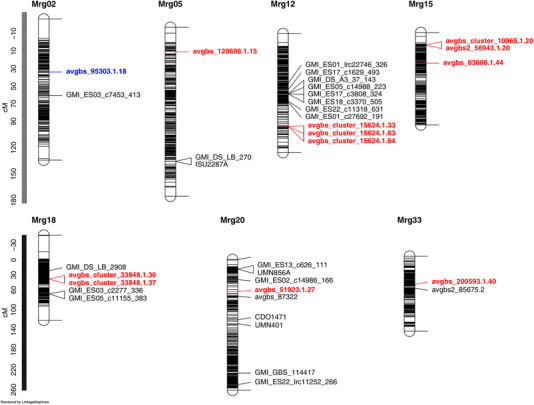
Ideograms of Mrg02, Mrg05, Mrg12, Mrg15, Mrg18, Mrg20, and Mrg33. The genetic length of each linkage group in centimorgans (cM) is indicated by the scales on the left. The location of markers on the 2018 oat consensus map (Bekele et al., 2018) are represented by the black horizontal lines on each linkage group. Markers associated with crown rust disease severity detected in this investigation are labeled in red. Markers associated with days‐to‐heading detected in this investigation are labeled in blue. Markers detected in other investigations are labeled in black

One crown rust QTL was consistently detected on Mrg15. Crown rust QTL were also detected on Mrg05, Mrg12, Mrg15, Mrg18, Mrg20, and Mrg33 in at least one model (Table 1; Figure 5). Most importantly, these five QTL were detected only on single observation dates with little or no signal of an association on observation dates just days before and after.

Two distinct QTL were potentially detected on Mrg15 (Table 1; Figure 5). No other investigation has mapped a crown rust resistance QTL to Mrg15 to our knowledge. One QTL on Mrg15 was associated with the markers avgbs_cluster_10965.1.20 and avgbs2_56943.1.20 located at the 3 cM position (Table 1). These markers were statistically significant in the 2017 late trial of 23 June 2017, 2017 late trial AUDPC, 2018 trial of 11 June 2018, and multi‐environment AUDPC models (Table 1). Additionally, these markers have statistically suggestive, but not statistically significant, p‐values in many of the other models evaluated. This QTL is not coincident with the days‐to‐heading QTL detected in this investigation or in other investigations. Finally, the effect size of this QTL was estimated to be relatively large by *post‐hoc* modeling and the resistant allele is relatively rare in the Founder population. These characteristics make this QTL a promising target for further investigation and breeding by MAS. A possible second QTL was identified on Mrg15 associated with the maker avgbs_63606.1.44 at the 23 cM position in the 2017 early trial 23 June 2017 model (Table 1). It is likely that this QTL is distinct from the QTL detected at the 3 cM position because the markers associated with each QTL are placed in different linkage blocks by ACHAC and because the QTL at the 23 cM position is detected just once while the QTL at the 3 cM position is detected frequently.

The crown rust QTL on Mrg05 was associated with the marker avgbs_120698.1.15 at the 10 cM position (Table 1; Figure 5). Several other studies have detected crown rust resistance loci on this linkage group. The CORE project QTL QPc.CORE.05 (Esvelt Klos et al., 2017) is associated with marker GMI_DS_LB_270 placed at the 135.5 cM position of Mrg05. Wight et al. (2004) identified an association between *Pc39* and ISU2287 (ISU2287A) which also located at 135.5 cM position indicating these two QTL are placed on opposite ends of Mrg05. Sunstrum et al. (2019) detected a QTL associated with avgbs_cluster_3439.1 located between the 57 cM and 89 cM positions. The lack of prior crown rust related findings near the 10 cM position on Mrg05 suggest this may be the first report of this QTL.

The crown rust QTL detected on Mrg 12 was associated with the marker avgbs_cluster_15624.1.33 at the 95 cM position (Table 1; Figure 5). Esvelt Klos et al. (2017) reported the QTL ‘QPc.CORE.12’ associated with the marker GMI_ES18_c3370_505 at the 58 cM position and Babiker et al. (2015) reported the QTL ‘QCr.cdl11‐13A’ and ‘QCr.cdl9‐13A’ associated with several markers in the 39–68 cM region. It is possible that this is a novel QTL given the genetic distance between the QTL detected in this investigation and the previously reported QTL.

The crown rust QTL on Mrg18 was associated with the markers avgbs_cluster_33848.1.36 and avgbs_cluster_33848.1.37 at the 37 cM position of the 2018 oat consensus map (Table 1; Figure 5). Esvelt Klos et al. (2017) previously reported QTL on Mrg18 associated with the marker GMI_DS_LB_2908 at the 21 cM position and the markers GMI_ES03_c2277_336 and GMI_ES05_c11155_383 at the 68 cM position of the 2018 oat consensus map. The QTL detected in this investigation may be a repeat detection of these QTL as the genetic position of the QTL is placed among the genetic positions of the previously reported QTL.

The crown rust QTL detected on Mrg20 was associated with the marker avgbs_51923.1.27 which is placed at the 63 cM position on the 2018 oat consensus map (Table 1). Several QTL associated with crown rust and days‐to‐heading have been previously reported on Mrg20. Two days‐to‐heading QTL, a trait commonly associated with quantitative crown rust resistance, were detected in Esvelt Klos et al. (2016). The nearest of these QTL was associated with avgbs_87322 at the 72 cM position. Three QTL associated with crown rust resistance were detected on Mrg20 by Esvelt Klos et al. (2017). The closest of these QTL is ‘QPc.CORE.20.1’ associated with the marker GMI_ES02_c14986_166 at the 84.20 cM position. Portyanko et al. (2005) identified a QTL, ‘Prq3’, associated with quantitative resistance and marker UMN856 (UMN856A) at the 18 cM position. It is possible that the QTL detected in this investigation is a repeat detection of one of these QTL given the close genetic proximity. Additional crown rust QTL have been detected at more distant positions including ‘QCr.cdl9‐19A’ associated with the marker GMI_DS_cc11093_89 at the 196.3 cM position (Babiker et al., 2015), GMI_ES22_lrc11252_266 at the 249.6 cM position (Admassu‐Yimer et al., 2019), a race‐specific resistance gene introgressed from *Avena strigosa* associated with the marker GMI_ES13_c626_111 at the 18 cM position (Rines et al., 2018), and *Pc48* associated with the markers CDO1471 and UMN401 at the 119.69 cM and 135.80 cM positions, respectively (Wight et al., 2004). It is unlikely that the QTL detected in this investigation is a repeat detection of one of these QTL given their genetic distance from the QTL and their race‐specific nature.

The crown rust QTL on Mrg33 was associated with marker avgbs_200593.1.40 which is placed at the 48 cM position of the 2018 oat consensus map (Table 1). Sunstrum et al. (2019) detected a nearby QTL associated with avgbs2_85675.2 at the 56 cM position. Montilla‐Bascón et al. (2015) detected an association between crown rust severity and the marker AME176‐3. This marker is reported to map to the chromosome 15A which is associated with the merged linkage group Mrg33 (Chaffin et al., 2016). Unfortunately, AME176–3 is not placed on the 2018 oat consensus map. The temporal detection of this QTL was slightly different than the other QTL detected in this study described above. The statistically significant observation date was surrounded by three statistically suggestive observation dates and was statistically suggestive in the 2018 AUDPC model. This indicates that the QTL on Mrg33 may be associated with a gene that provides a longer‐term impact on disease severity than the QTL on linkage groups Mrg05 and Mrg20.

Days to heading was also mapped because of the common association between maturity and disease severity (Diaz‐Lago et al., 2002). The marker avgbs_95303.1.18 at the 33 cM position of Mrg02 was found to be highly significant in both 2017 environments. This QTL has been previously reported by Winkler et al. (2016) associated with the marker GMI_ES03_c7453_413. This heading date QTL is not coincident with the crown rust QTL detected in this investigation. Visual analysis of the heading date Manhattan plots (Supplemental Figure S5) does not reveal any statistically suggestive peaks near the previously described crown rust QTL.

### 4.3 Quantitative resistance is difficult to distinguish from race‐specific resistance

Resistance to crown rust exists in distinct race‐specific and non‐race‐specific forms. Methods have been developed to detect and characterize each form. Race‐specific resistance is usually evaluated in a controlled greenhouse environment using seedlings and single‐spore crown rust isolates with a known avirulence pattern. Characterization of race‐specific resistance is a process of exclusion using this method (Wamishe, Thompson, & Milus, 2004). Observations are made using a categorical scale that describes the nature and degree of the observed hypersensitive response. Non‐race‐specific resistance is usually characterized in a field or disease nursery environment using natural or bulk captured inoculum composed of many races of the pathogen. Observations are usually made on adult plants using a quantitative scale that measures the proportion of leaf surface covered in disease pustules (Diaz‐Lago et al., 2002).

The lines in this investigation were evaluated in a field disease nursery, received diverse natural inoculum, and were evaluated using a quantitative scale to identify QTL associated with quantitative crown rust resistance. However, using these methods does not guarantee that the loci detected are not race‐specific. It is possible for differences in race‐specific resistance to produce quantitative differences in disease severity and the absence of race‐specificity is difficult to prove.

The USDA‐ARS Cereal Disease Laboratory conducts an annual survey of the *P. coronata* population in the American Upper Midwest. The most recent publication on this data revealed that the Midwestern *P. coronata* population was virulent to an average of 15.53 of thirty single resistance differential lines and that the average number of virulences per *P. coronata* isolate increased at a rate of 0.66 virulences per year in the northern oat region between the years 2001 and 2009 (Carson, 2011). Many of the resistance genes used in this survey are defeated with rates of virulence >90%. Based on the results of the 2016 USDA‐ARS CDL crown rust survey the resistance genes *Pc62*, *Pc94*, and *Pc96* with virulence frequencies of 19.3, 26.5, and 27.7% in Minnesota, respectively (unpublished data, USDA‐ARS CDL), are candidates for the Mrg15 linkage block 2 QTL because the corresponding avirulence genes have low frequencies of virulence in the pathogen population and the resistance genes are currently unmapped. However, the oat crown rust differential set is not exhaustive for all known crown rust resistance genes and many resistance genes are likely uncharacterized. The markers identified in this investigation could be used to backcross this QTL into a highly avirulent background. This QTL could be then further characterized by single‐isolate tests which may uncover patterns of race‐specificity.

### 4.4 Temporal variation of disease severity impacts trait mapping

Plant trait mapping experiments usually consider the effects of genotype and the environment by evaluating a mapping population in multiple environments. Some mapping experiments address the interaction of genotype and the environment by replicating the mapping population within each environment. Evaluation of the trait of interest is usually made at a single time point. These experimental designs are useful for traits such as grain yield or the deoxynivalenol toxin concentration in barley and wheat grain because these end point values are directly important for human use. Crown rust disease severity, however, is an important trait because of the impact crown rust disease has on other traits such as grain yield and grain quality (May et al., 2014; Simons, Youngs, Booth, & Forsberg, 1979). If disease severity can rapidly change and if there is substantial variation in the rate of change among the lines in the mapping population, then an end point measurement of disease severity may not capture the overall magnitude of disease pressure throughout the growing season. Thus, a single measurement may not be indicative of the impact the disease had on the grain yield and quality.

Crown rust disease severity is the result of a complex interaction between a plant, a pathogen, and the environment. *Puccinia coronata* is a dynamic organism and the life cycle stage, size, and genetic composition of the *P. coronata* population changes throughout the growing season (Al‐Kherb et al., 1987). *Puccinia coronata* encounters a host that progresses through the seedling, vegetative, and reproductive life stages. Finally, both host and pathogen encounter changing environmental conditions that sometimes favor the host and other times favor the pathogen (Kochman & Brown, 1976a, 1976b). Disease severity at any one time point is then influenced by the host, the pathogen, the environment, time, and the interactions among these factors which produce substantial temporal variation that most disease trait mapping experiments ignore. This investigation uncovers the importance of this temporal variation in trait mapping which should be carefully considered when evaluating the results of any other trait mapping experiment where the trait is similarly influenced by temporal variation.

The crown rust QTL detected on linkage groups Mrg05 and Mrg20 are notable because they are detected in only a single environment and on a single observation date. These QTL were both detected early in the season in the 2017 early trial. No signal that could be considered statistically significant or suggestive is observed just days directly before or after the significant observation date for either QTL. One possible explanation for the single‐date detection of the QTL on Mrg05 and Mrg20 is that these QTL may be associated with resistance genes that influence the latency period. Previous investigations have detected differences of crown rust latency period between resistant and susceptible cultivars of 2 d (Luke, Barnett, & Pfahler, 1984) and 2.5 d (Diaz‐Lago et al., 2003) which could explain the change in signal within just a couple days. These observations were also made during the oat vegetative growth stage. If periods of rapid growth coincide with periods when no free water is available on the plant leaves for new infections to initiate, then it is possible for whole plant severity to decrease as occurred in the 2017 early trial of this investigation. Thus, a second possible explanation of these observations is that they are associated with genes that regulate growth in young plants. As these observation dates were just five and 7 d after the first signs of disease were observed, it is likely that the disease pustules observed on those dates were due to infection by primary inoculum. Previous investigations have measured the oat crown rust latent period as being 8 d (Kochman & Brown, 1975) and between 9.6 and fifteen days (Diaz‐Lago et al., 2003). The disease severity signal was lost for these QTL before secondary inoculum would likely be able to produce disease pustules. So, it is unlikely that the observed pattern is due to genotypes being resistant to different proportions of the primary inoculum due to race structure of the pathogen.

Another possible explanation for the observation of these QTL for brief periods of time in single environments is that they are false positive identifications. However, the threshold of statistical significance used in this investigation (−log_10_(*p*) = 4.65) is appropriate considering the number of independent genetic loci in this population. A common practice to verify the QTL identified in GWA mapping is to develop and evaluate either a biparental population or near isogeneic lines which are heterogeneous for the QTL of interest. Unfortunately, QTL that exhibit environmental or temporal variation may be resistant to this type of analysis if trials fail to recreate the environmental conditions under which the QTL were first identified.

Finally, most other mapping investigations of quantitative oat crown rust resistance evaluated disease severity at a single time point (Esvelt Klos et al., 2017; Lin et al., 2014; Portyanko et al., 2005; Winkler et al., 2016). Some other investigations of quantitative disease resistance in plants have evaluated disease severity at three or four time points (Arojju et al., 2018; Jamann et al., 2015; Jamil et al., 2018; Wolfe et al., 2016; Zegeye, Rasheed, Makdis, Badebo, & Ogbonnaya, 2014). This investigation evaluated disease severity at fifteen, eight, and seven time points in the three environments; making it one of the most temporally dense plant disease investigations to date. Some researchers may dismiss some of the QTL identified in the investigation because the genetic signal is present for only a single or small number of time points. However, it is possible that many other disease severity QTL identified in other investigations which measured disease severity at a single time point were also similarly short‐lived and may have escaped scrutiny due to a lack of information. These additional observations may currently be labor intensive to collect but could potentially be completed more easily by the development of high‐throughput phenotyping techniques.

## AUTHOR CONTRIBUTIONS

I.G. McNish designed the investigation, managed the field plots, scored the plant traits, analyzed the data, and wrote the manuscript. C.M. Zimmer edited the manuscript and provided critiques of the results and discussion. A.Q. Susko edited the manuscript and provided critiques of the results and discussion. D.J. Heuschele edited the manuscript, provided critiques of the analysis and discussion, and organized genotyping activities. T. Tiede collected the Founder population, submitted lines for inclusion in the POGI population, and wrote parts of the manuscript related to those activities. A.J. Case demonstrated rust scoring technique to I.G. McNish, wrote the code to produce consensus genotype calls for the POGI population, and organized genotyping activities. K.P. Smith advised the other coauthors on all aspects of the project, edited the manuscript, and obtained funding for the research.

## CONFLICT OF INTEREST DISCLOSURE

The authors declare no conflicts of interest.

## Supporting information

Supplemental Figure S1: Founder population scree plot.

Supplemental Figure S2: plot of the k‐means gap statistic of University of Minnesota Oat Founder population principal component analysis data from k = 1 to k = 10.

Supplemental Figure S3: Heatmaps of pairwise r^2^ and linkage block cluster analysis of each linkage group from Bekele *et al*. (2018).

Supplemental Figure S4: Correlation plots for each trial. Scatter plots for each pair of variables are below the axis.

Supplemental Figure S5: Manhattan and quantile‐quantile (Q‐Q) plots for all genome‐wide association models.

Supplemental Table S1: Pairwise r^2^ matrices for each Bekele *et al*. (2018) linkage group. R^2^ values are above the diagonal.

Supplemental Table S2: Linkage block assignments for each marker determined by adjacency‐constrained hierarchical agglomerative clustering.

Supplemental Table S3: Full genome‐wide association mapping results for each genome‐wide association model.

Supplemental Figure S1: Founder population scree plot.
Supplemental Figure S2: plot of the k‐means gap statistic of University of Minnesota Oat Founder population principal component analysis data from k = 1 to k = 10.
Supplemental Figure S3: Heatmaps of pairwise r^2^ and linkage block cluster analysis of each linkage group from Bekele *et al*. (2018).
Supplemental Figure S4: Correlation plots for each trial. Scatter plots for each pair of variables are below the axis.
Supplemental Figure S5: Manhattan and quantile‐quantile (Q‐Q) plots for all genome‐wide association models.
Supplemental Table S1: Pairwise r^2^ matrices for each Bekele *et al*. (2018) linkage group. R^2^ values are above the diagonal.
Supplemental Table S2: Linkage block assignments for each marker determined by adjacency‐constrained hierarchical agglomerative clustering.
Supplemental Table S3: Full genome‐wide association mapping results for each genome‐wide association model.

## Data Availability

Genotypic and phenotypic data is available from the T3/Oat database (https://triticeaetoolbox.org/oat/). The Founder population can be downloaded using the ‘UMN Founder Population’ preselected line set. All data and analysis scripts can be downloaded from GitHub (https://github.umn.edu/mcnis003/2019_plant_genome_cr_gwas.git).
