# Supplementary material for: Mapping crown rust resistance at multiple time points in elite oat germplasm
Source: Plant Genome. 2020 Apr 2;13(1):e20007. doi: 10.1002/tpg2.20007 (PMC12806915; doi:10.1002/tpg2.20007)
Supplement: Supplementary file 1 [file TPG2-13-e20007-s001.pdf]

2017 early trial

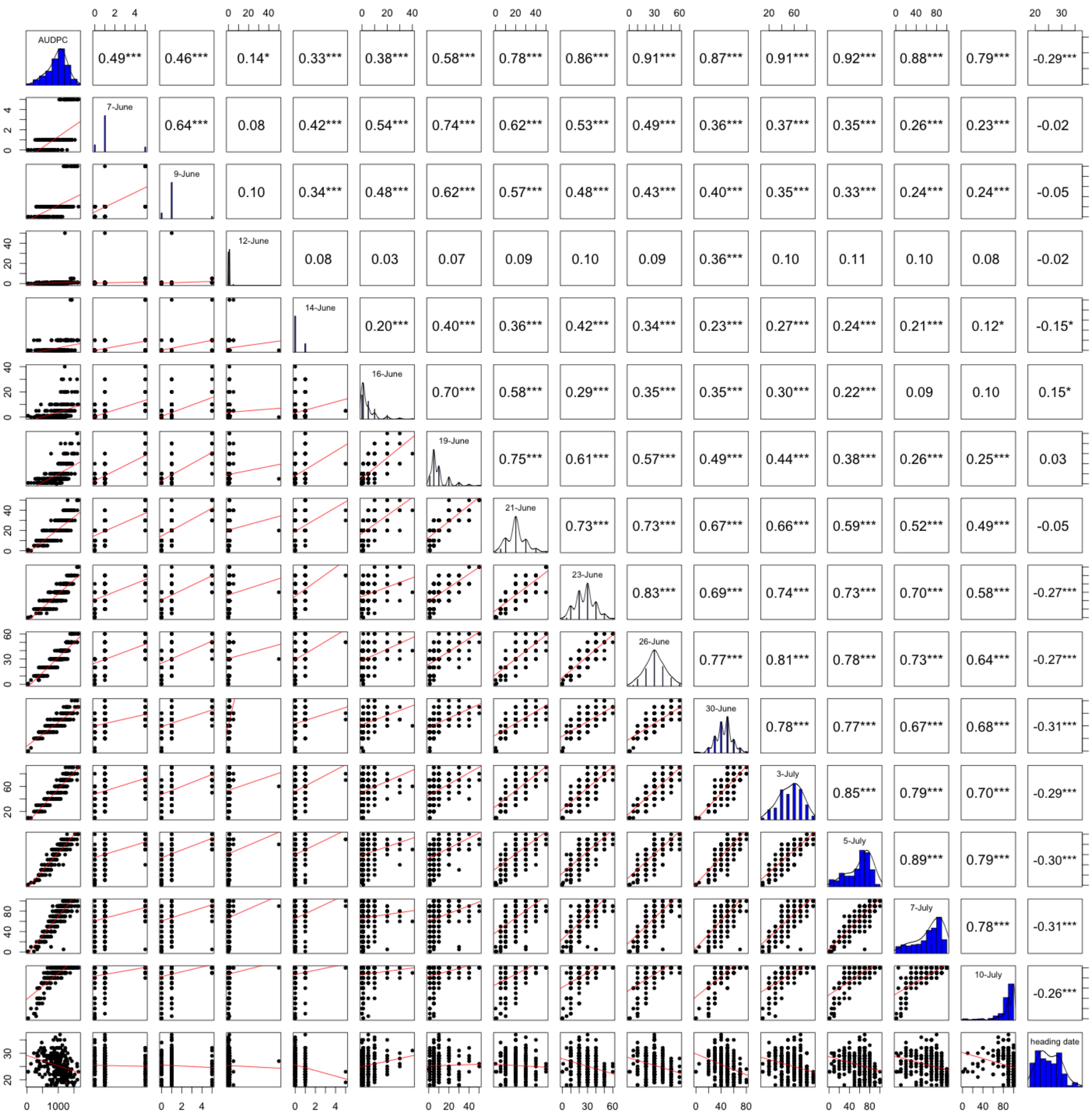

Figure 1:

2017 late trial

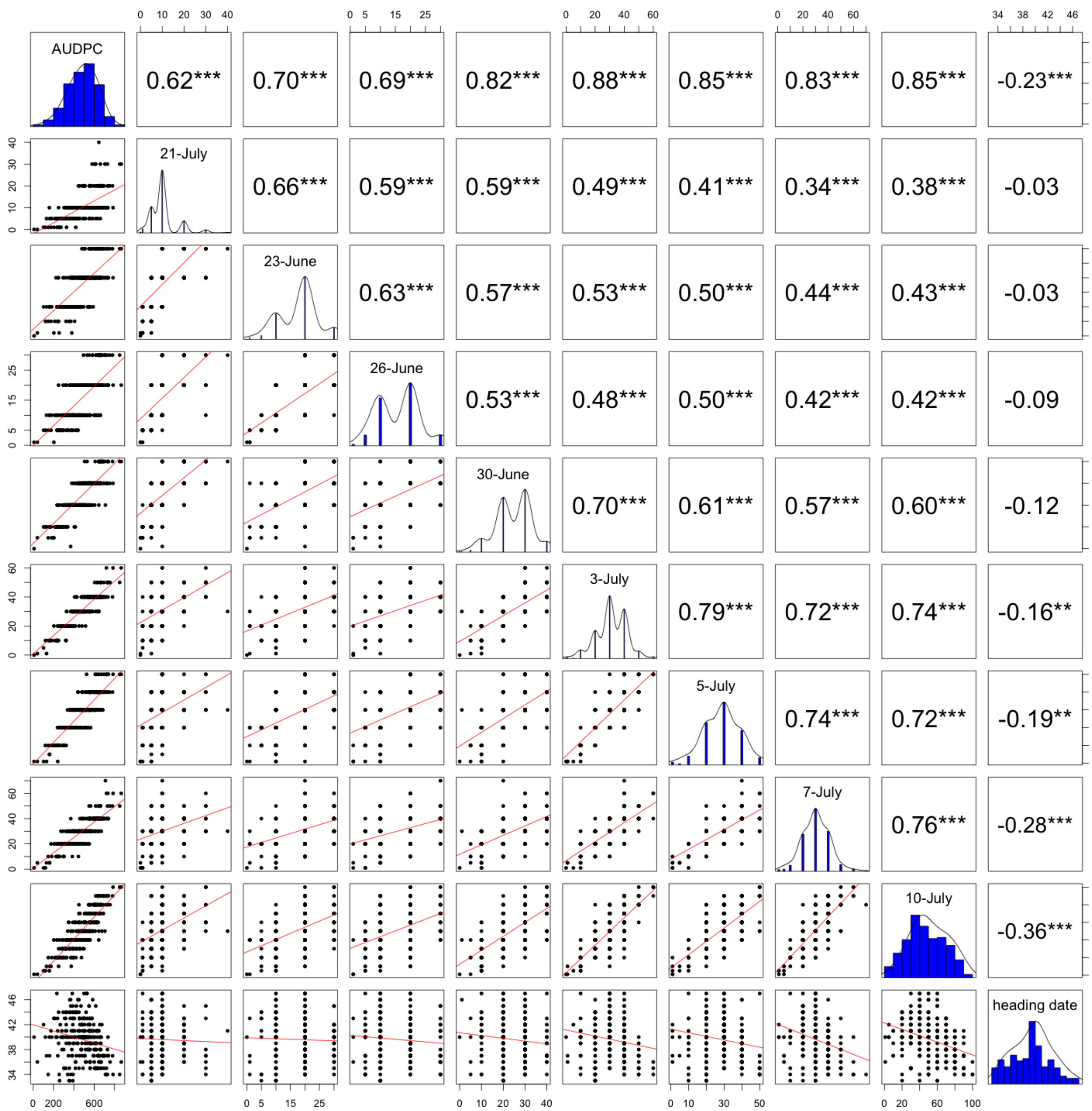

Figure 2:

2018 trial

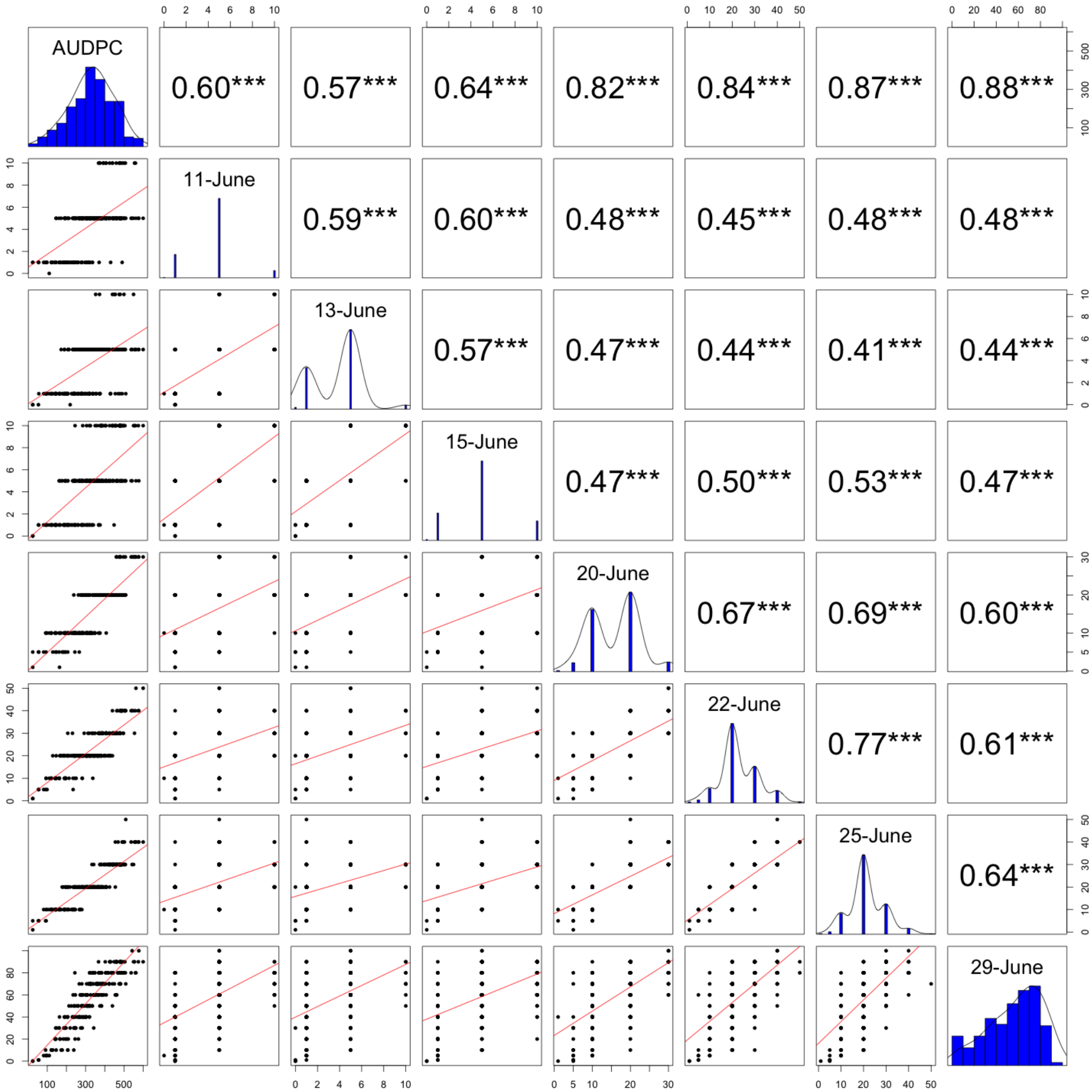

Figure 3:
