# Supplementary material for: Mapping crown rust resistance at multiple time points in elite oat germplasm
Source: Plant Genome. 2020 Apr 2;13(1):e20007. doi: 10.1002/tpg2.20007 (PMC12806915; doi:10.1002/tpg2.20007)

2017 early trial AUDPC

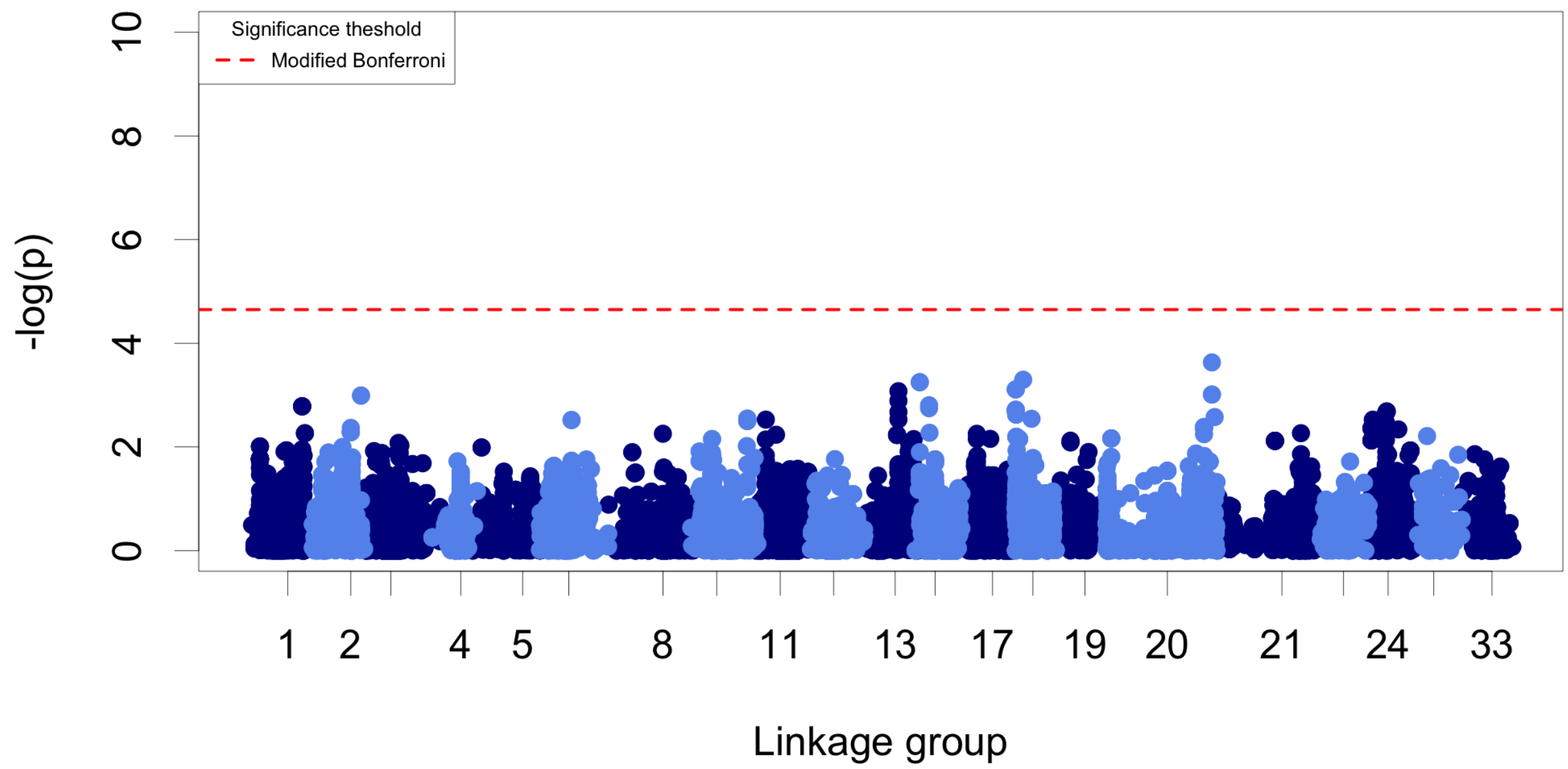

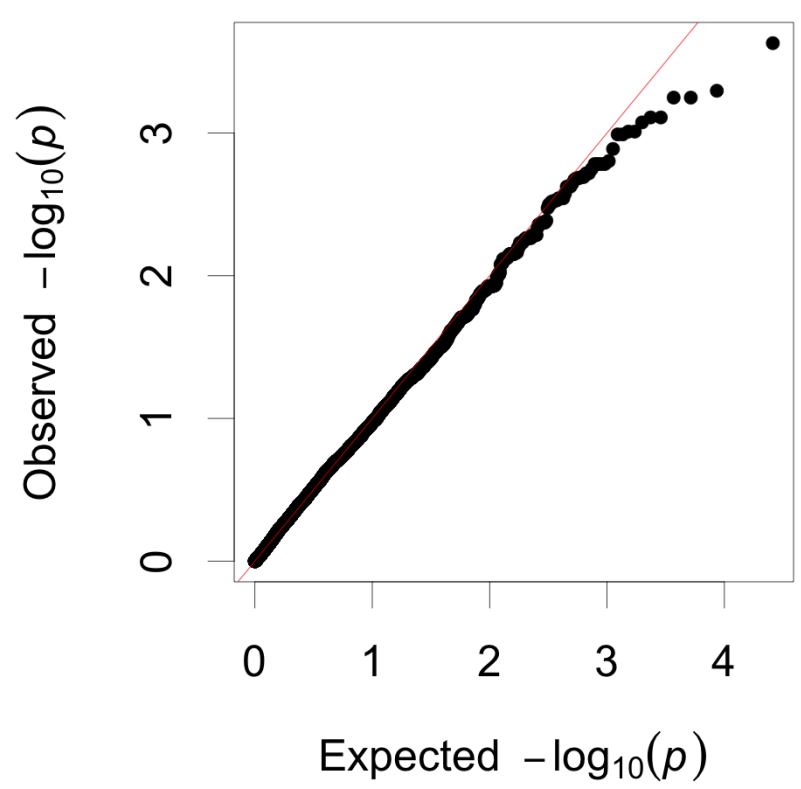

2017 early trial 7-Jun-17 observation

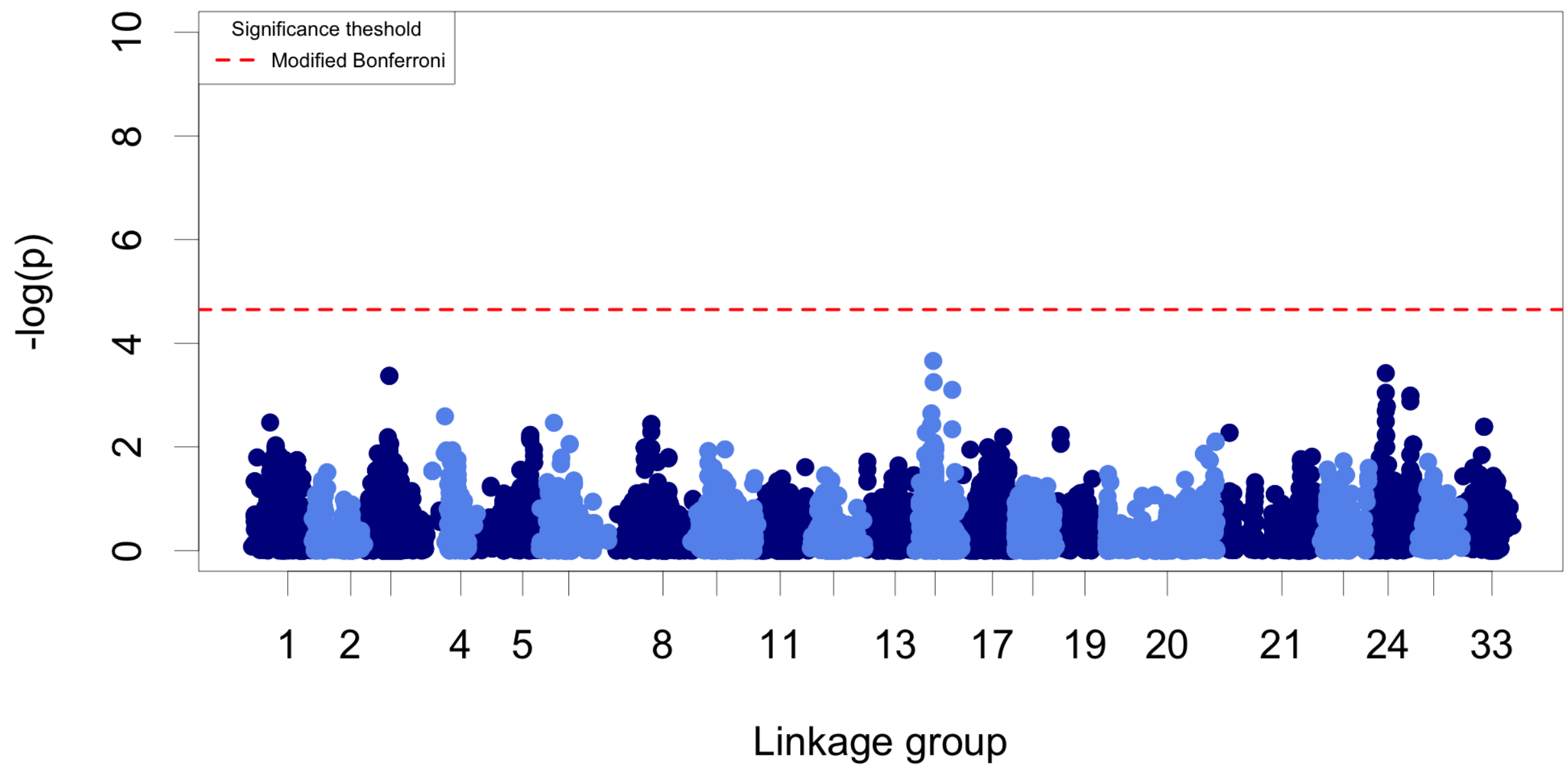

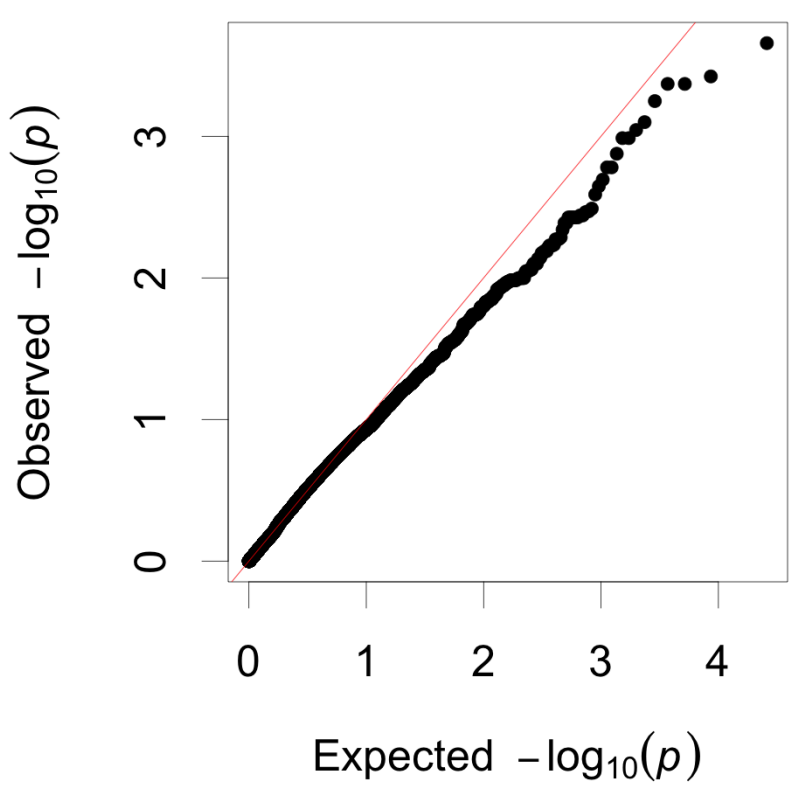

2017 early trial 9-Jun-17 observation

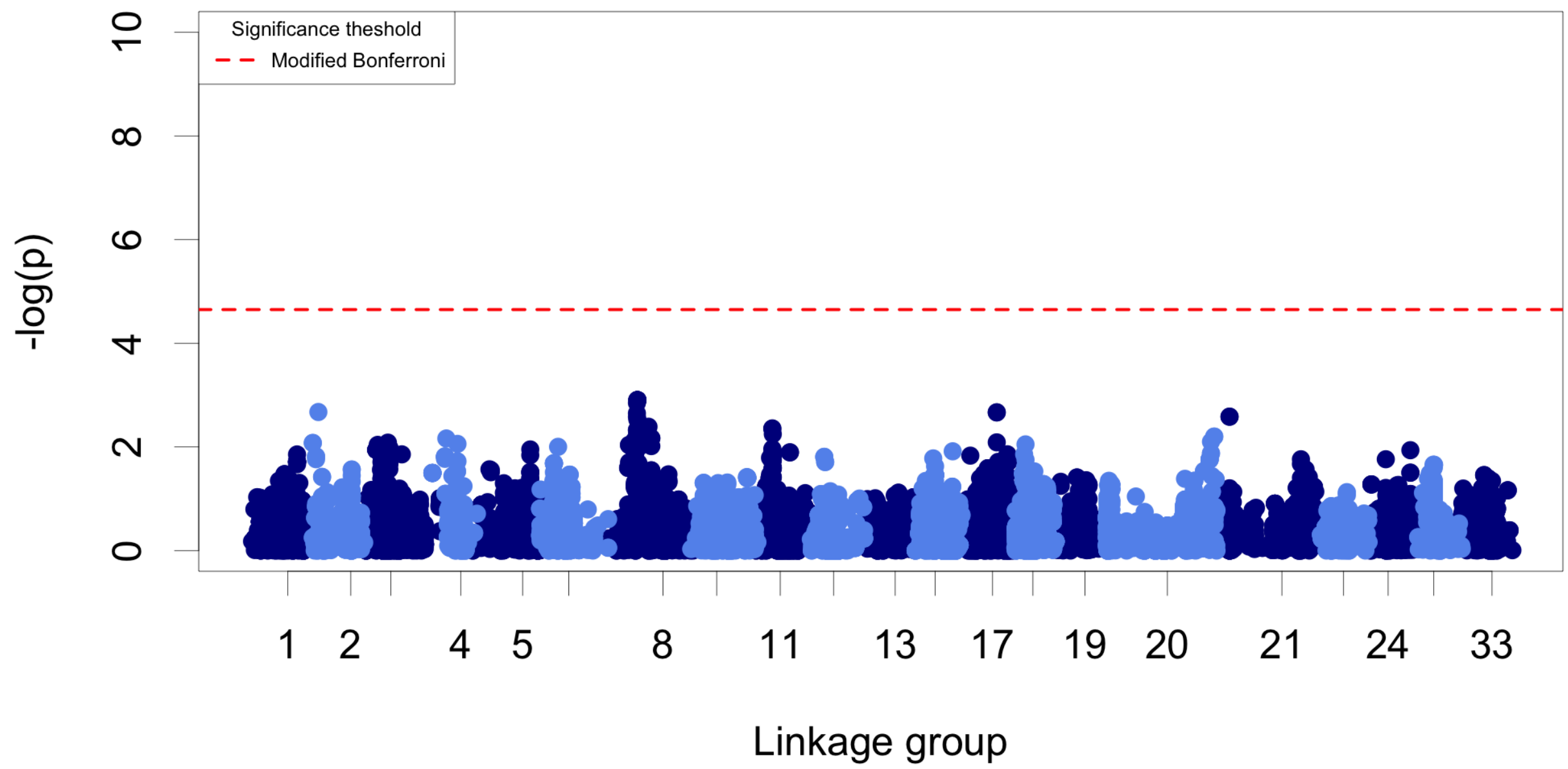

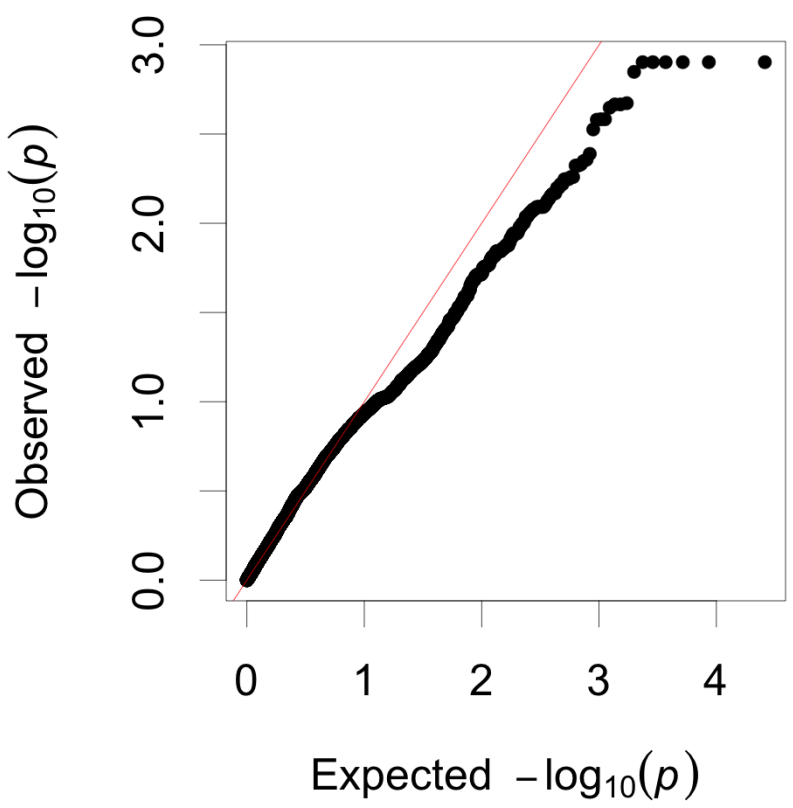

2017 early trial 12-Jun-17 observation

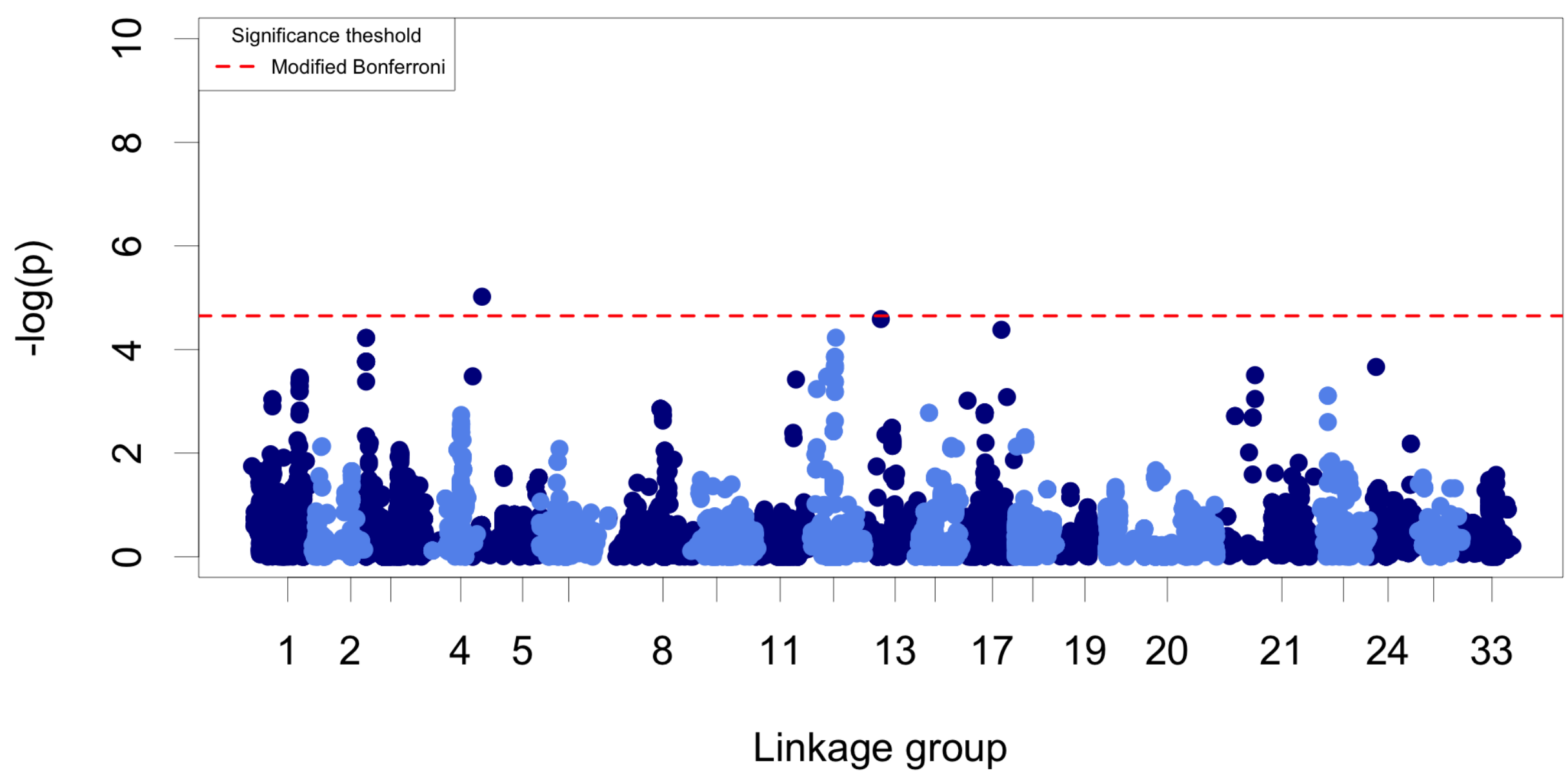

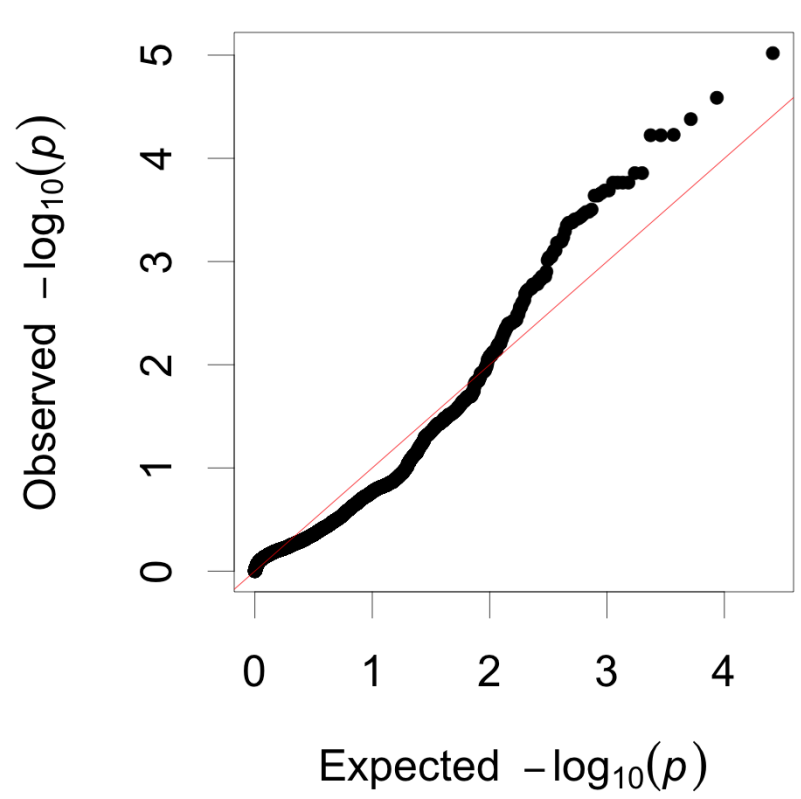

2017 early trial 14-Jun-17 observation

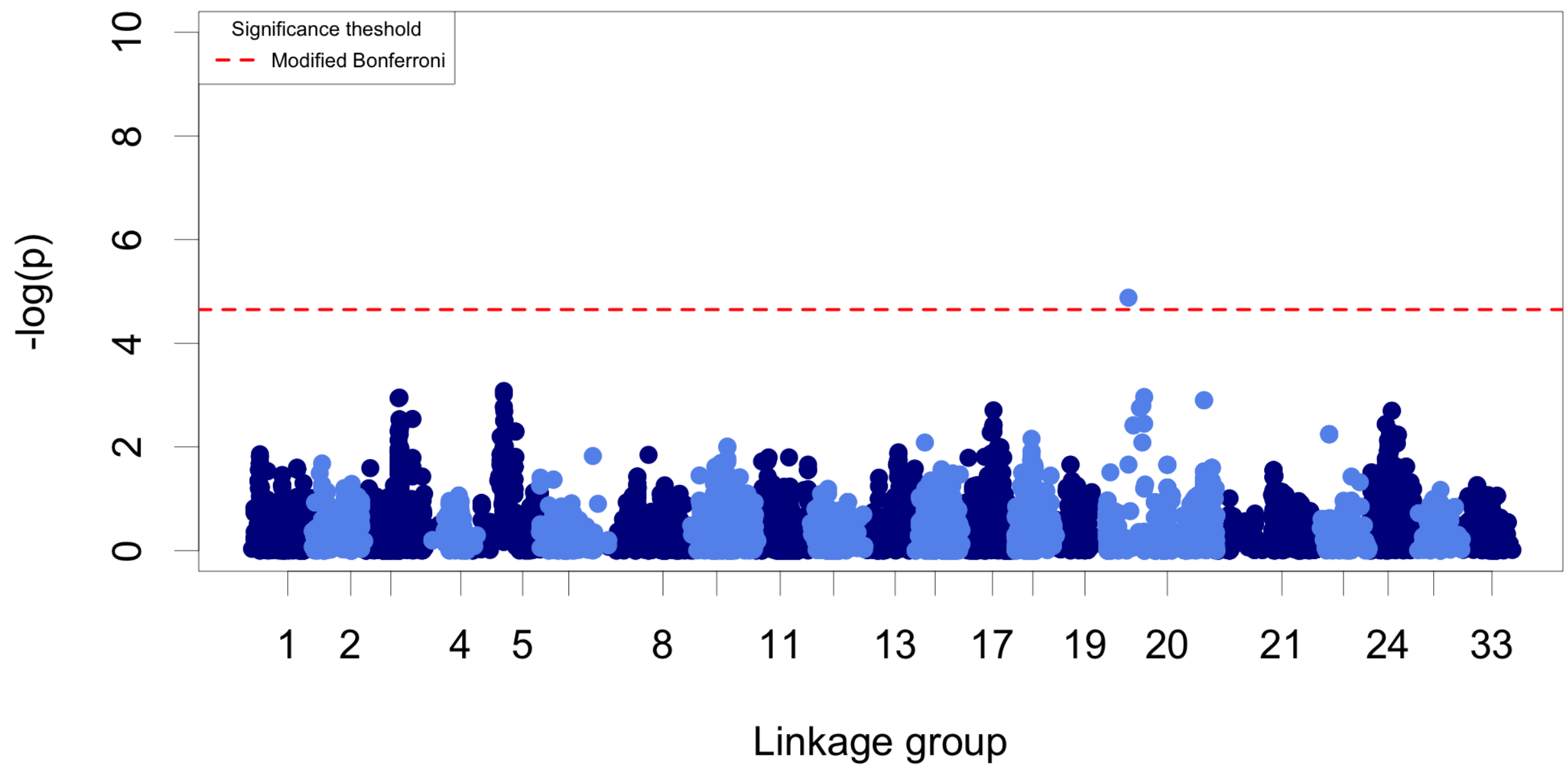

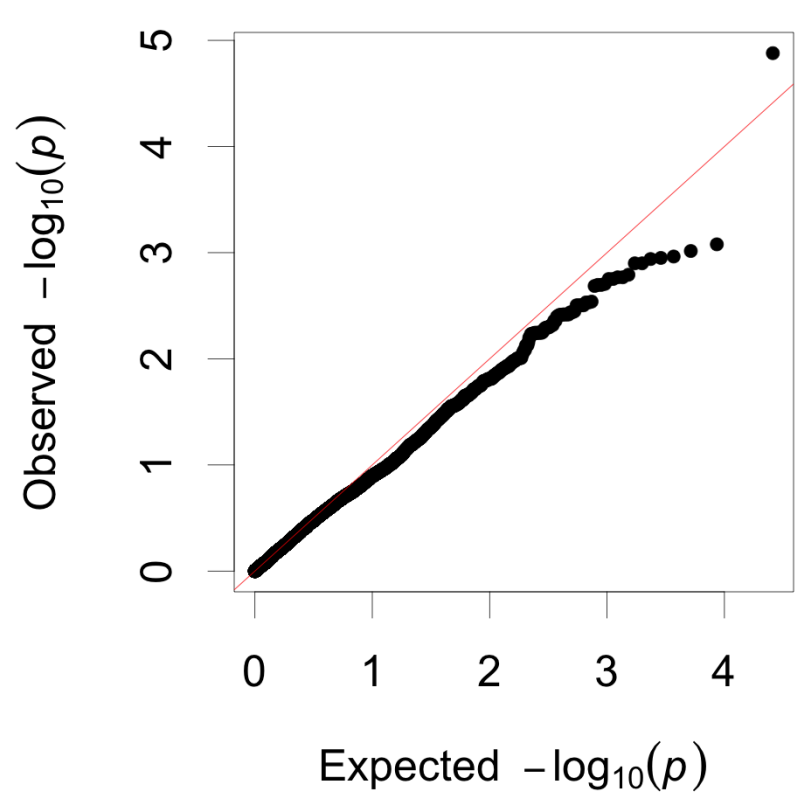

2017 early trial 16-Jun-17 observation

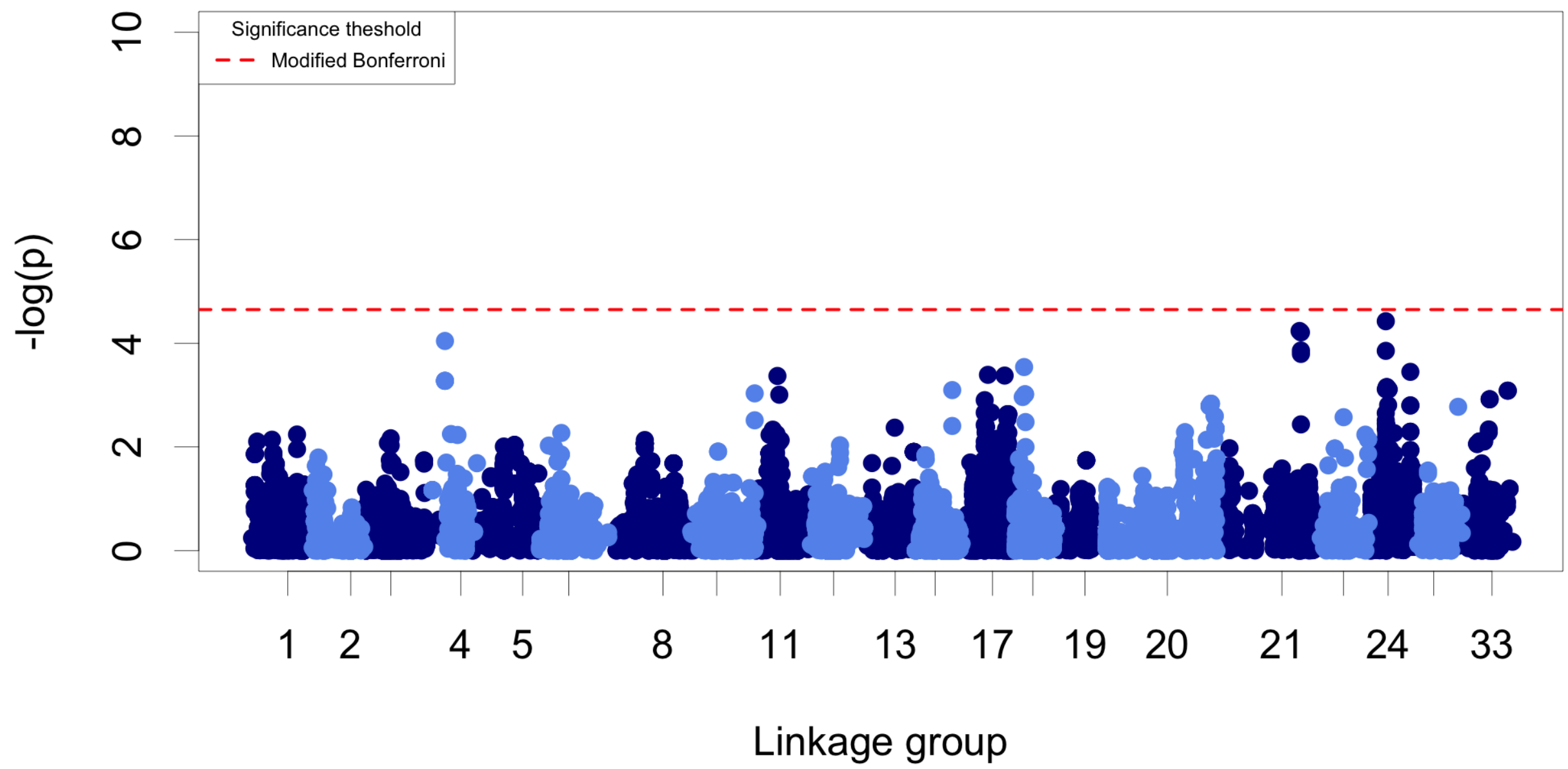

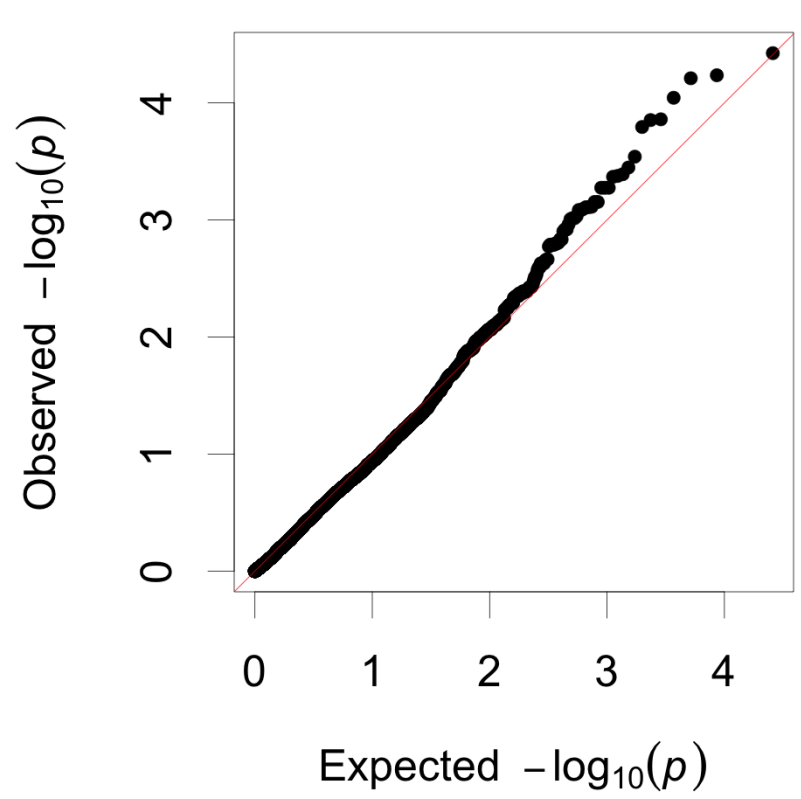

2017 early trial 19-Jun-17 observation

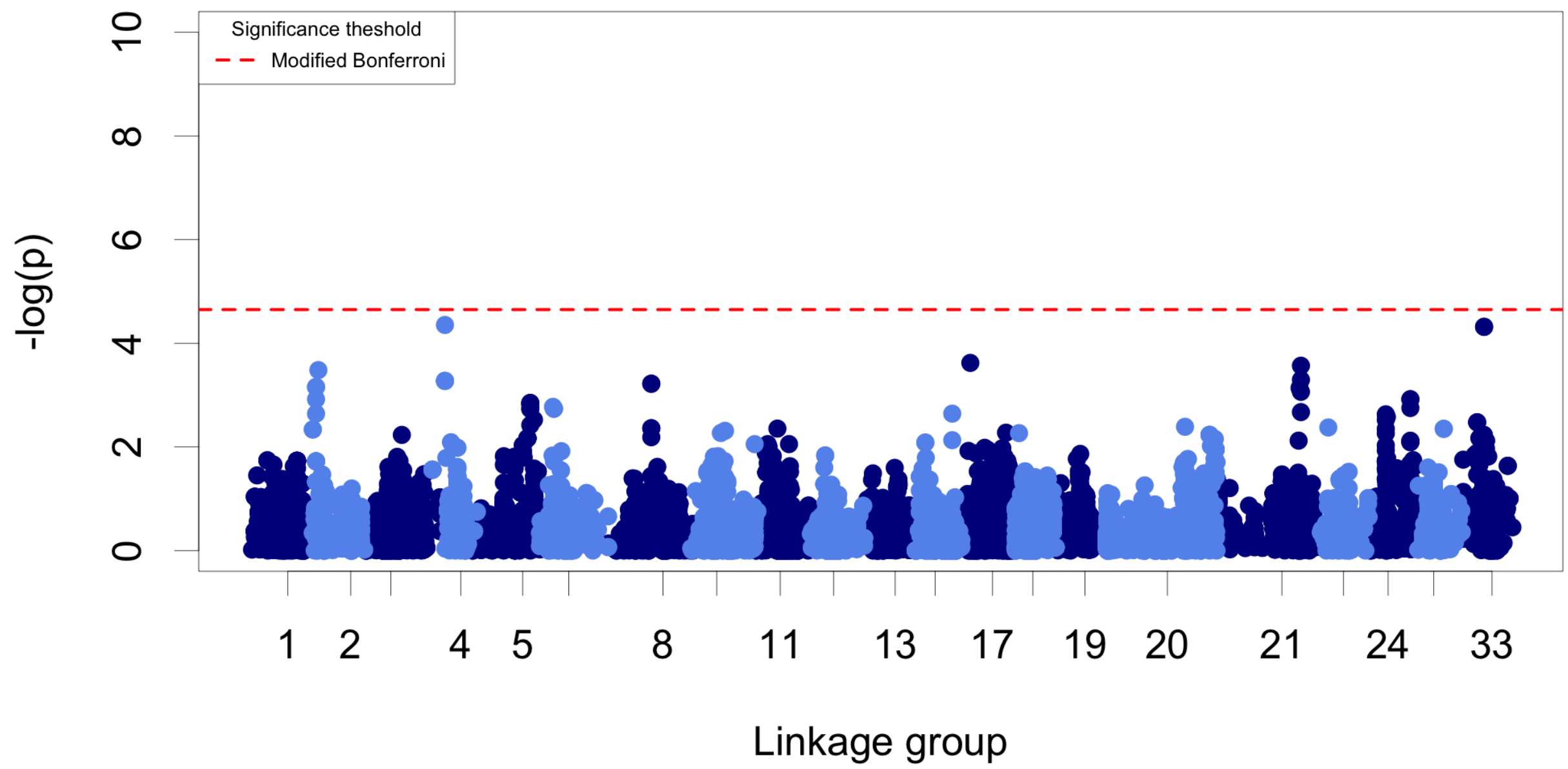

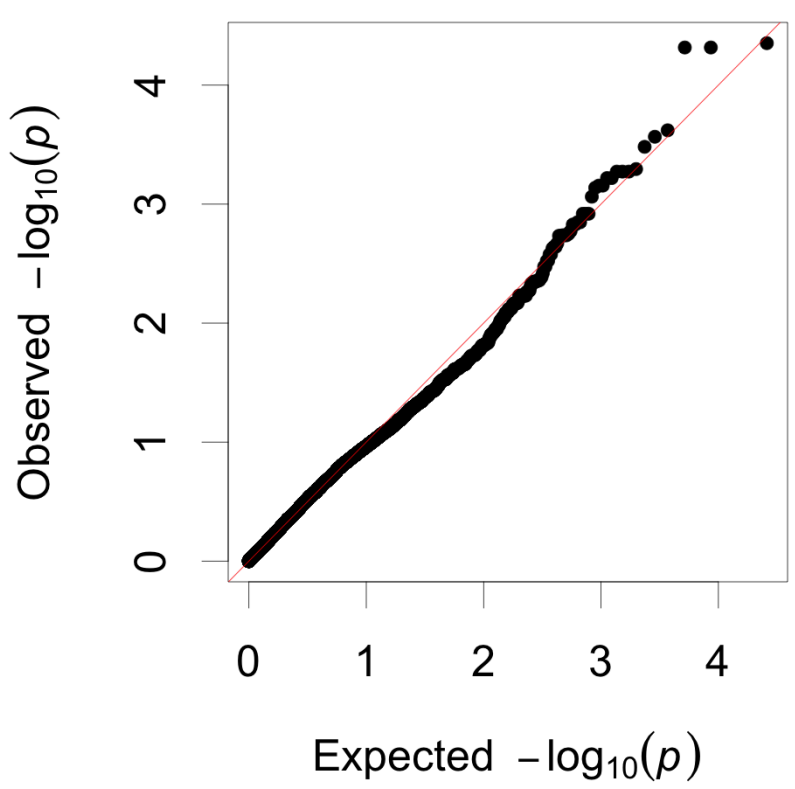

2017 early trial 21-Jun-17 observation

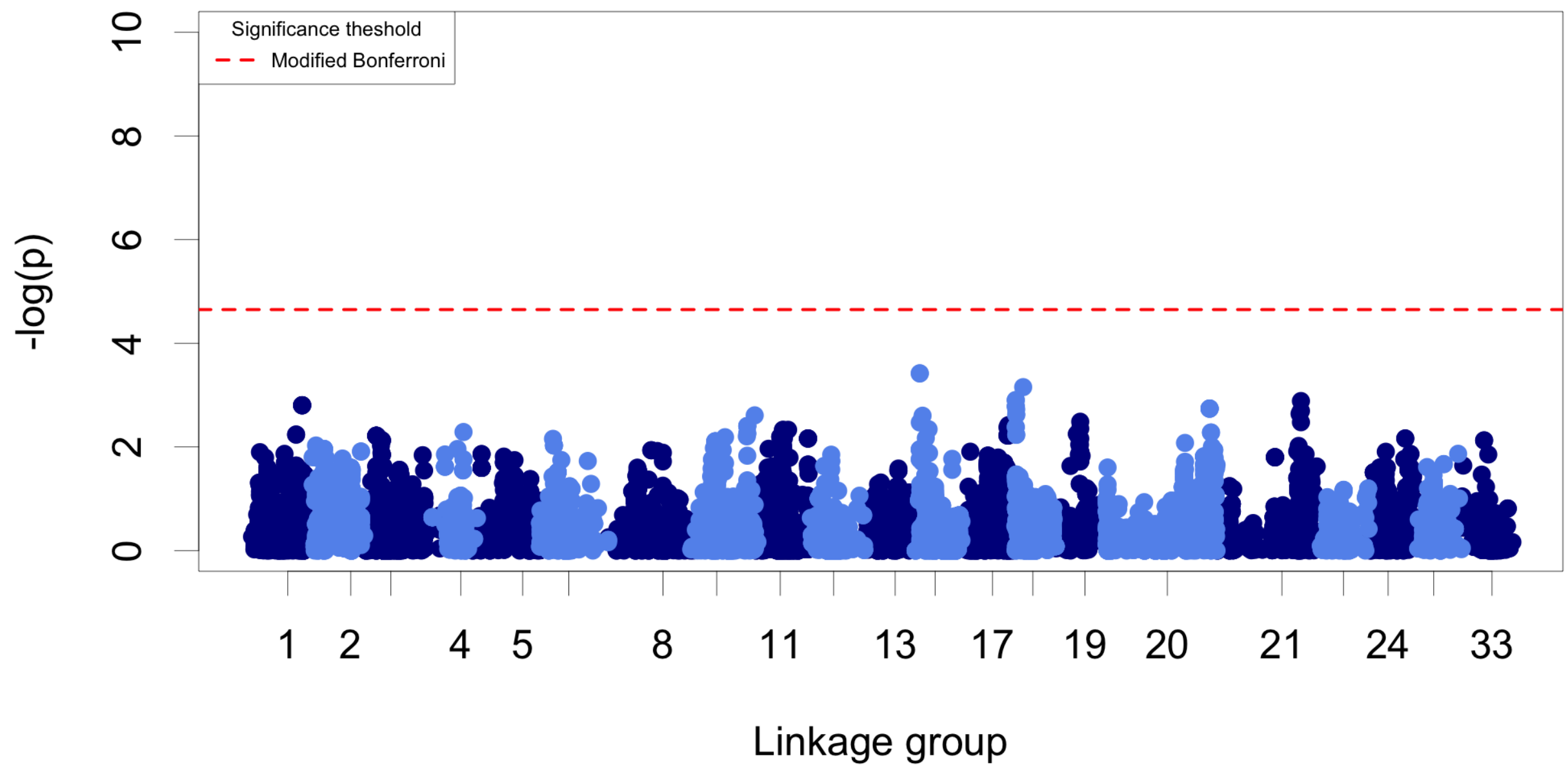

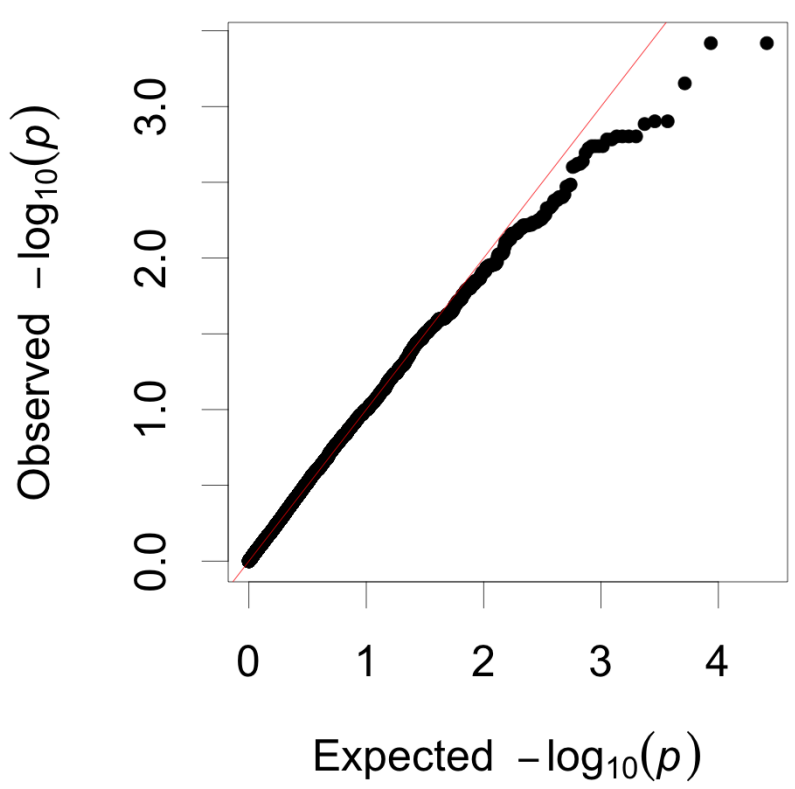

2017 early trial 23-Jun-17 observation

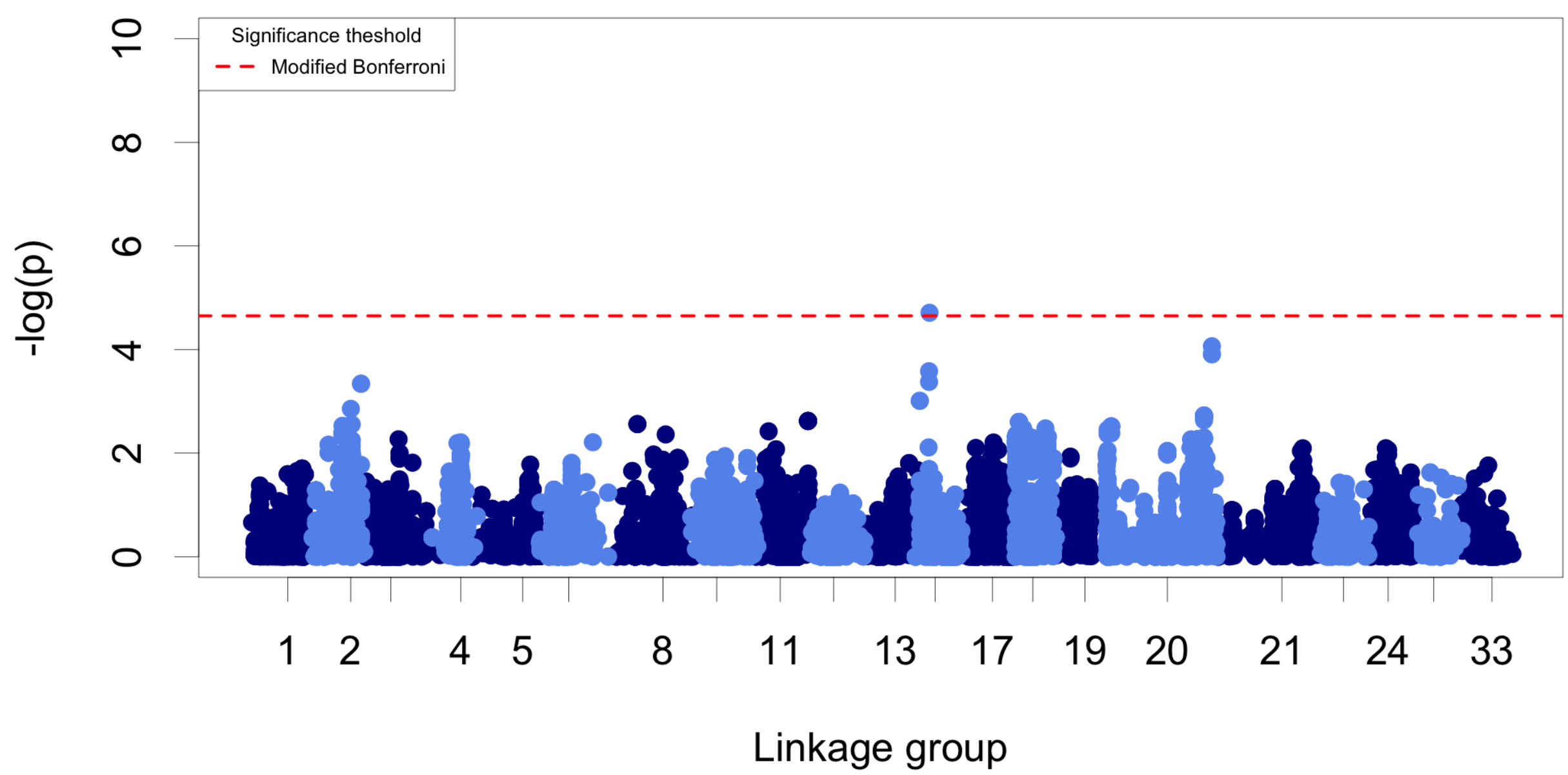

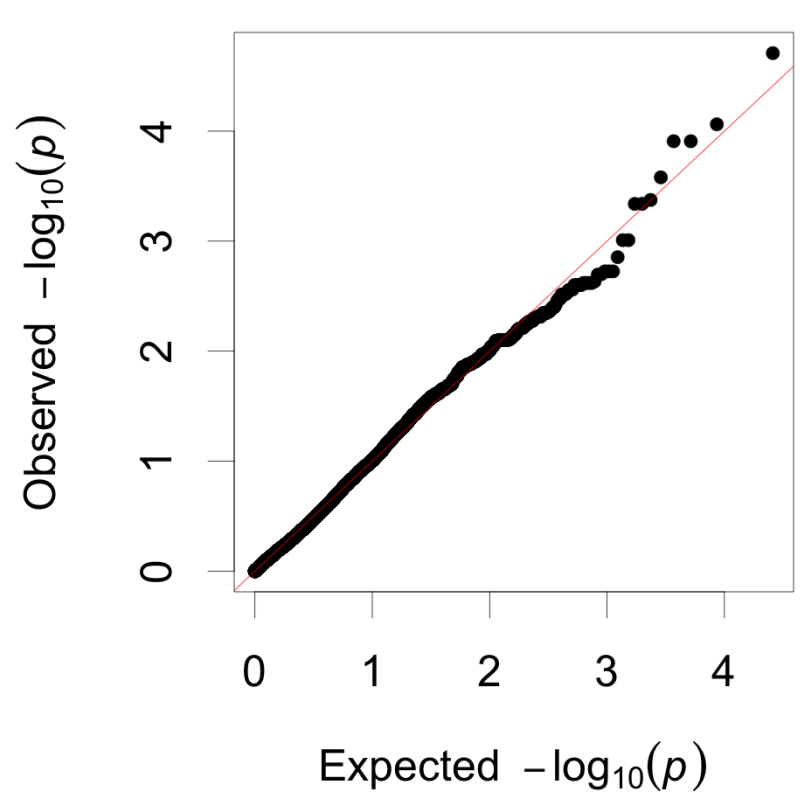

2017 early trial 26-Jun-17 observation

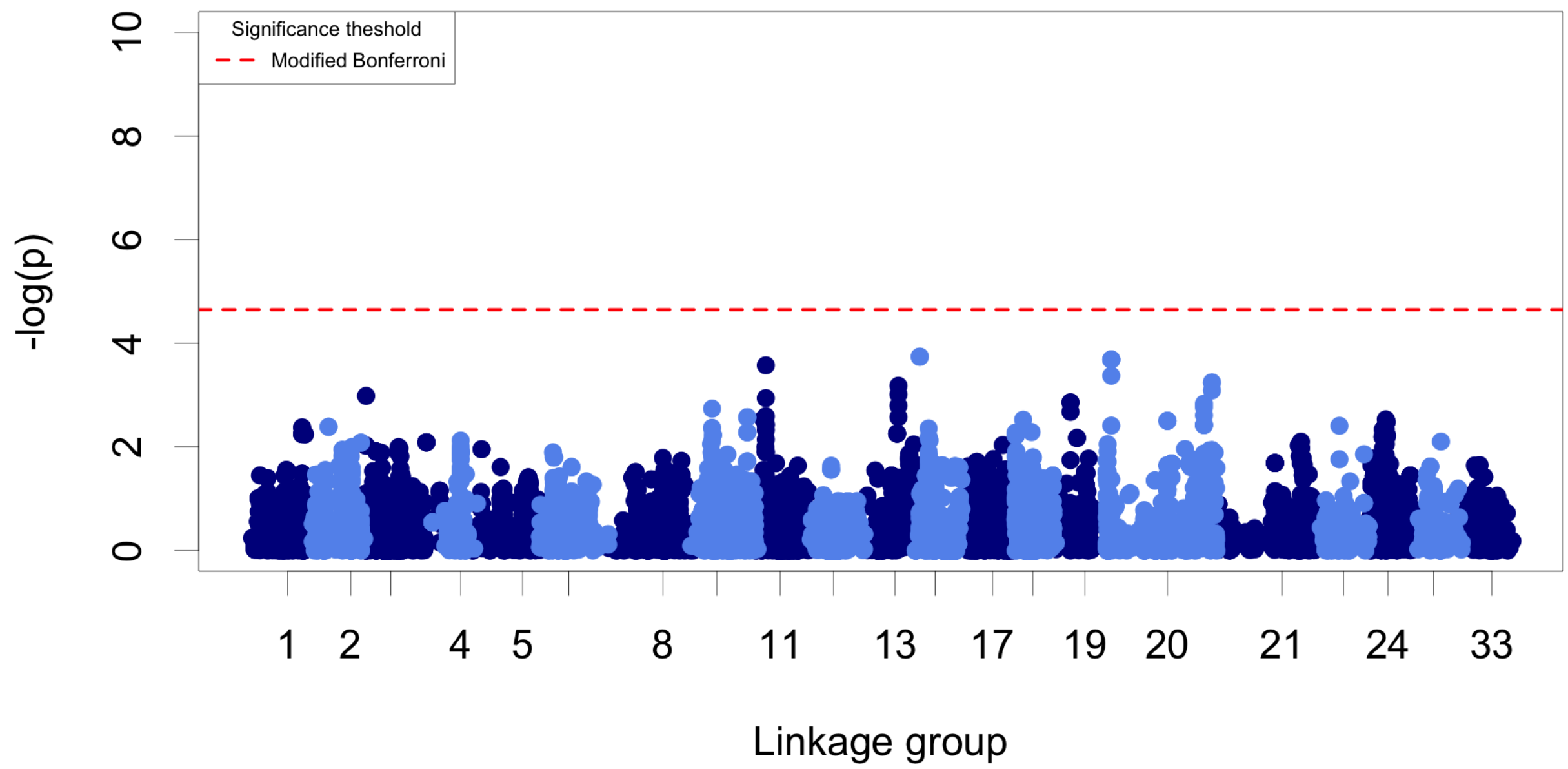

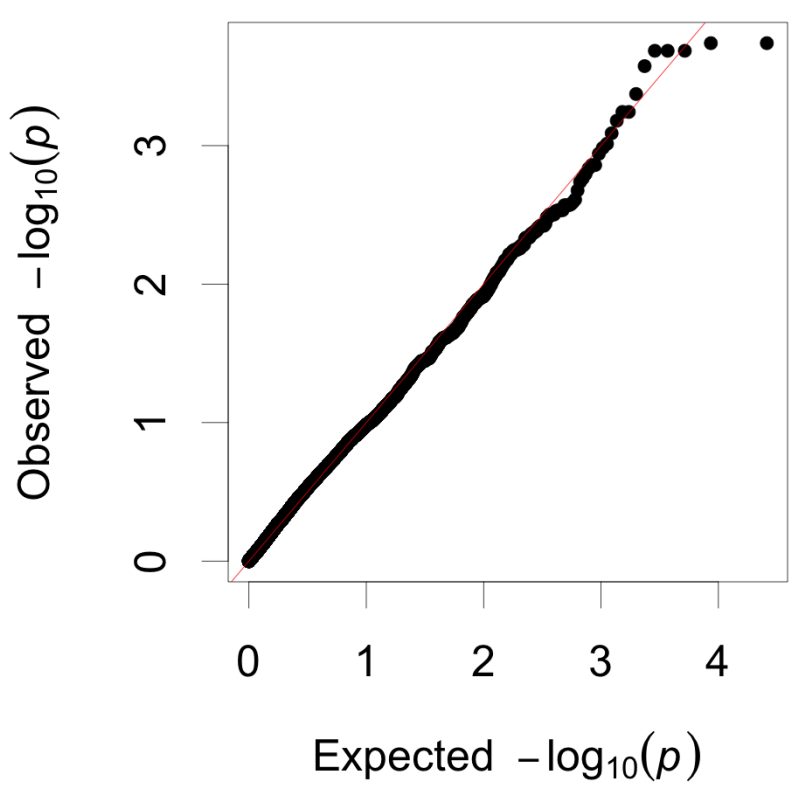

2017 early trial 30-Jun-17 observation

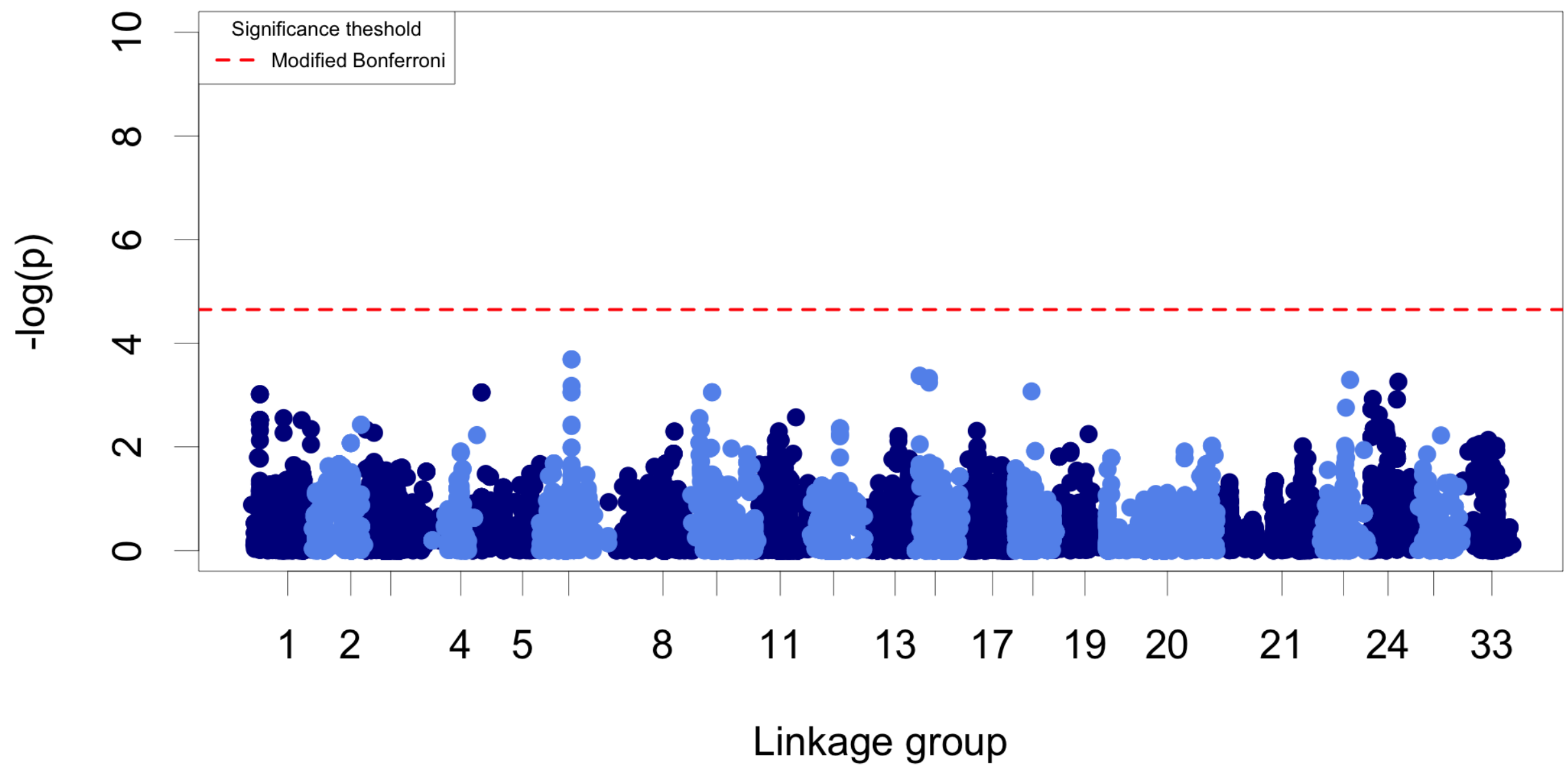

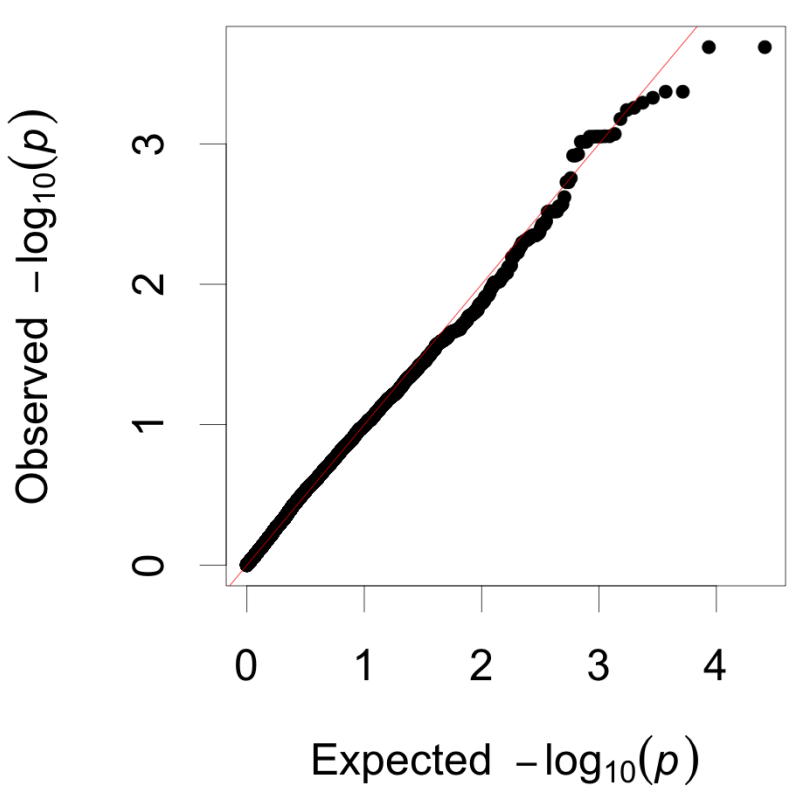

2017 early trial 3-Jul-17 observation

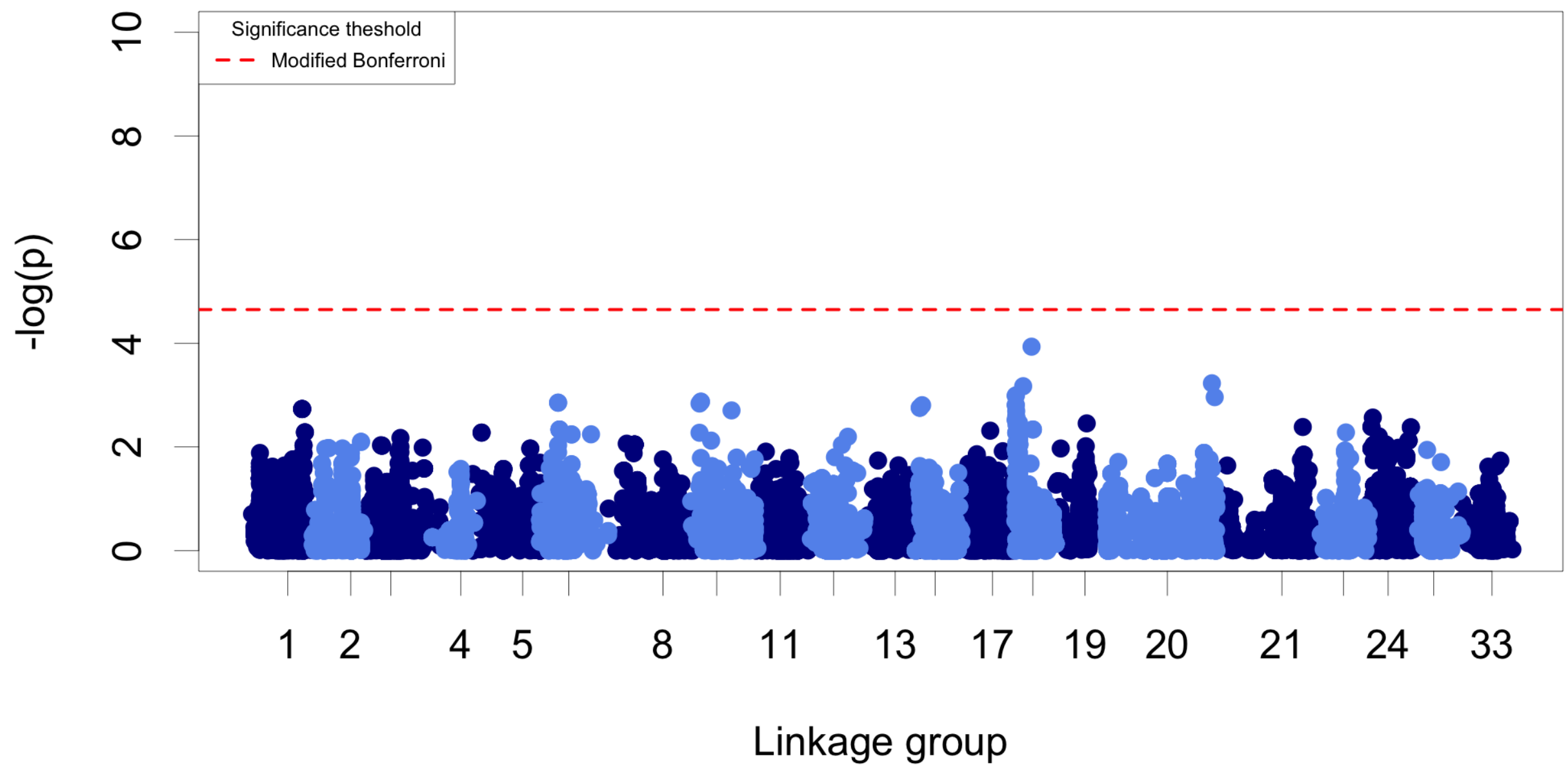

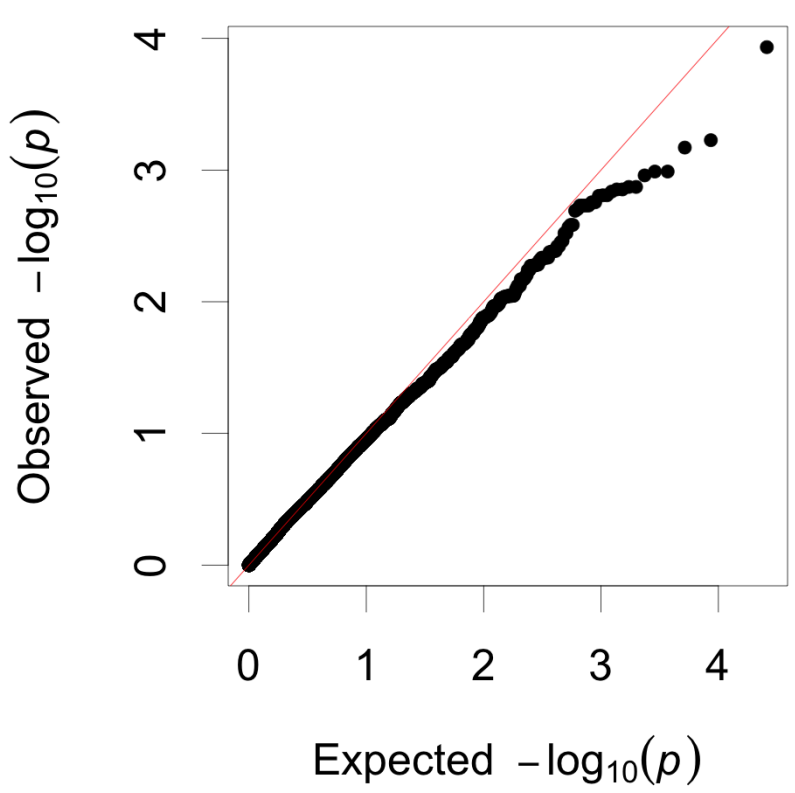

2017 early trial 5-Jul-17 observation

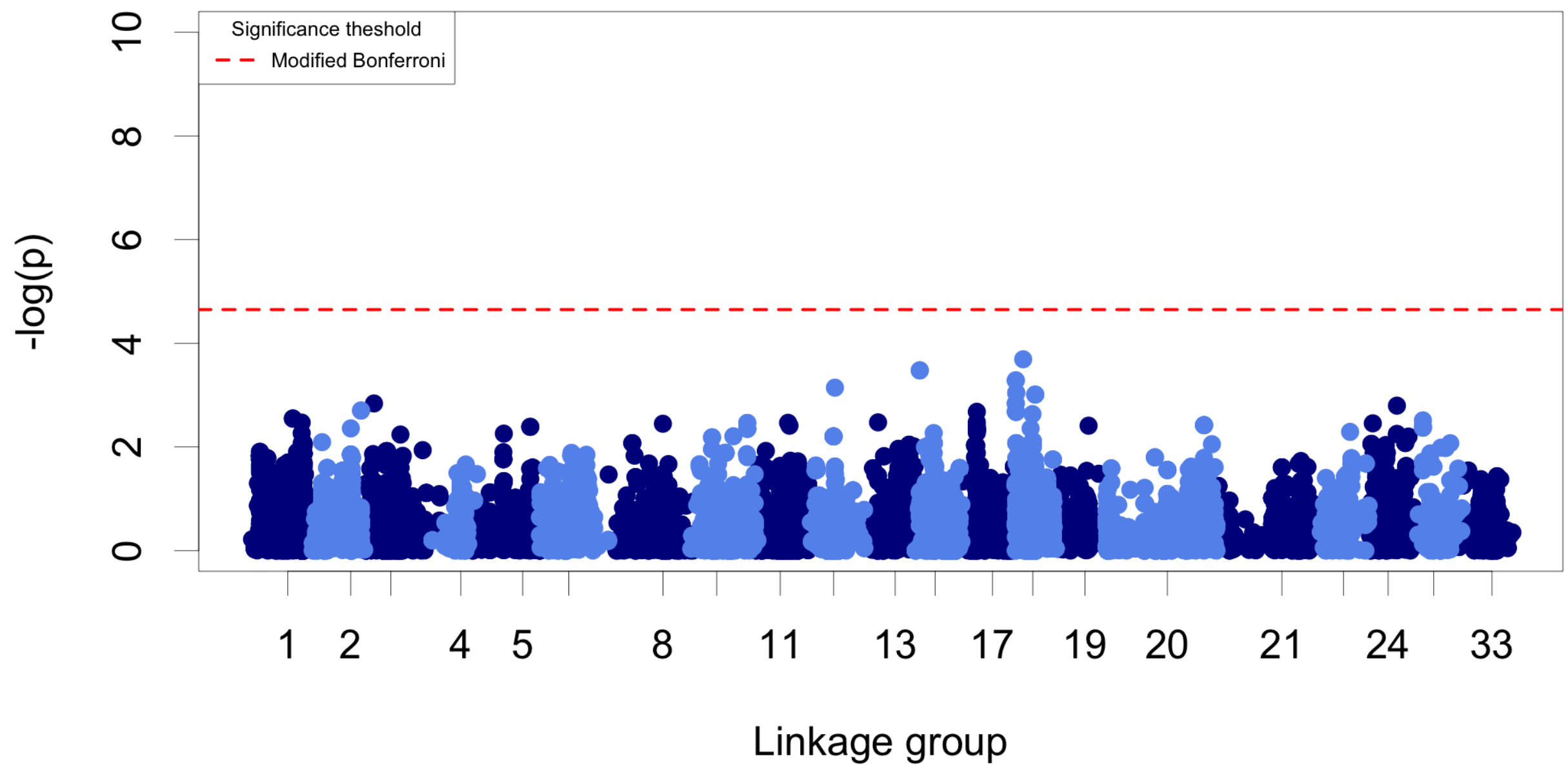

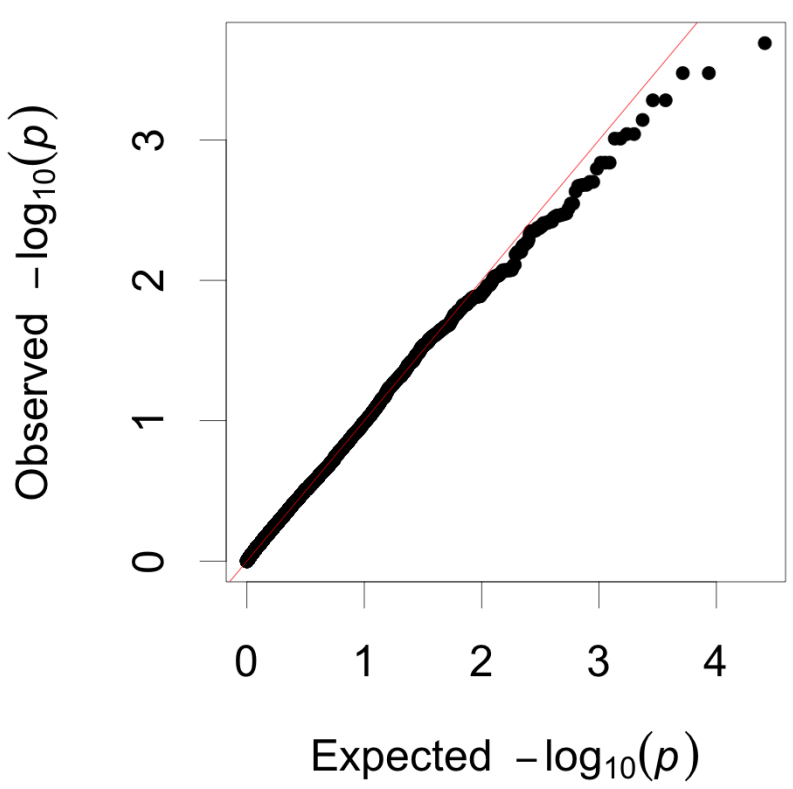

2017 early trial 7-Jul-17 observation

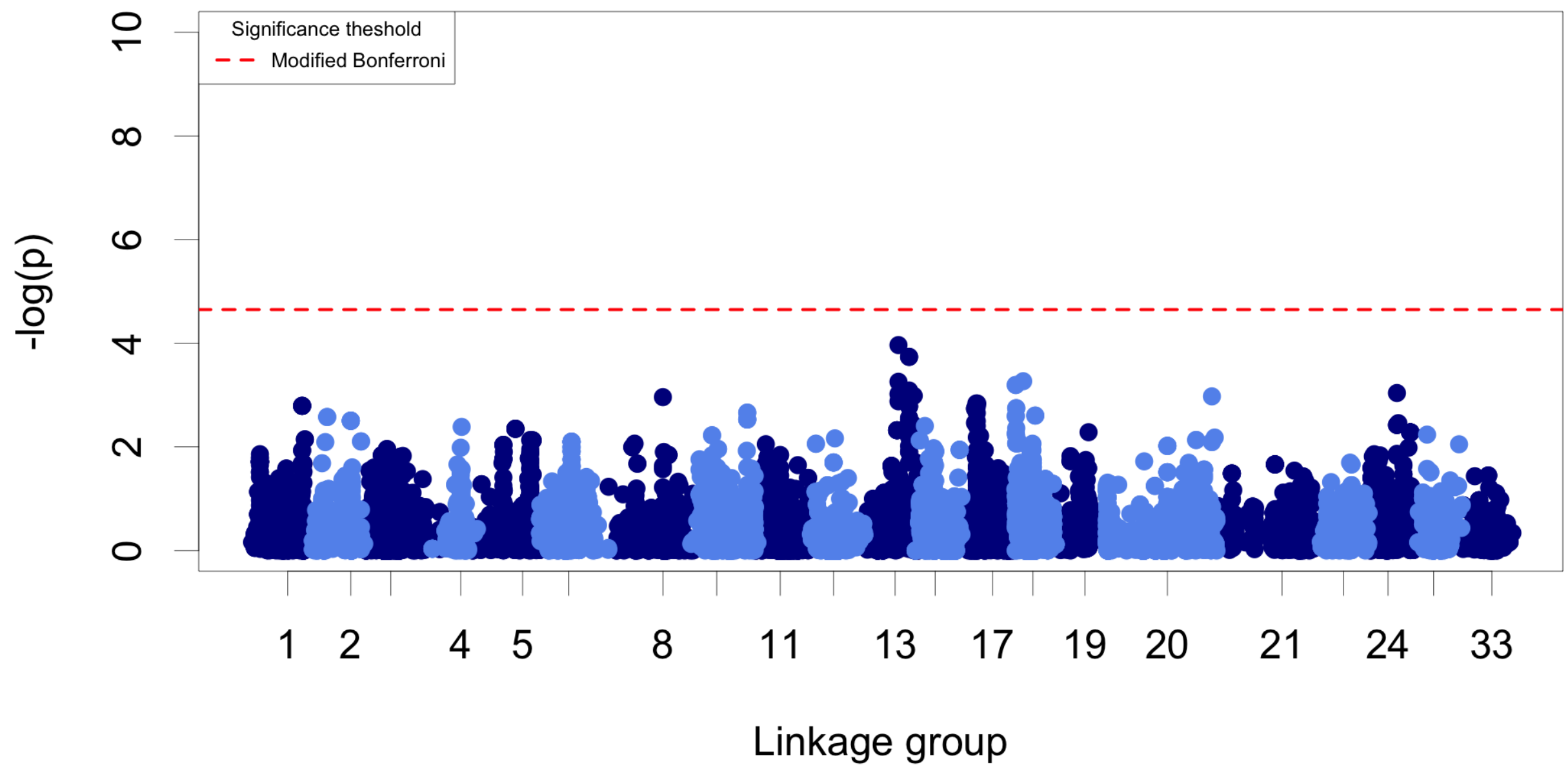

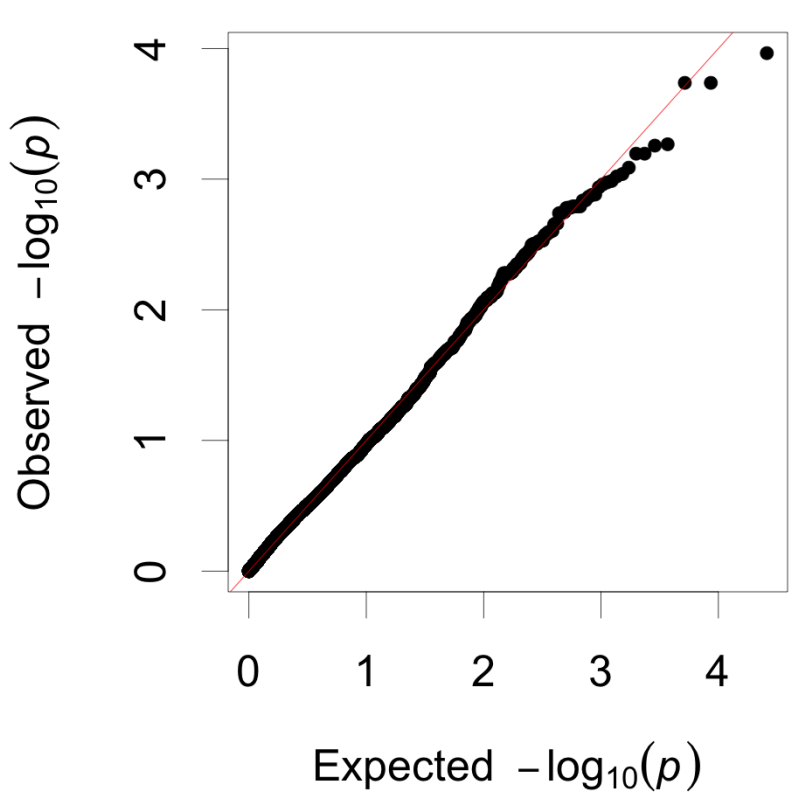

2017 early trial 10-Jul-17 observation

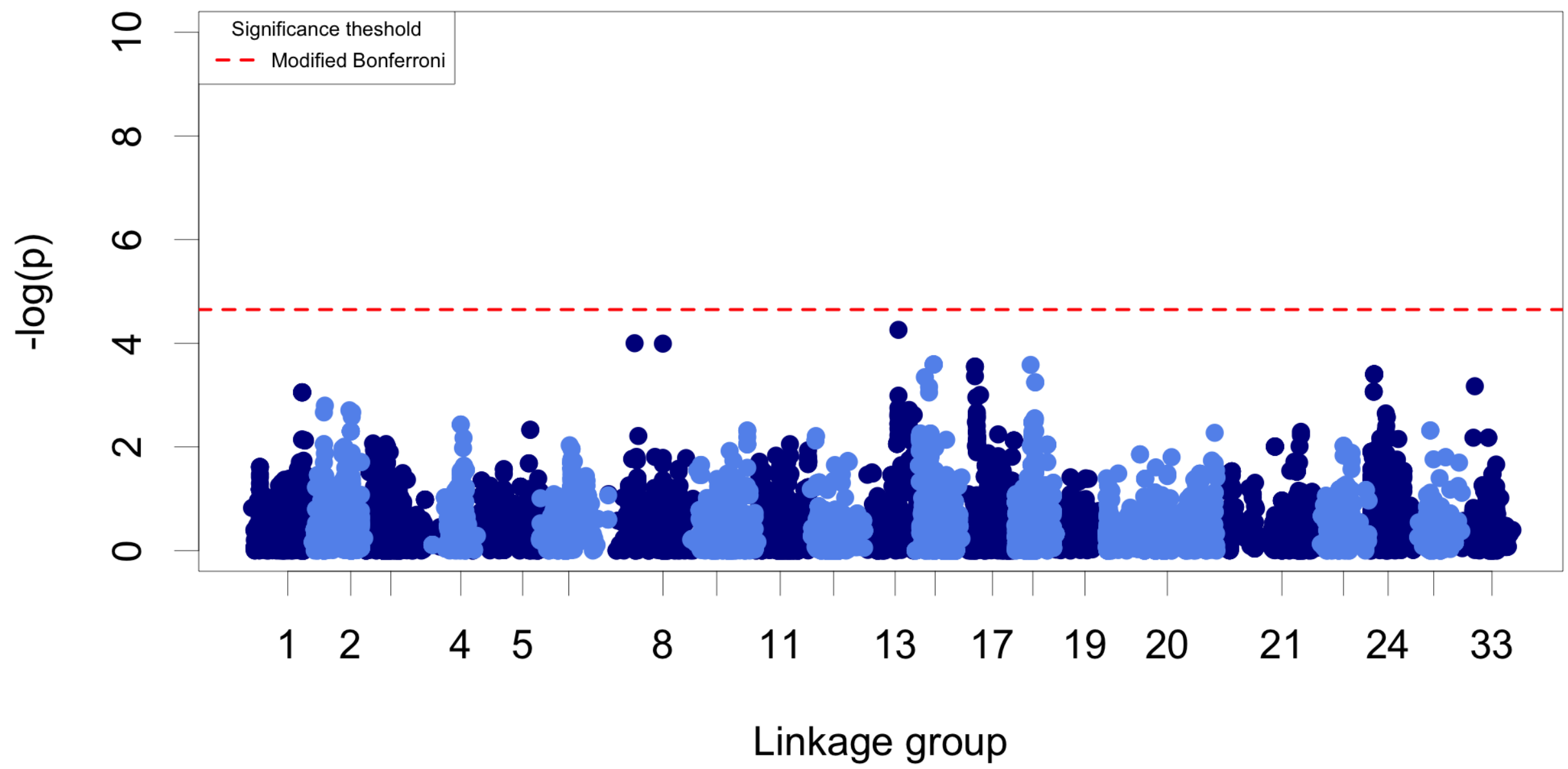

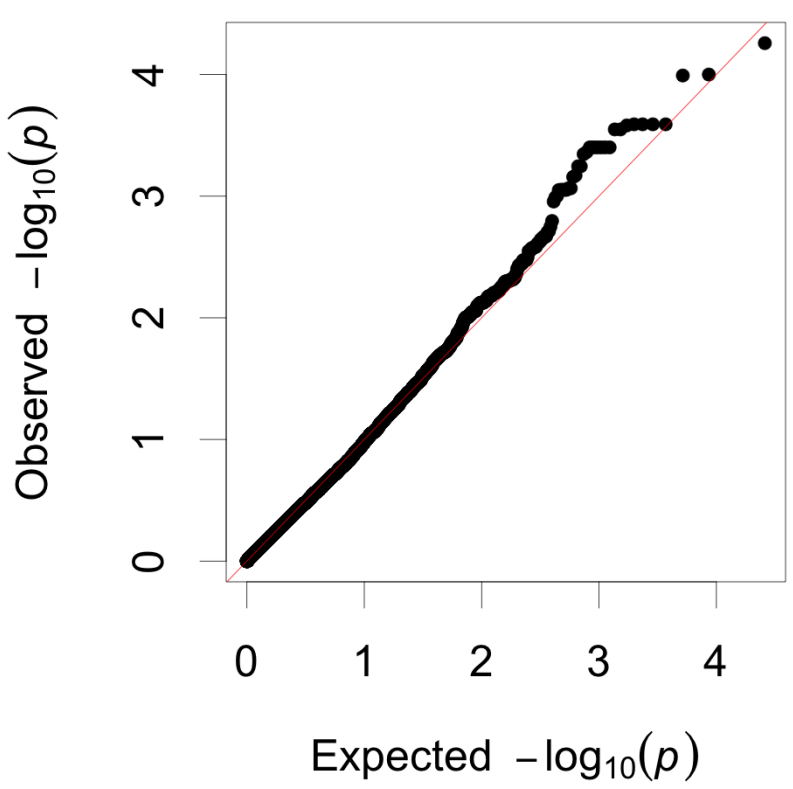

2017 early trial heading date

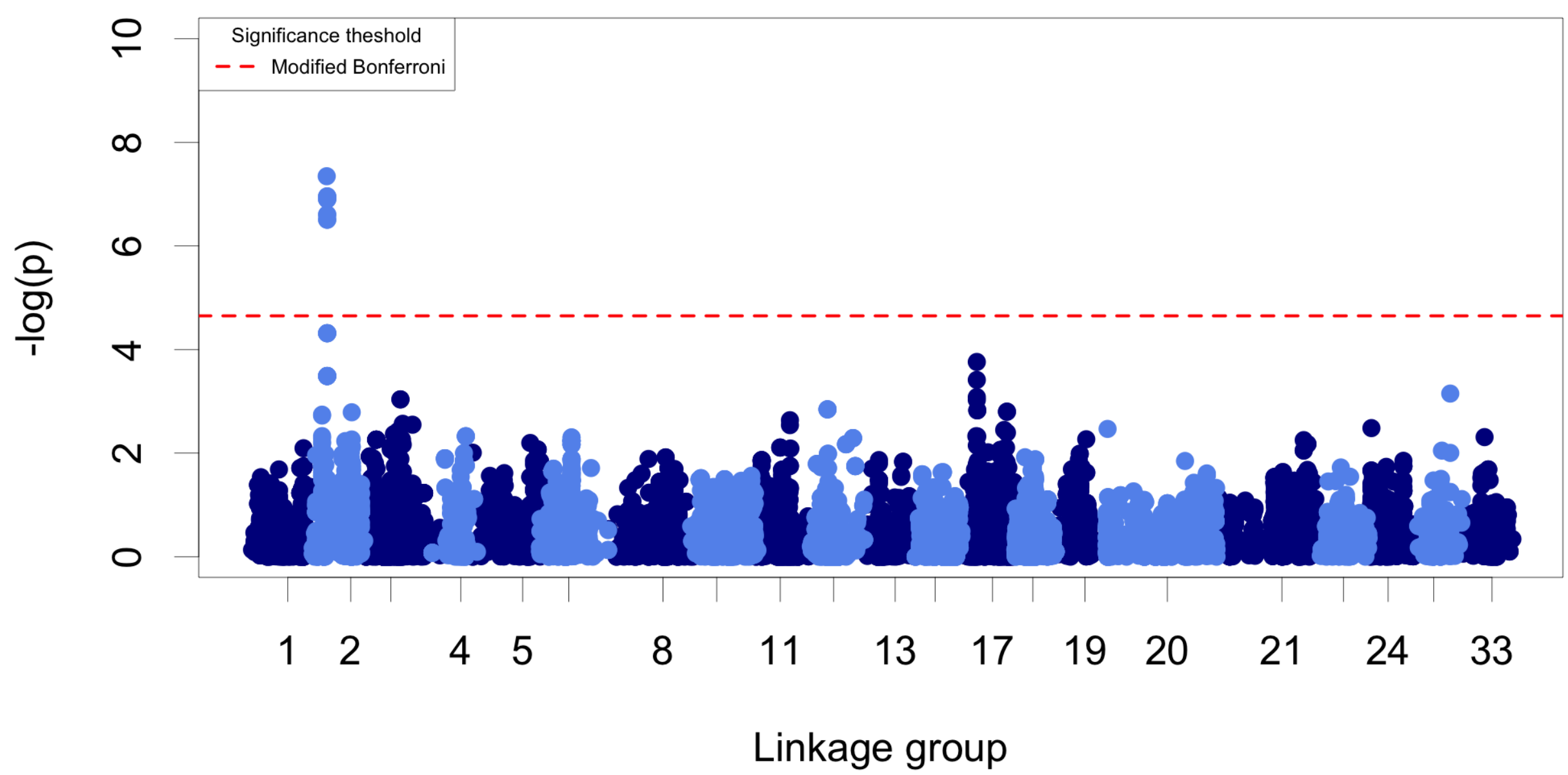

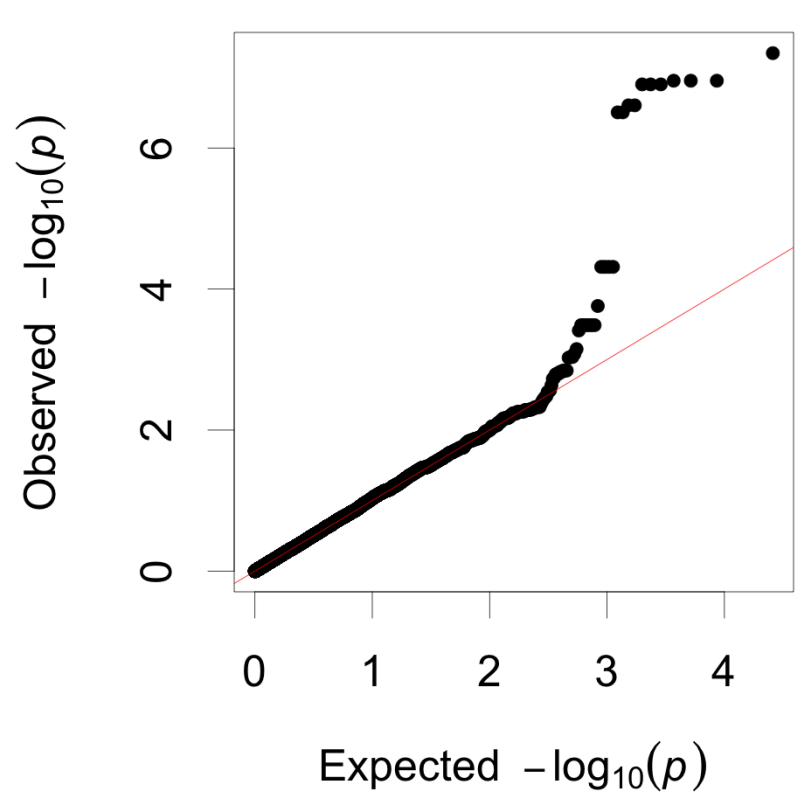

2017 late trial AUDPC

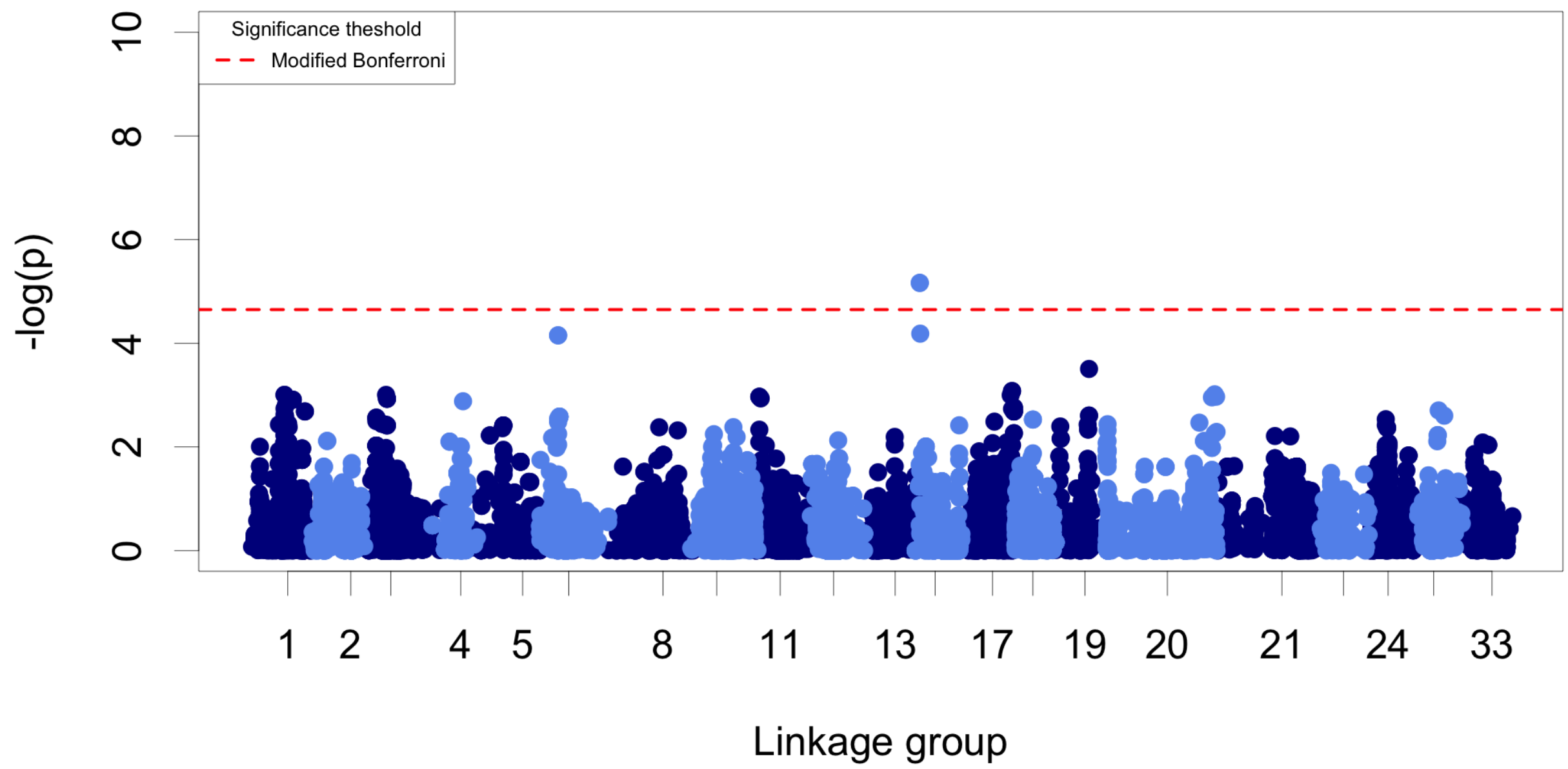

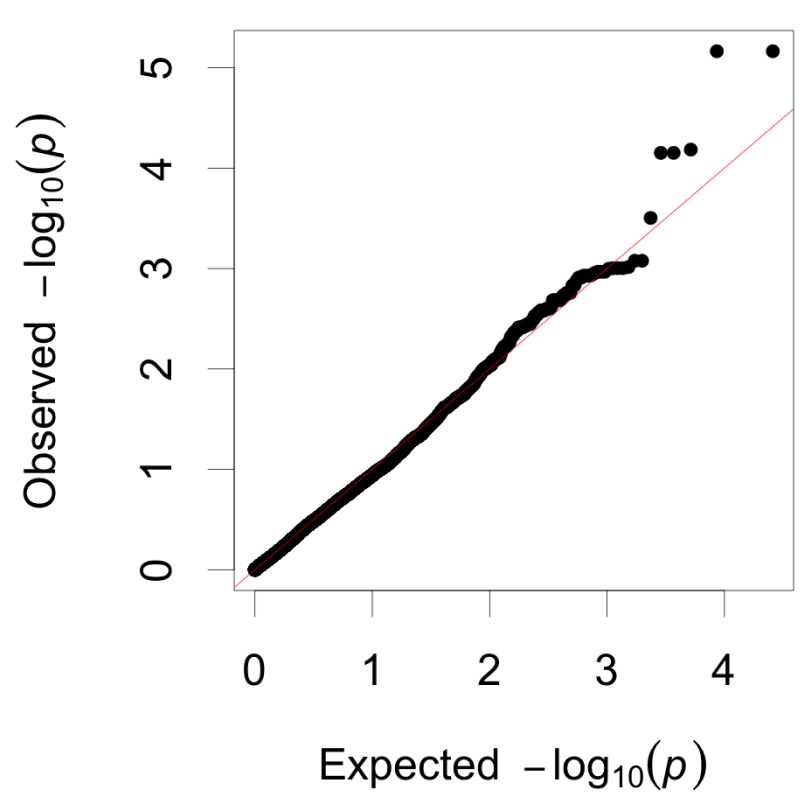

2017 late trial 21-Jun-17 observation

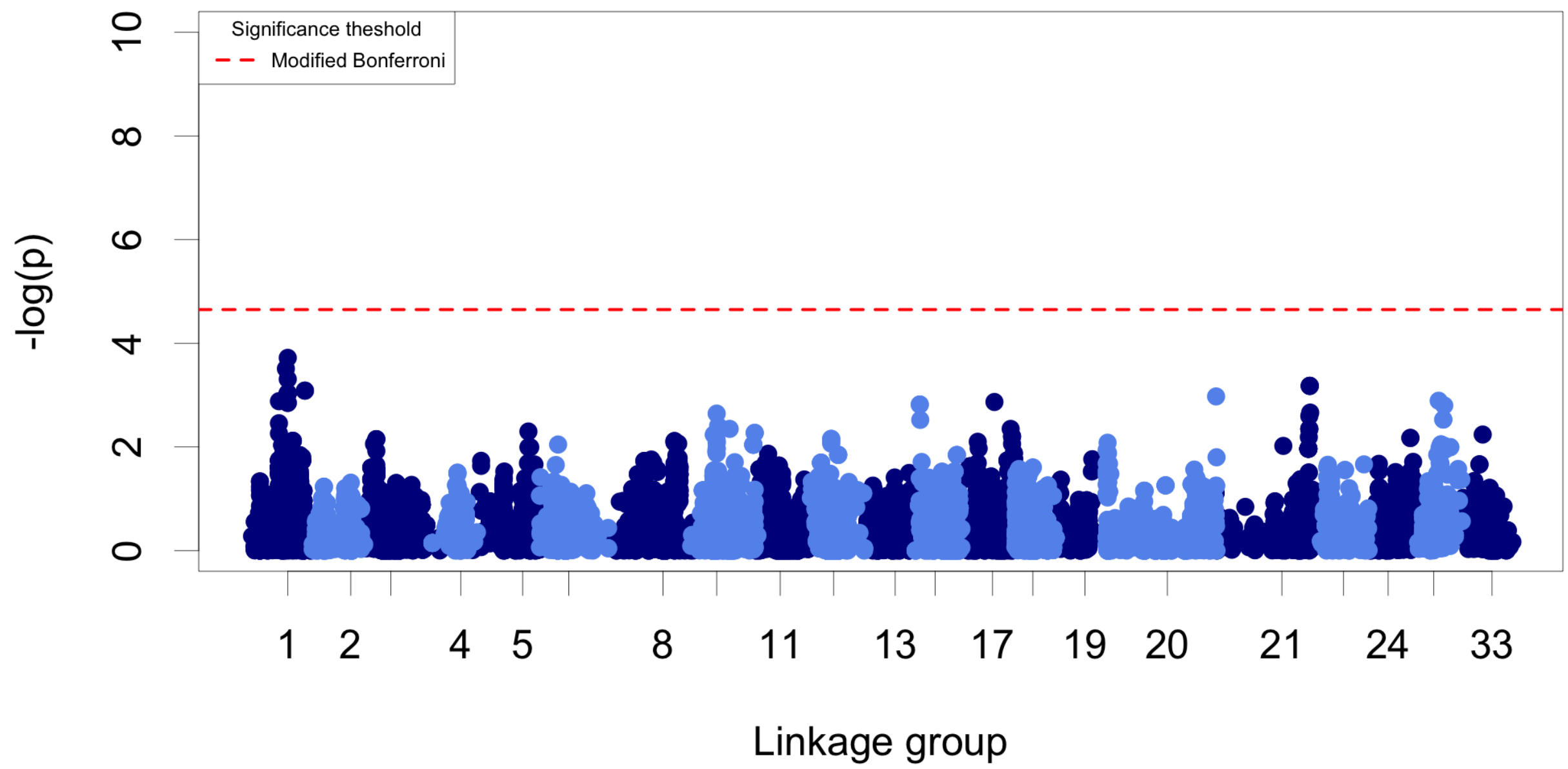

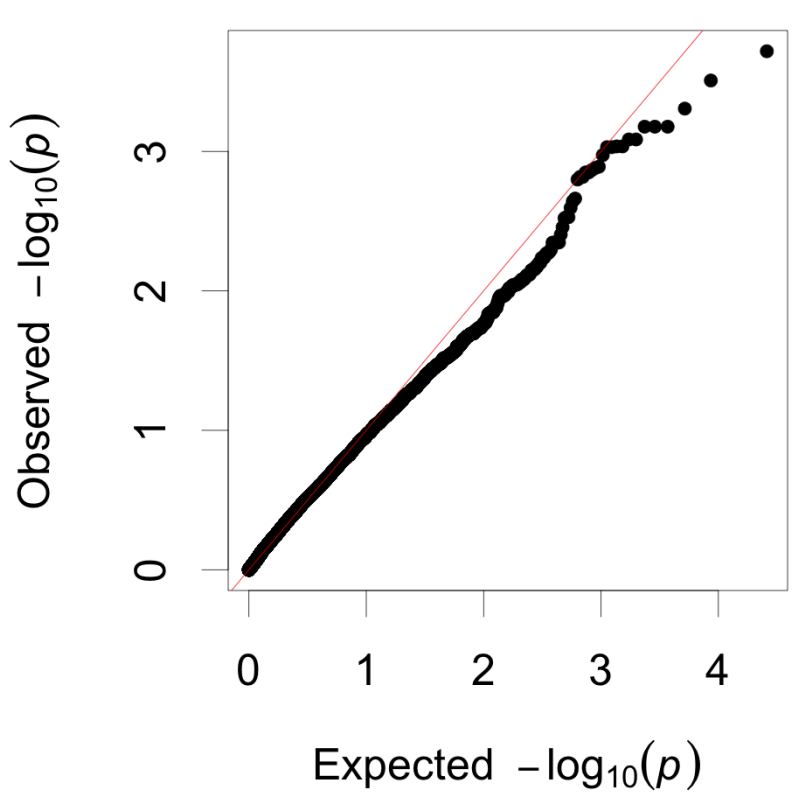

2017 late trial 23-Jun-17 observation

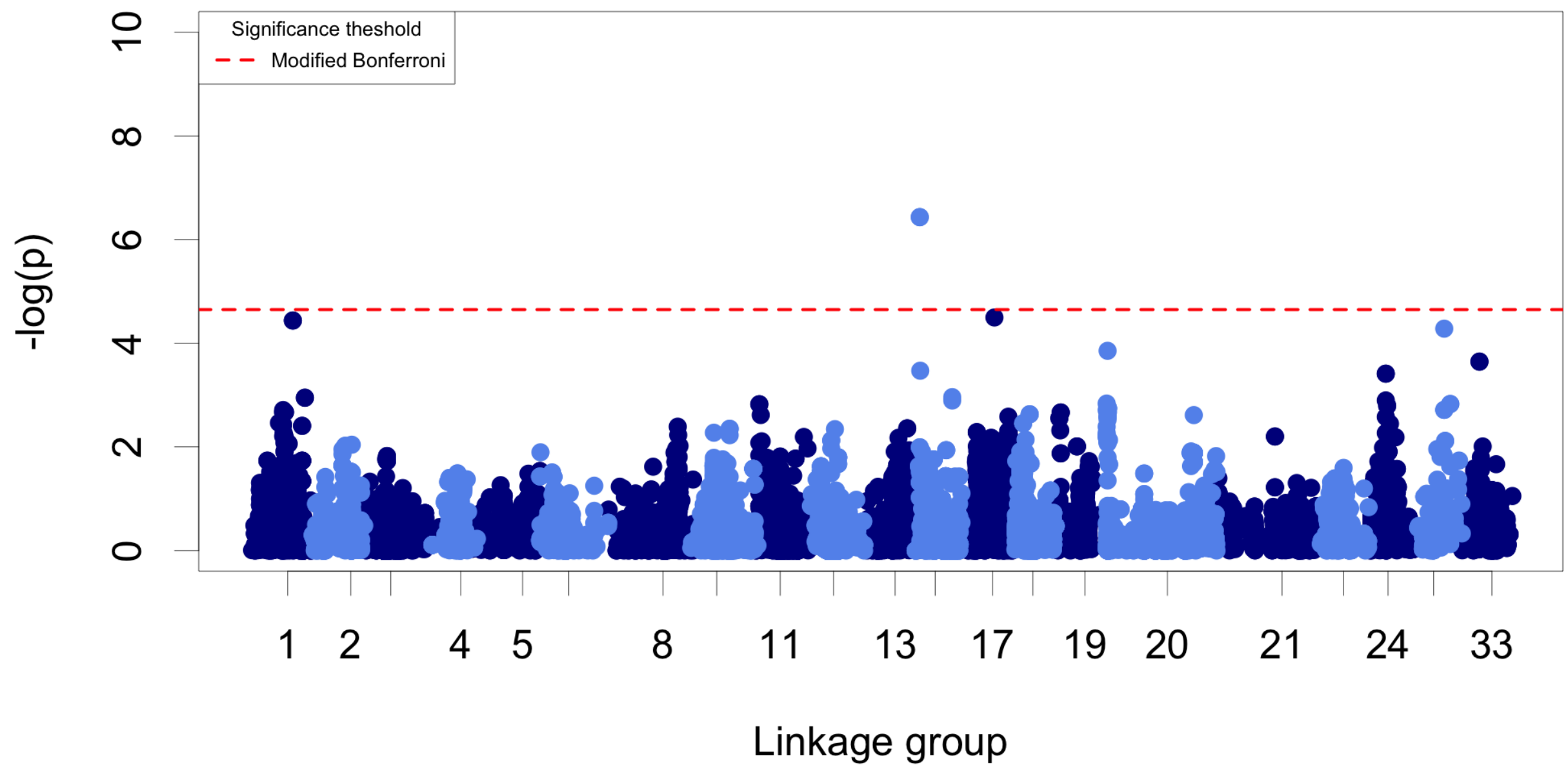

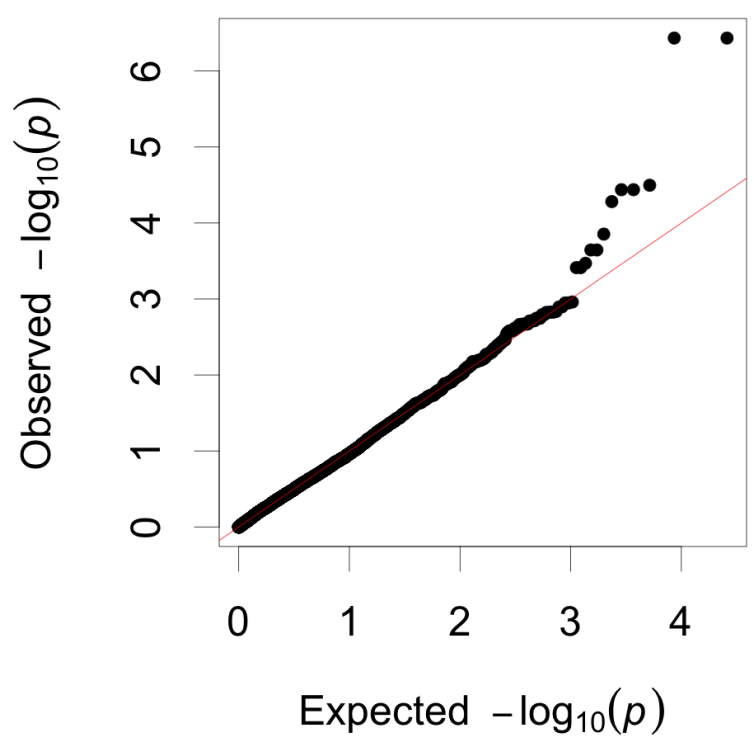

2017 late trial 26-Jun-17 observation

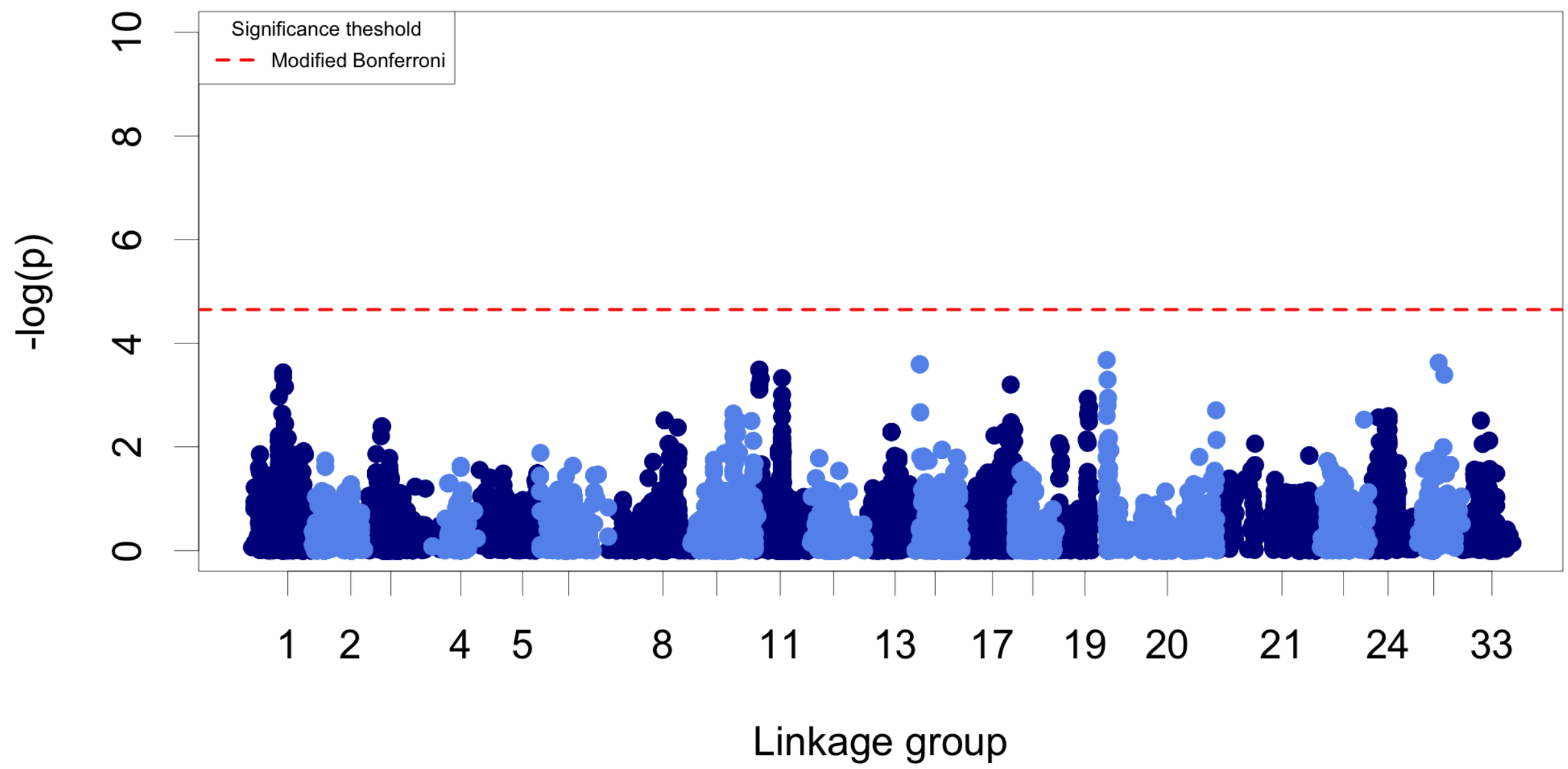

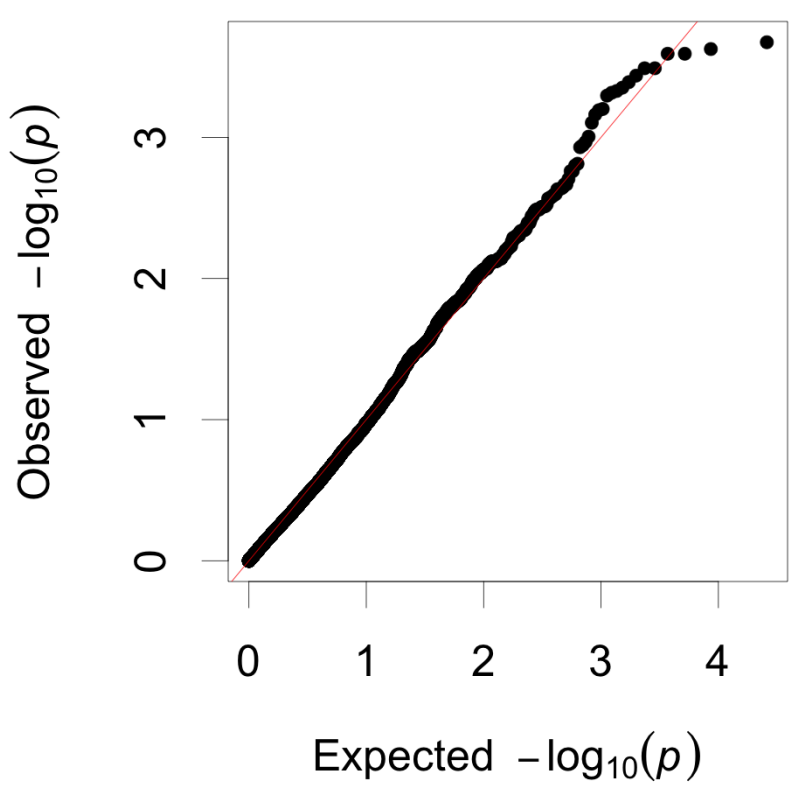

2017 late trial 30-Jun-17 observation

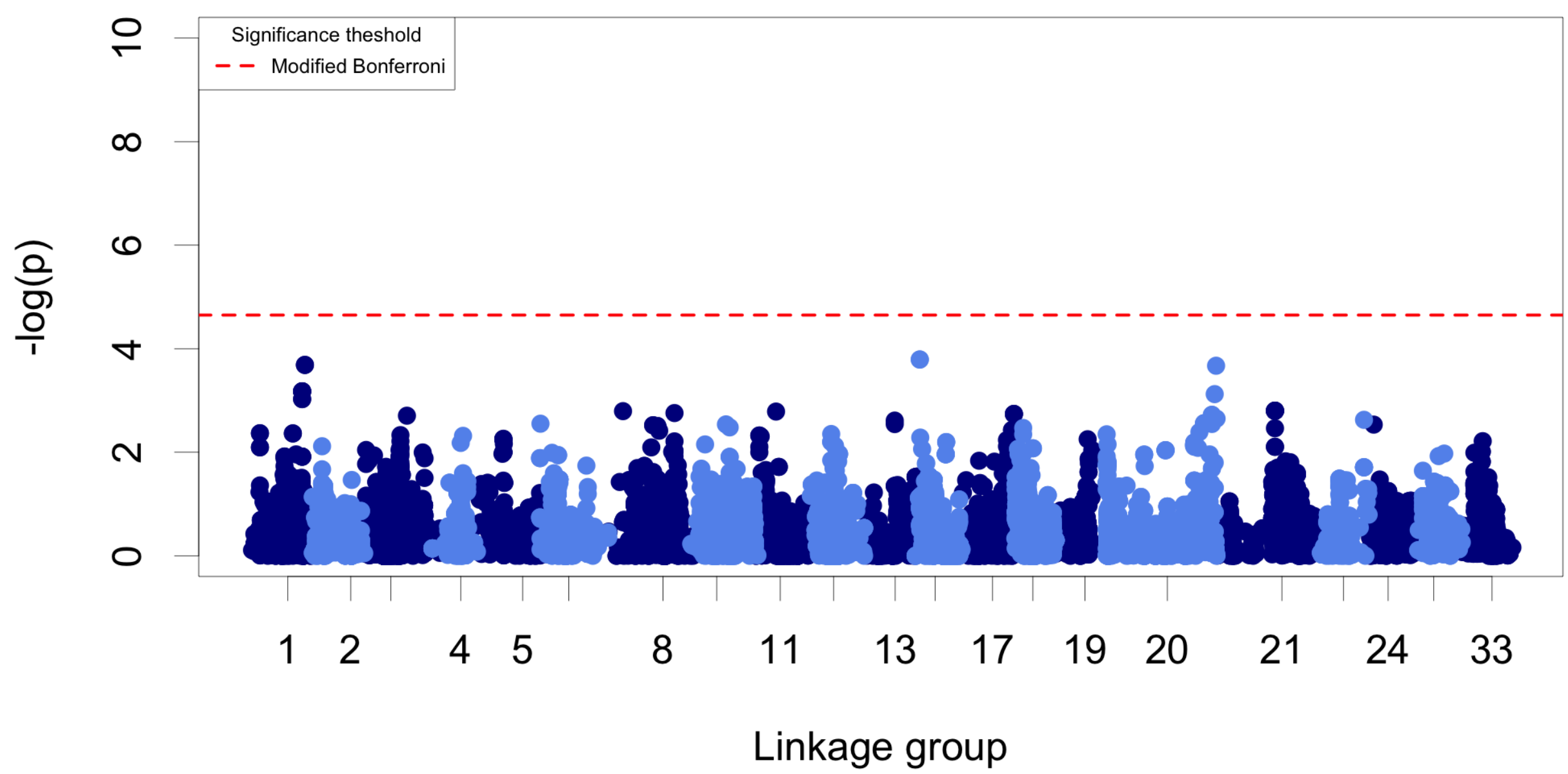

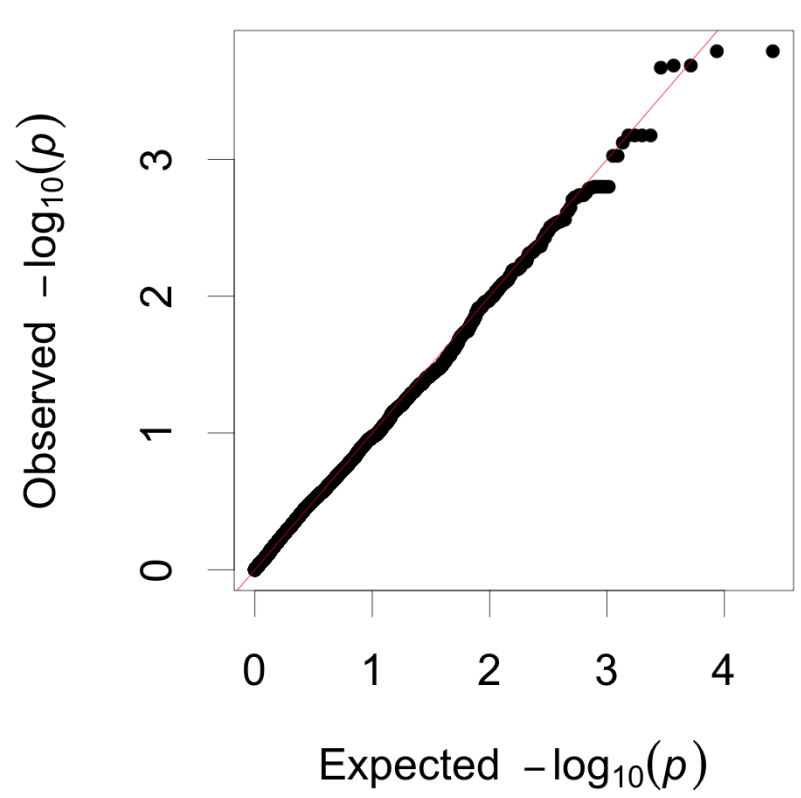

2017 late trial 3-Jul-17 observation

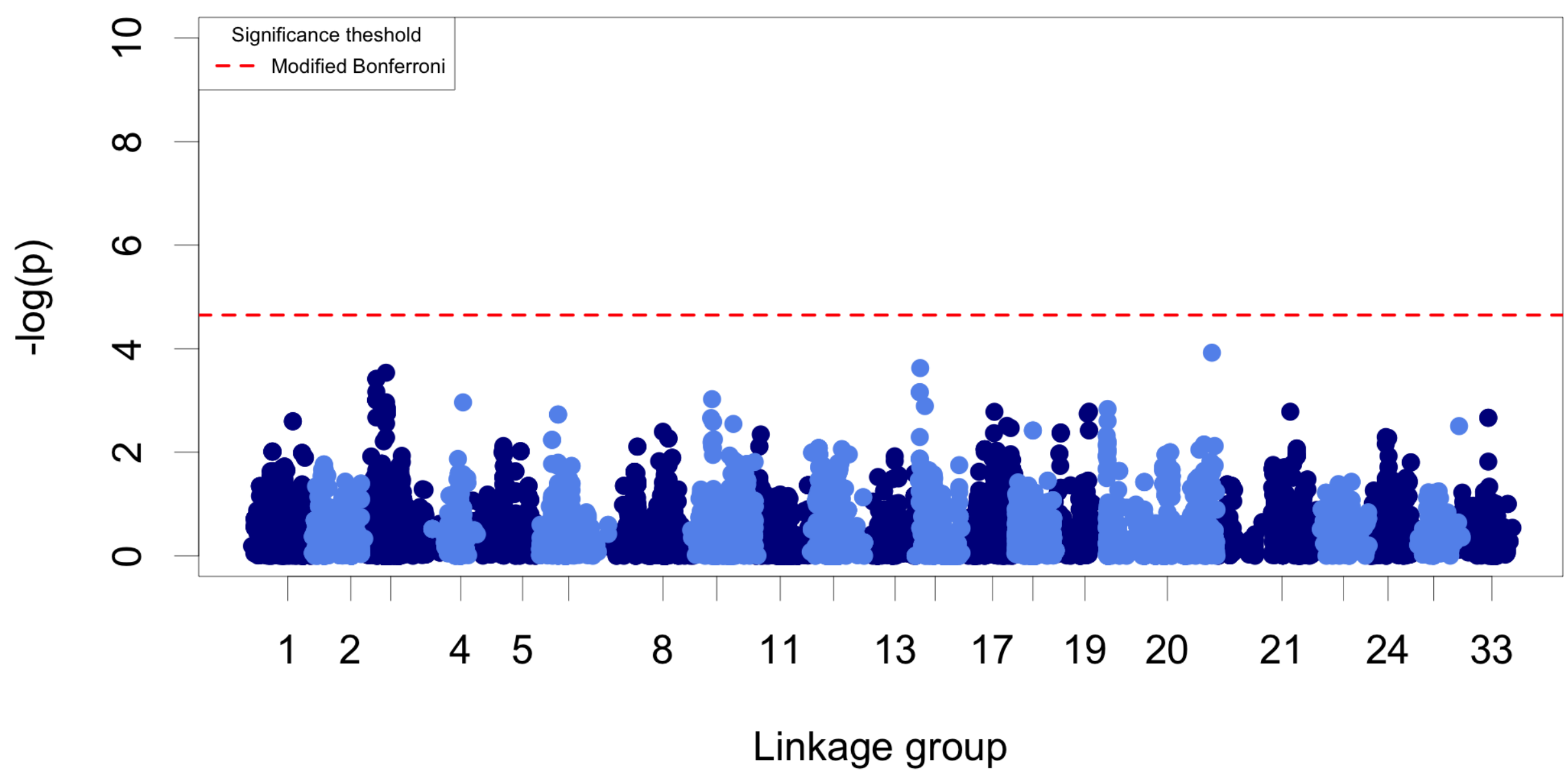

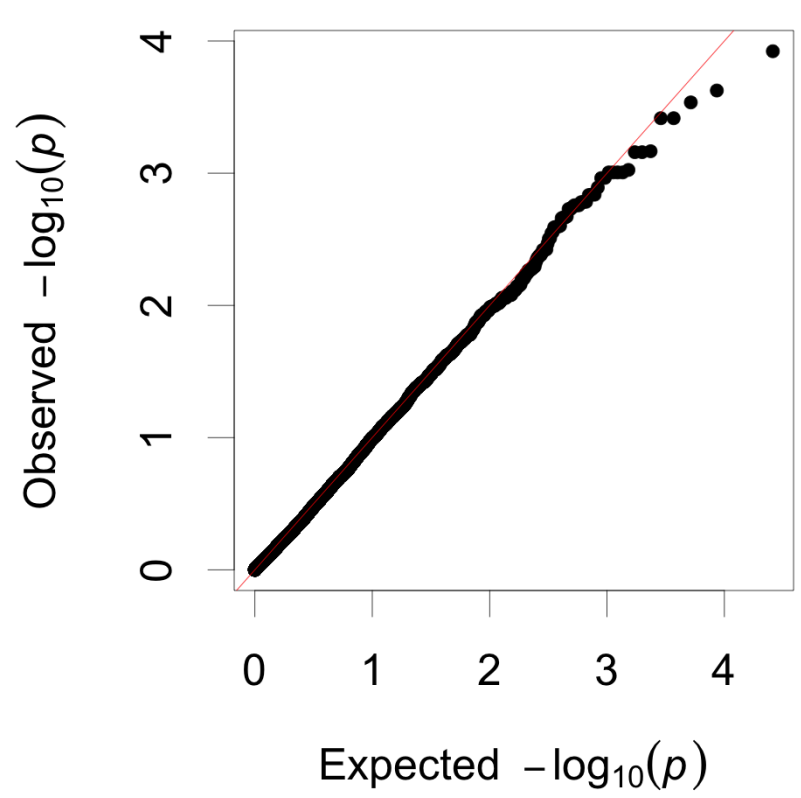

2017 late trial 5-Jul-17 observation

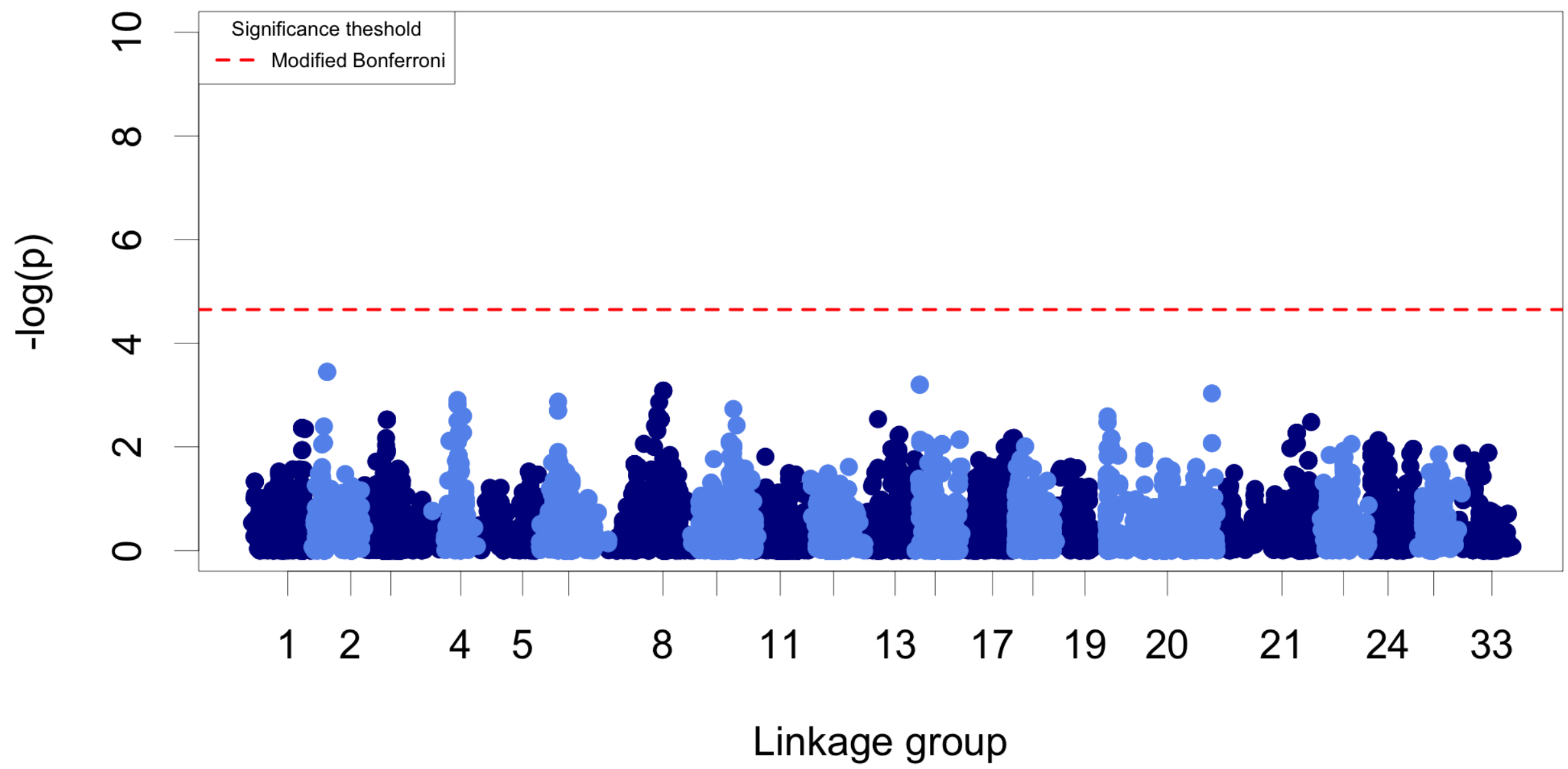

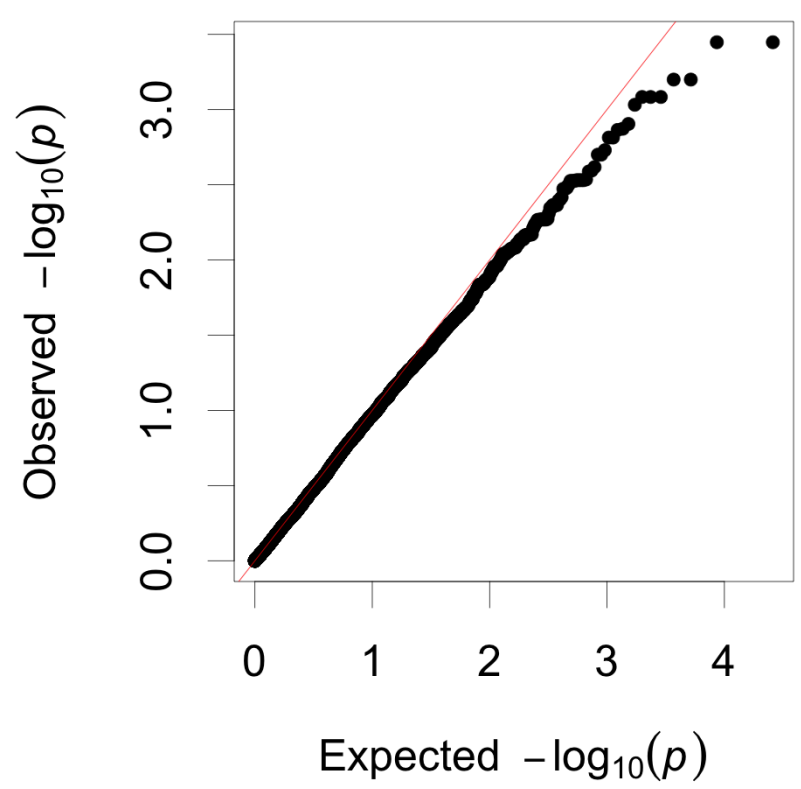

2017 late trial 7-Jul-17 observation

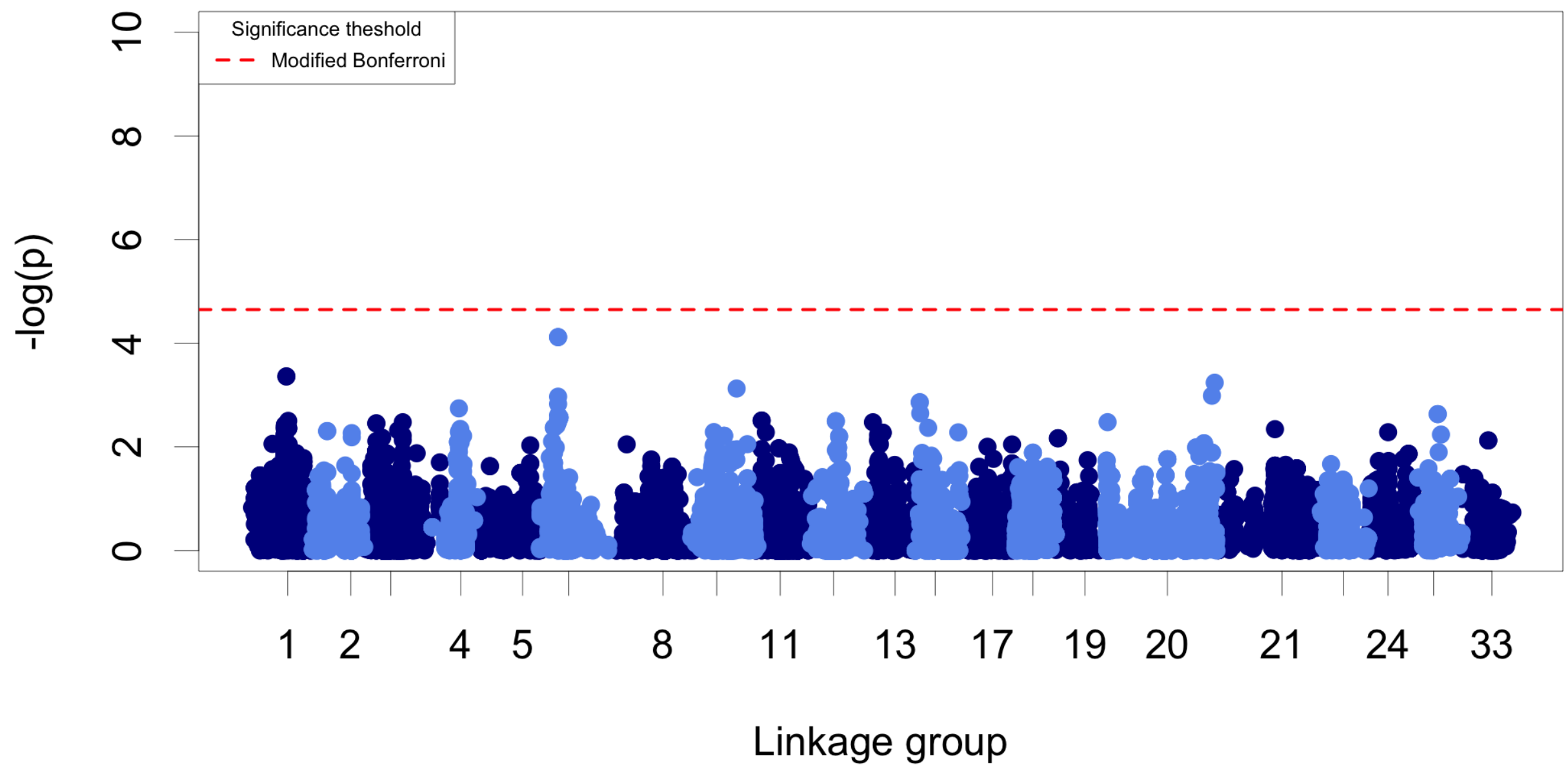

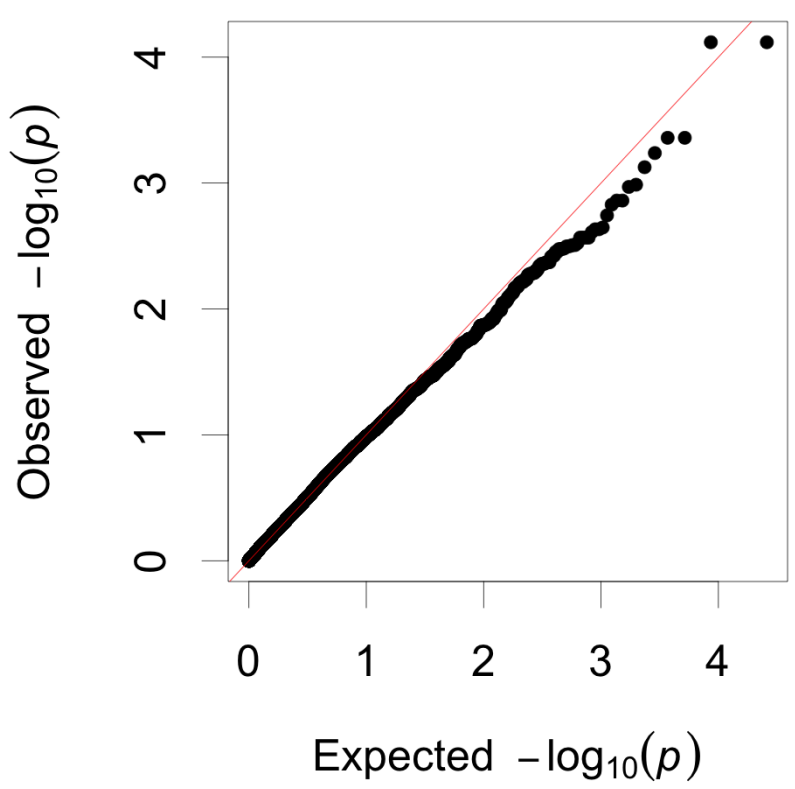

2017 late trial 10-Jul-17 observation

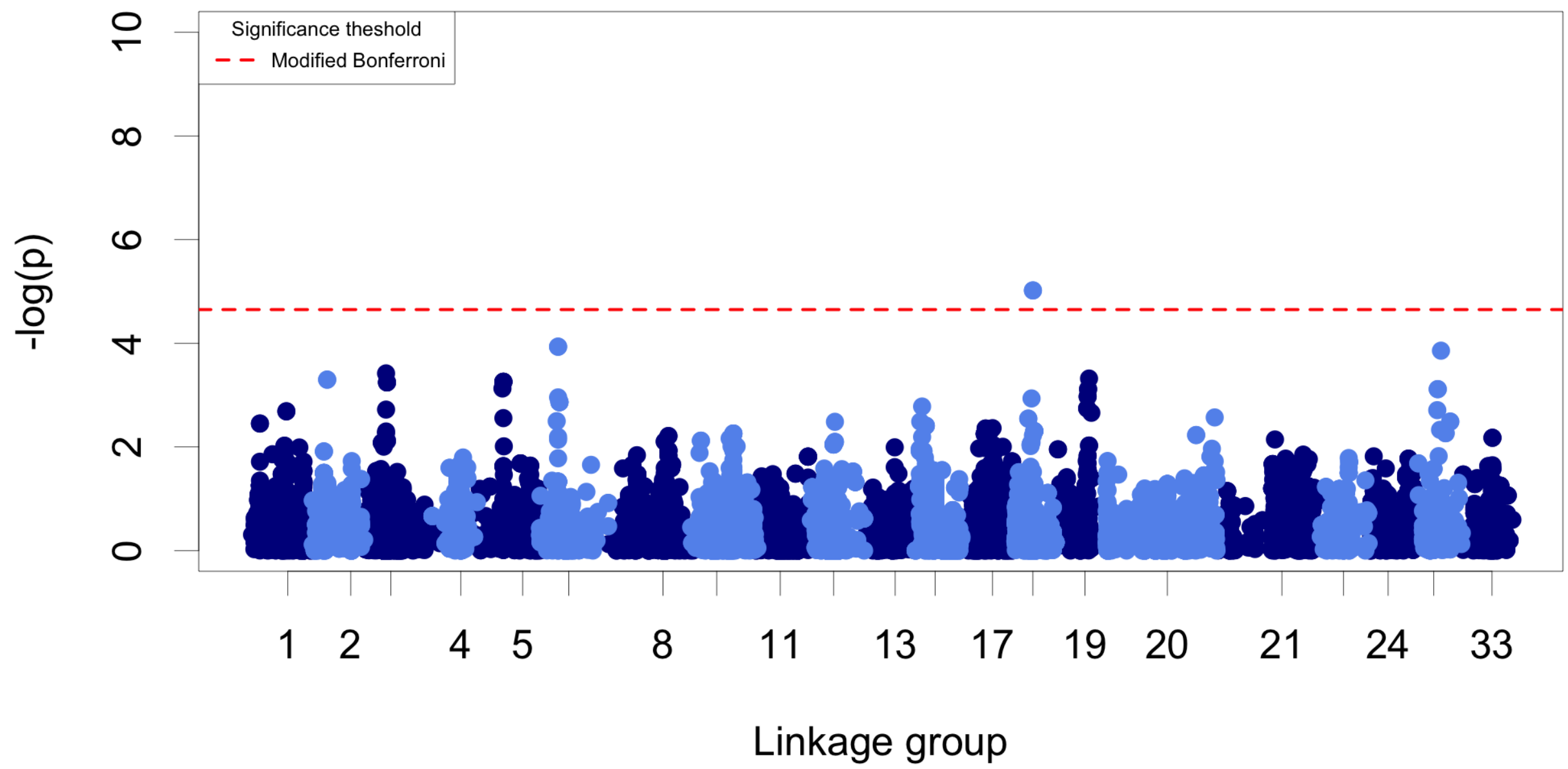

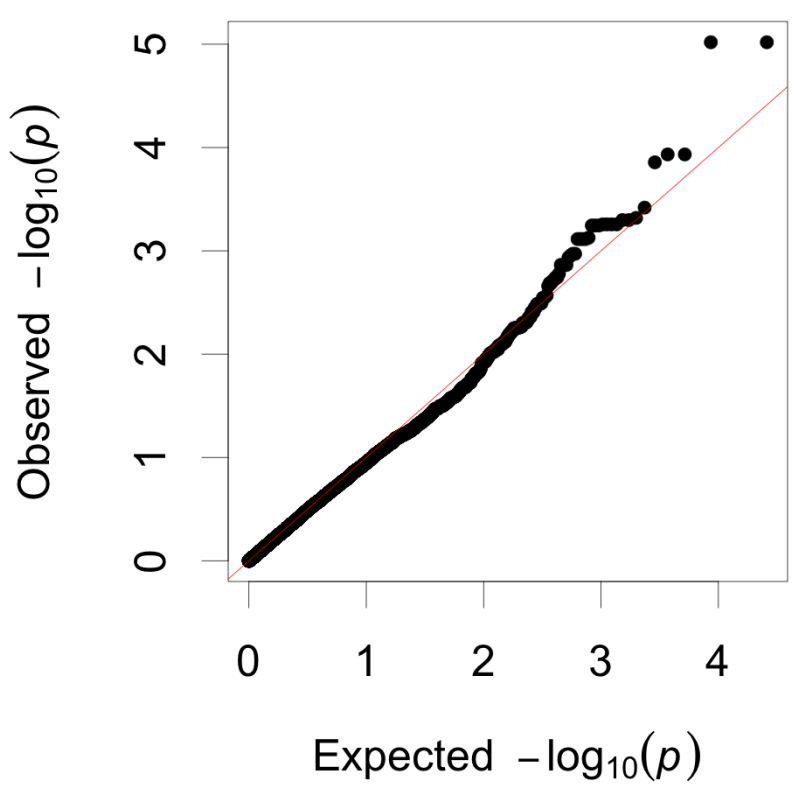

2017 late trial heading date

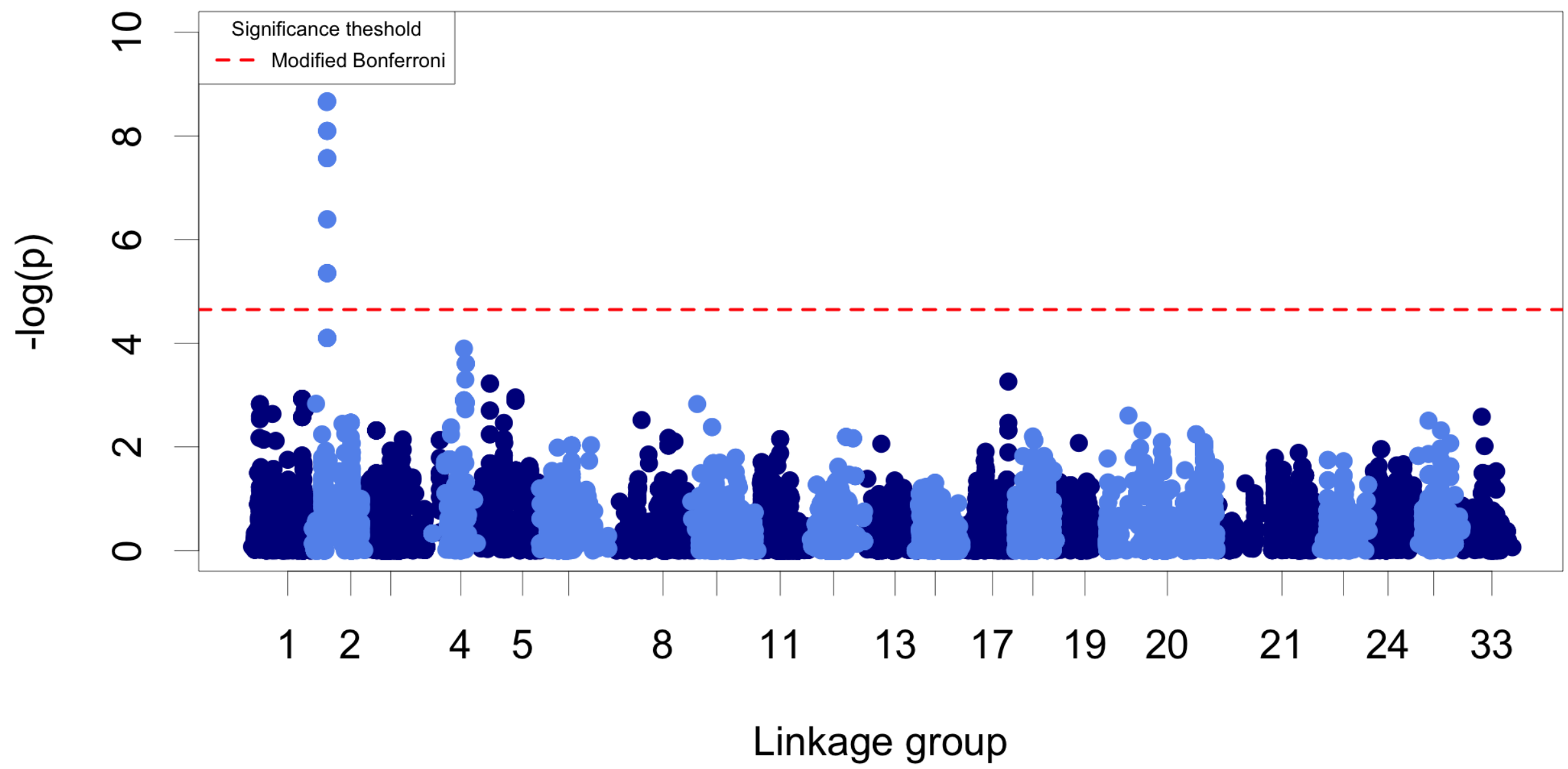

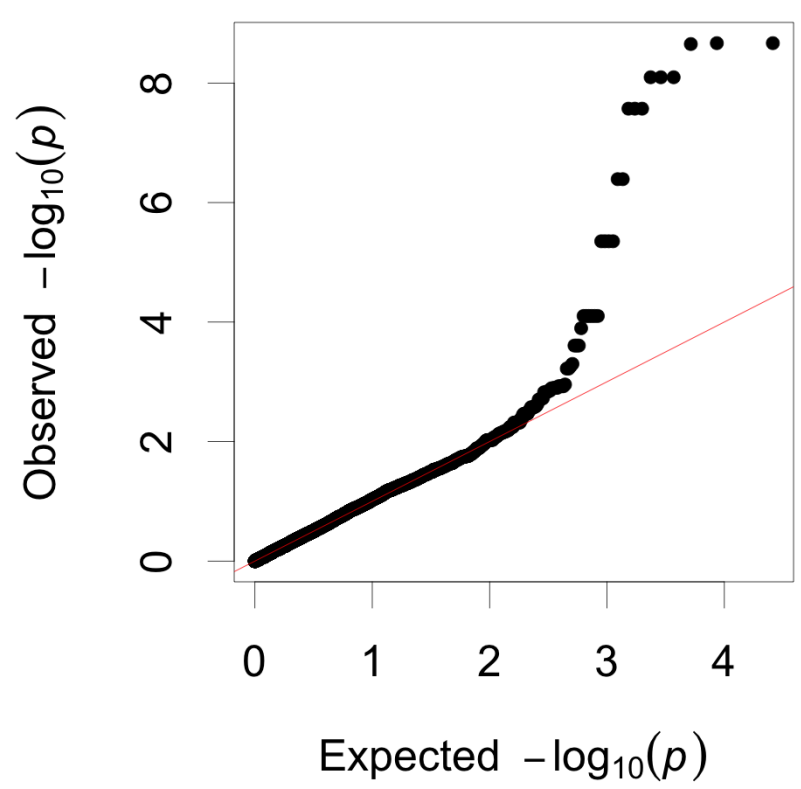

2018 trial AUDPC

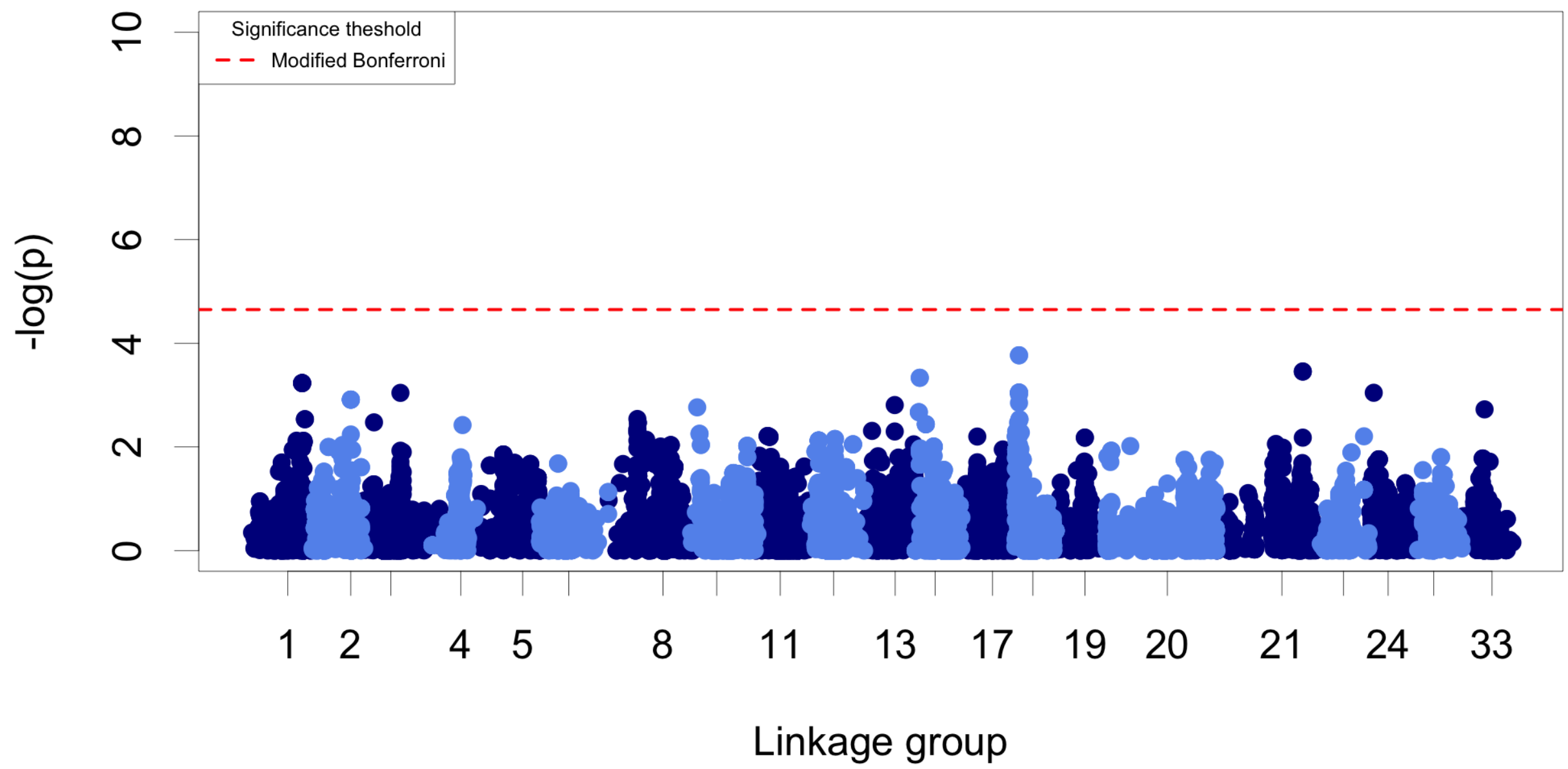

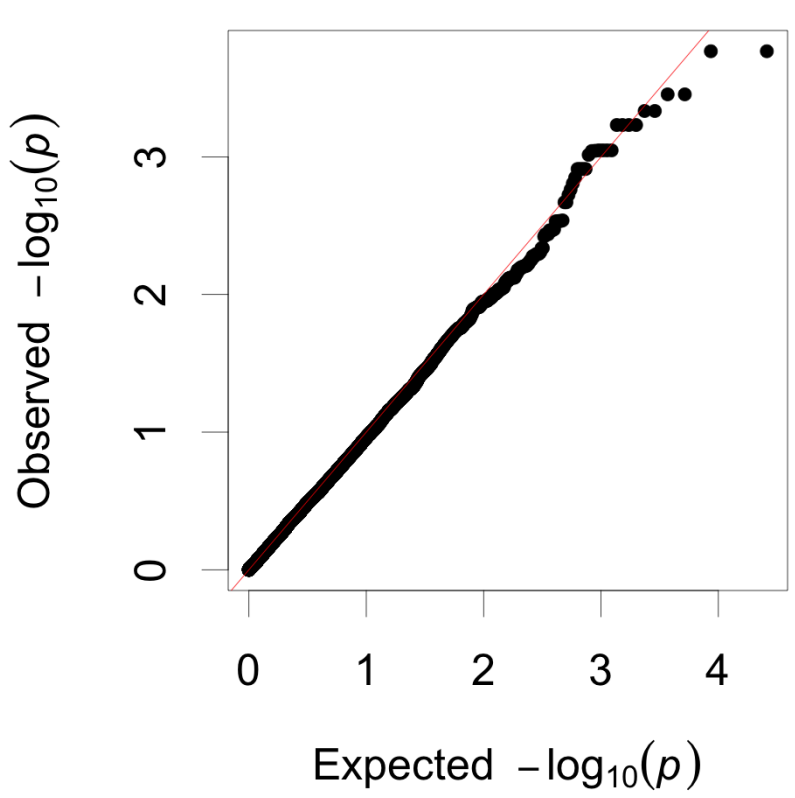

2018 trial 11-Jun-18 observation

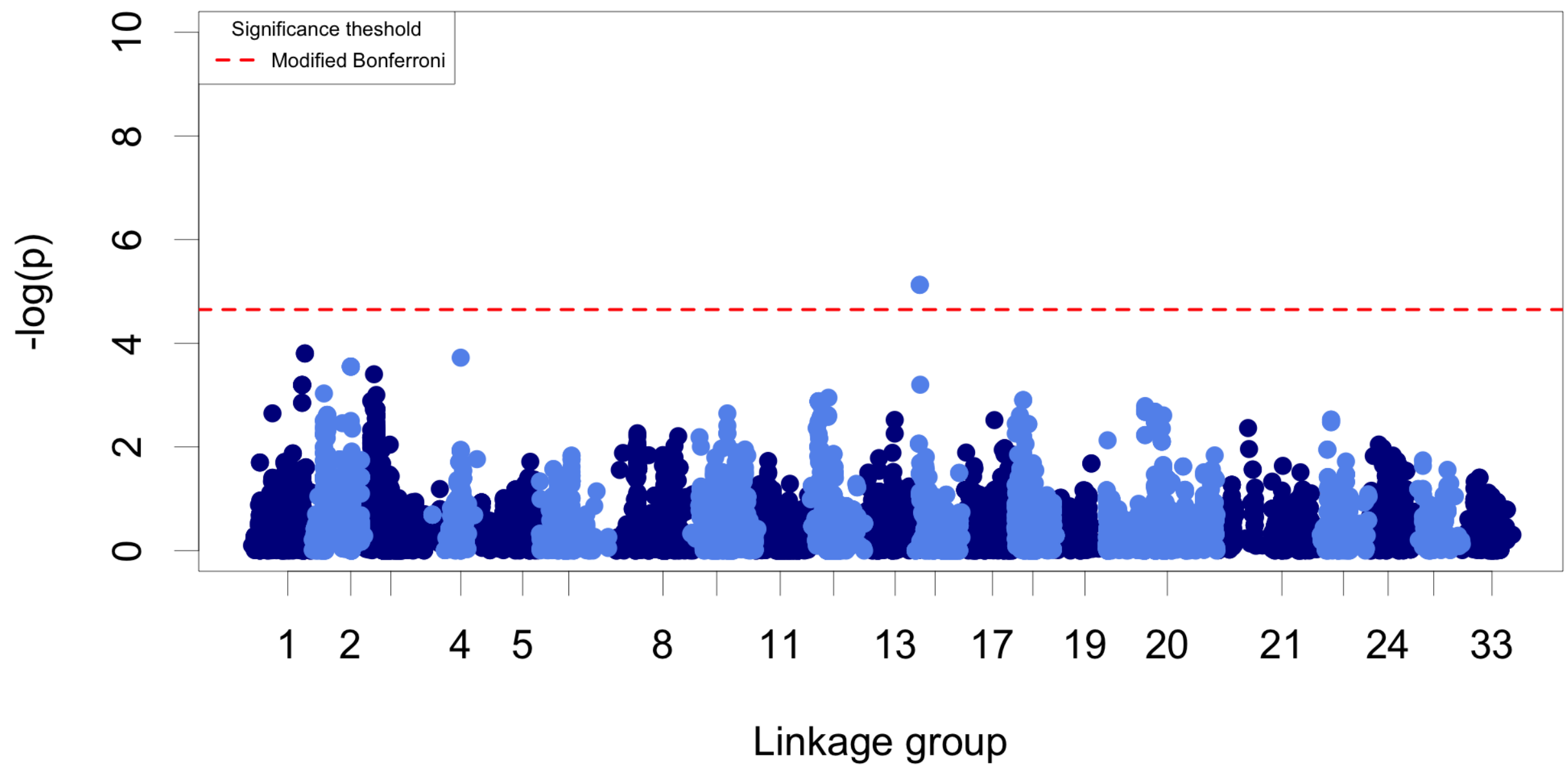

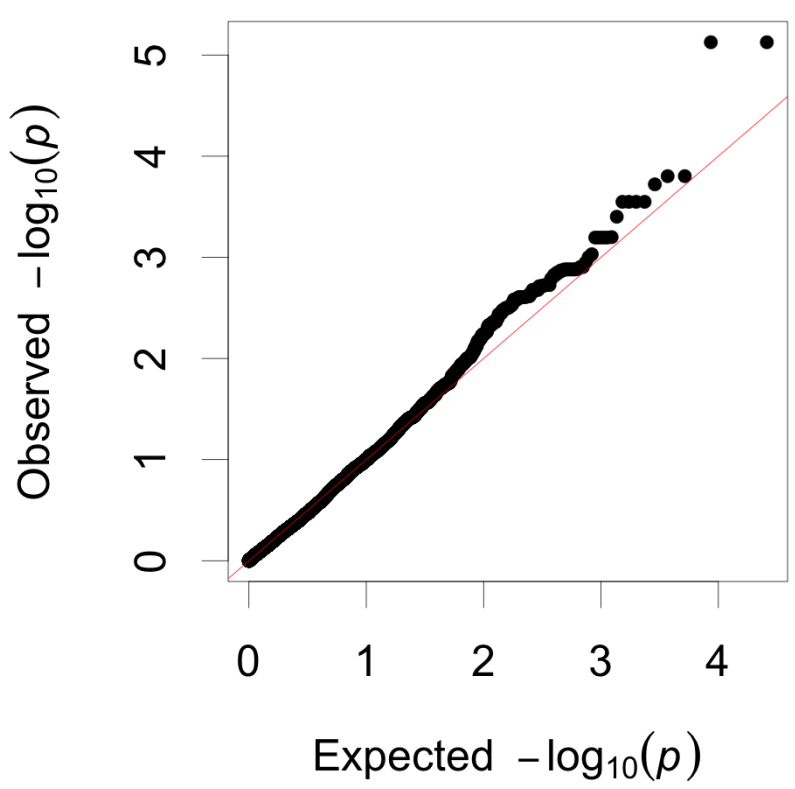

2018 trial 13-Jun-18 observation

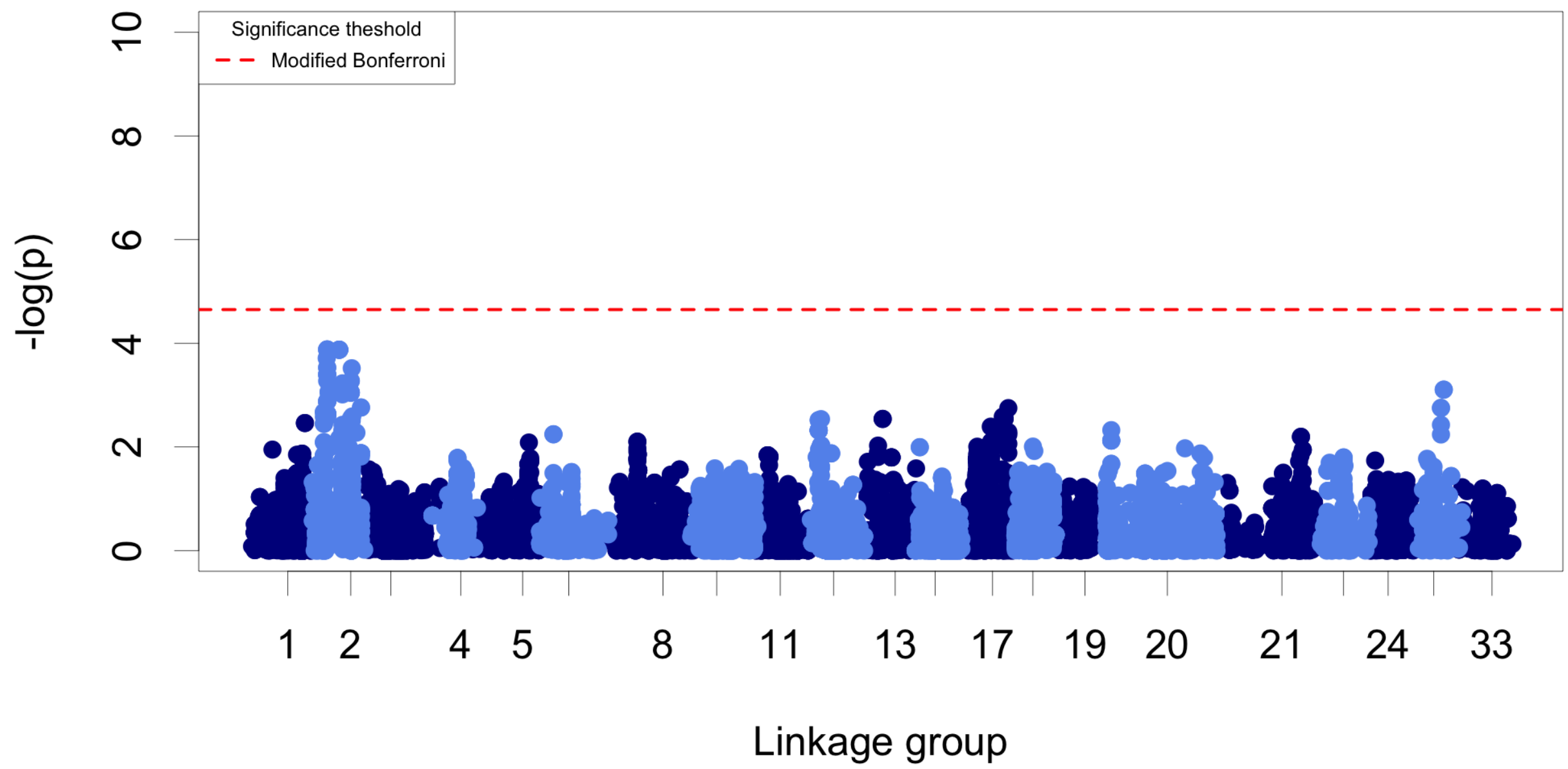

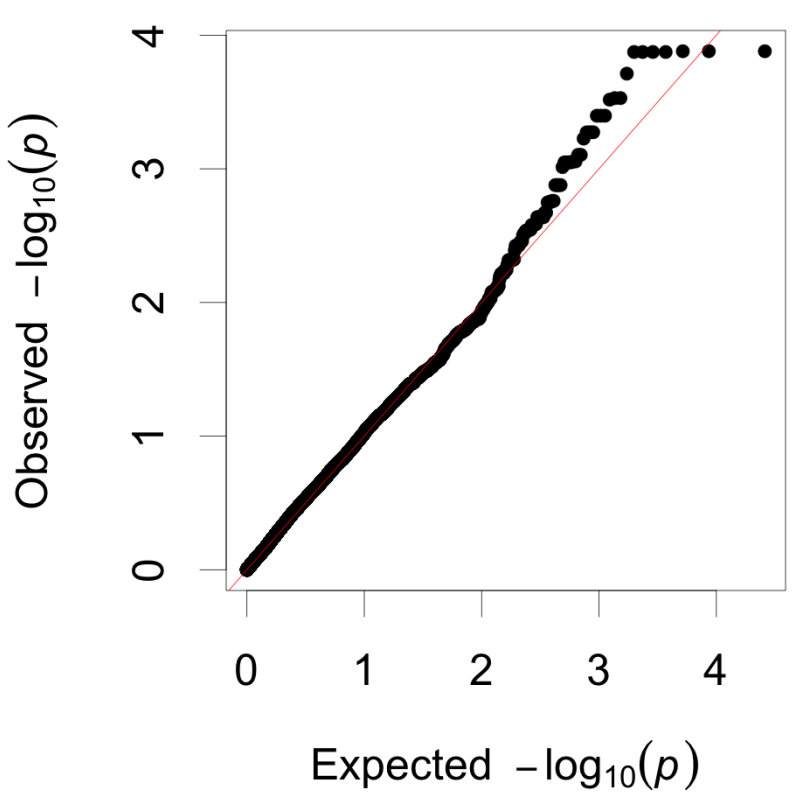

2018 trial 15-Jun-18 observation

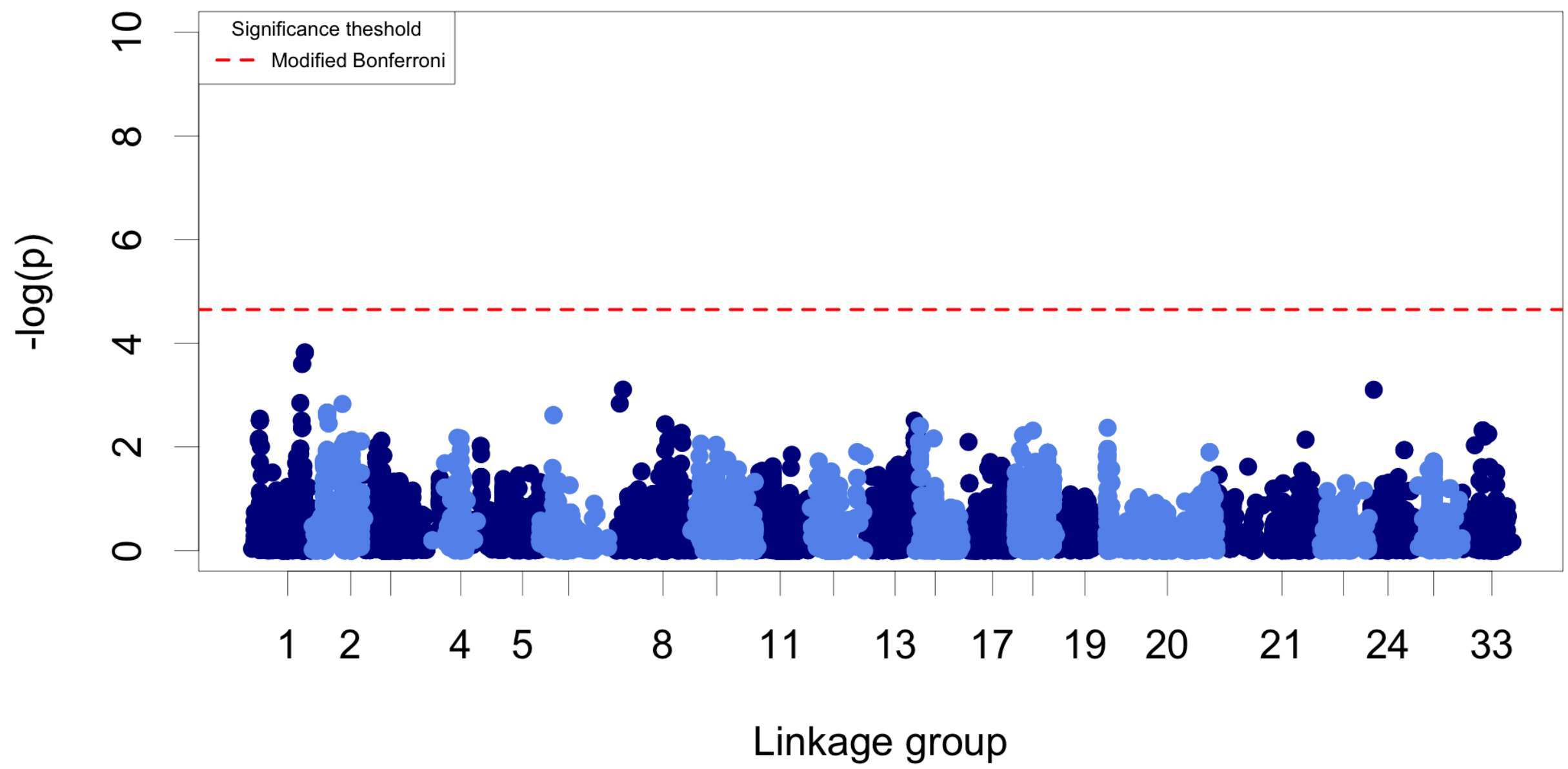

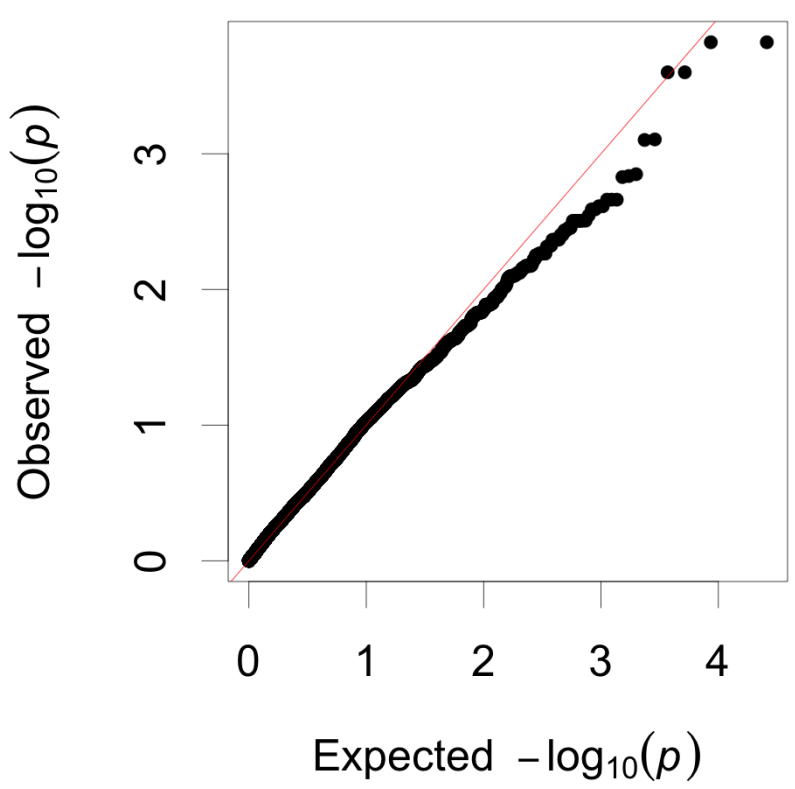

2018 trial 20-Jun-18 observation

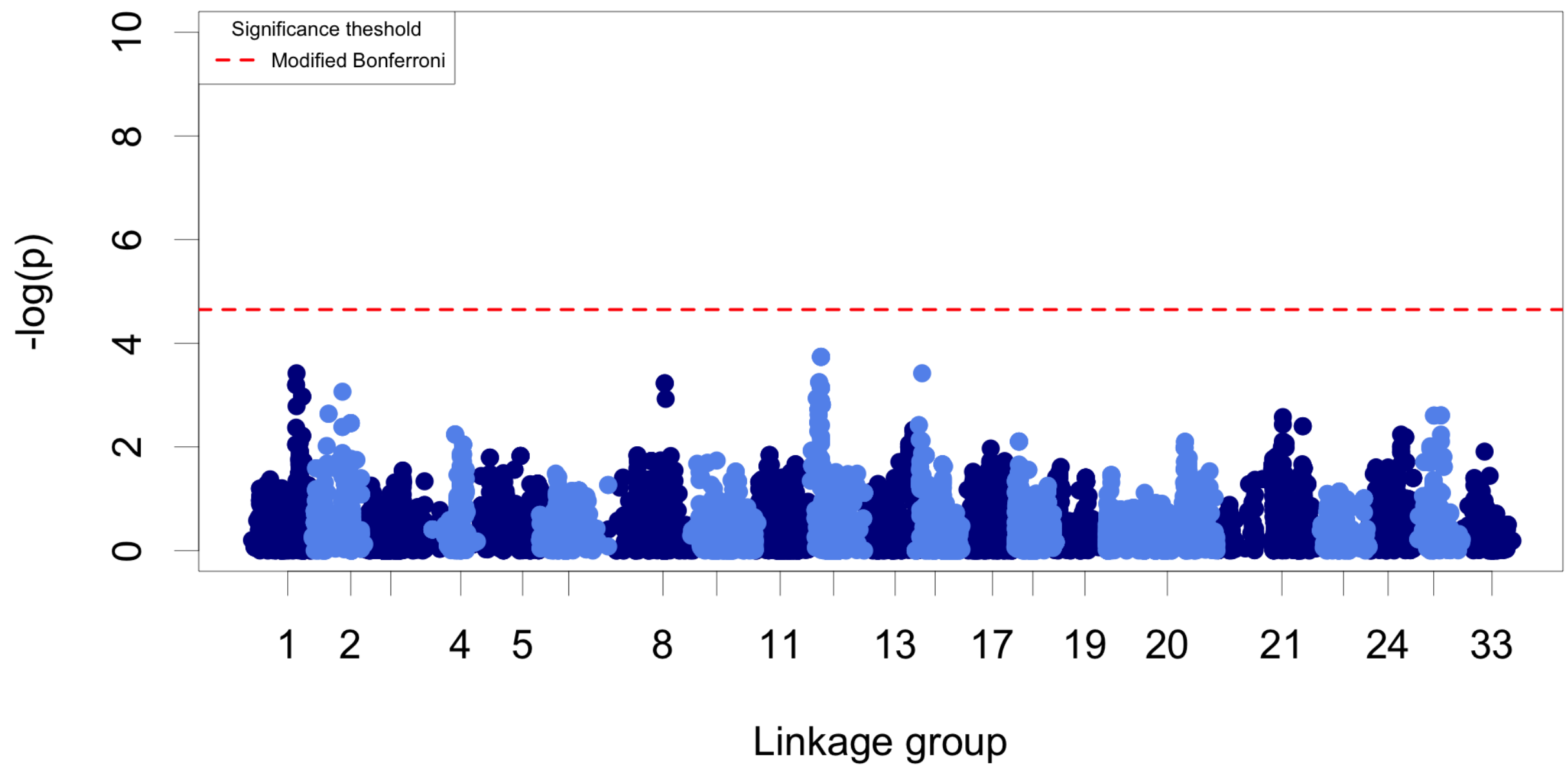

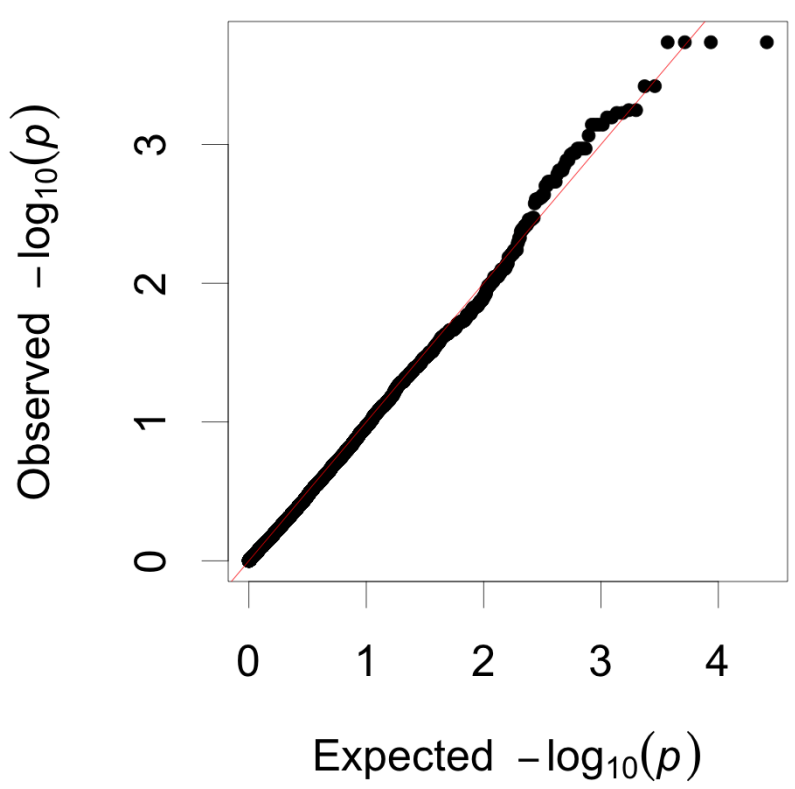

2018 trial 22-Jun-18 observation

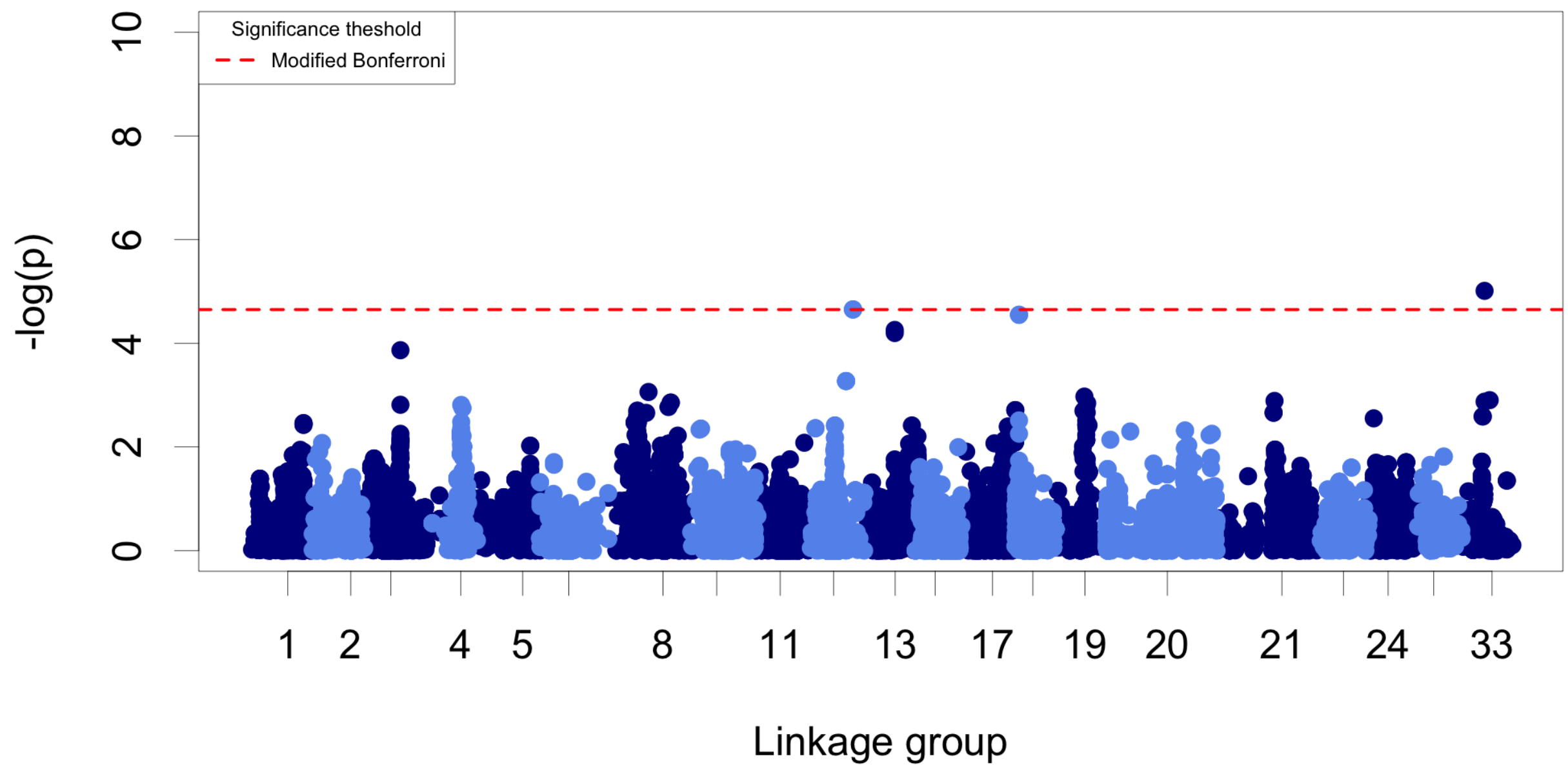

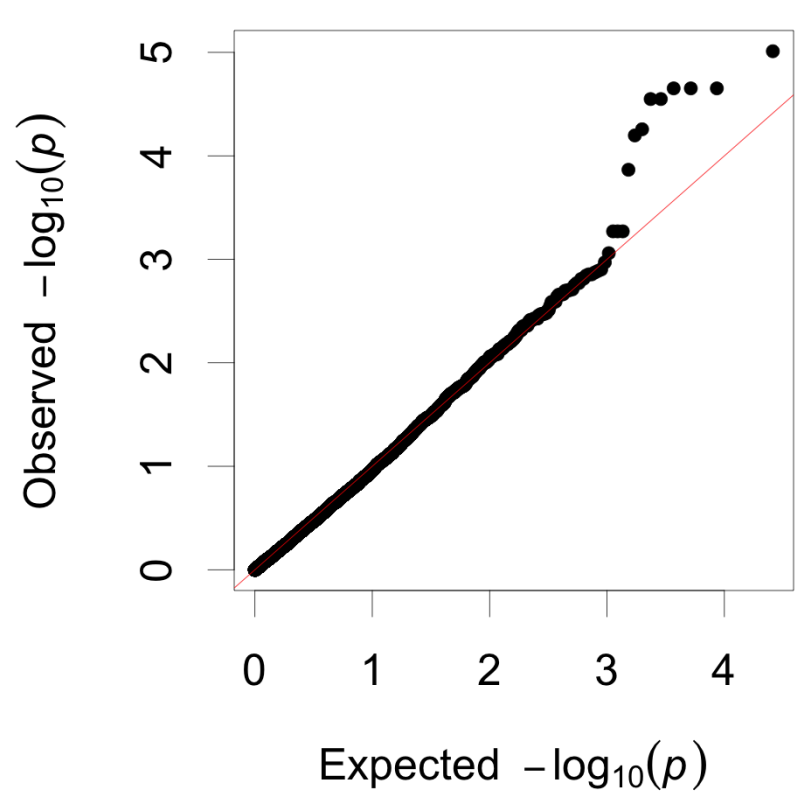

2018 trial 25-Jun-18 observation

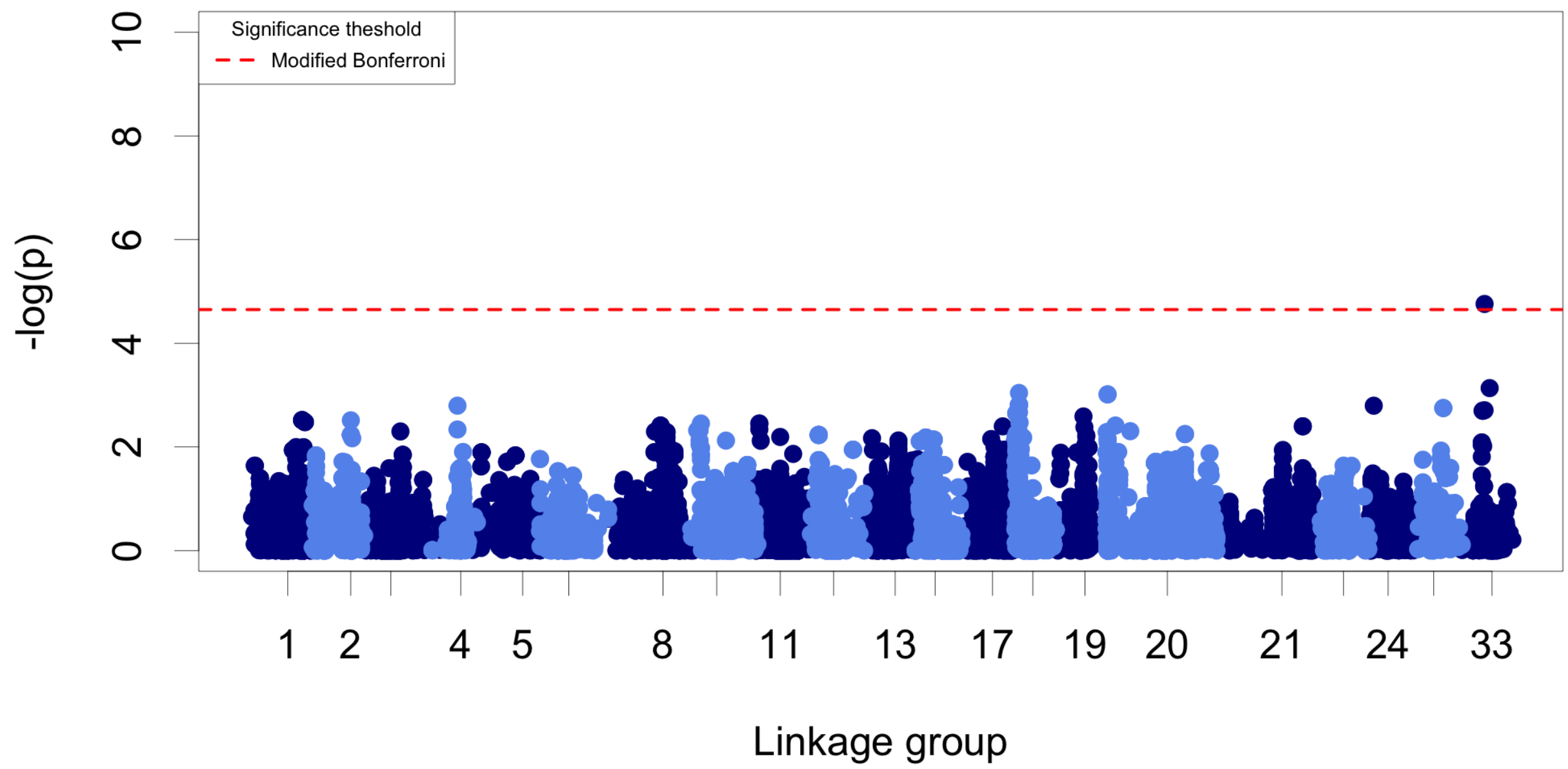

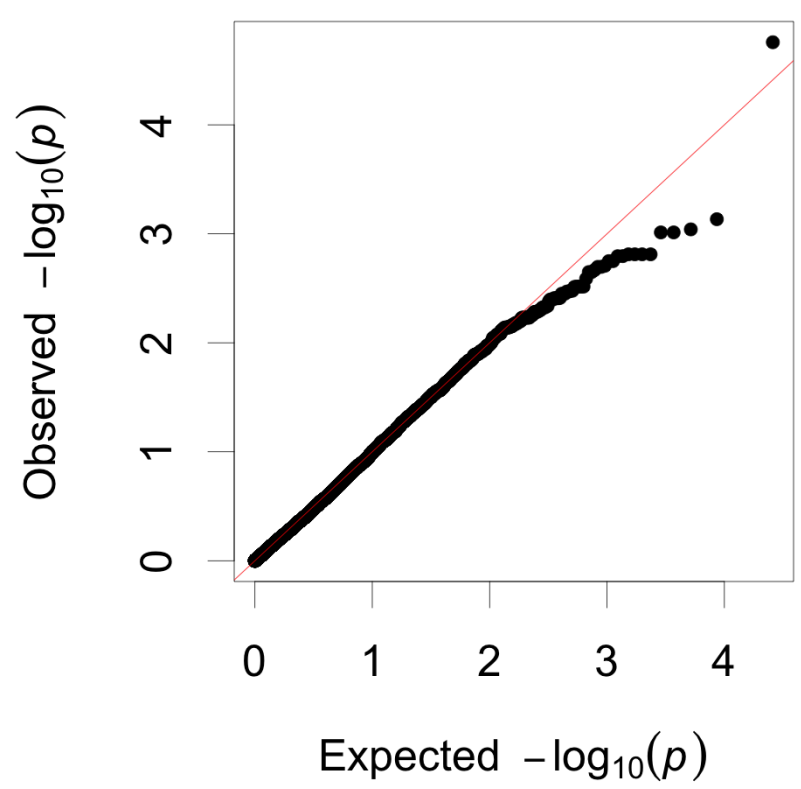

2018 trial 29-Jun-18 observation

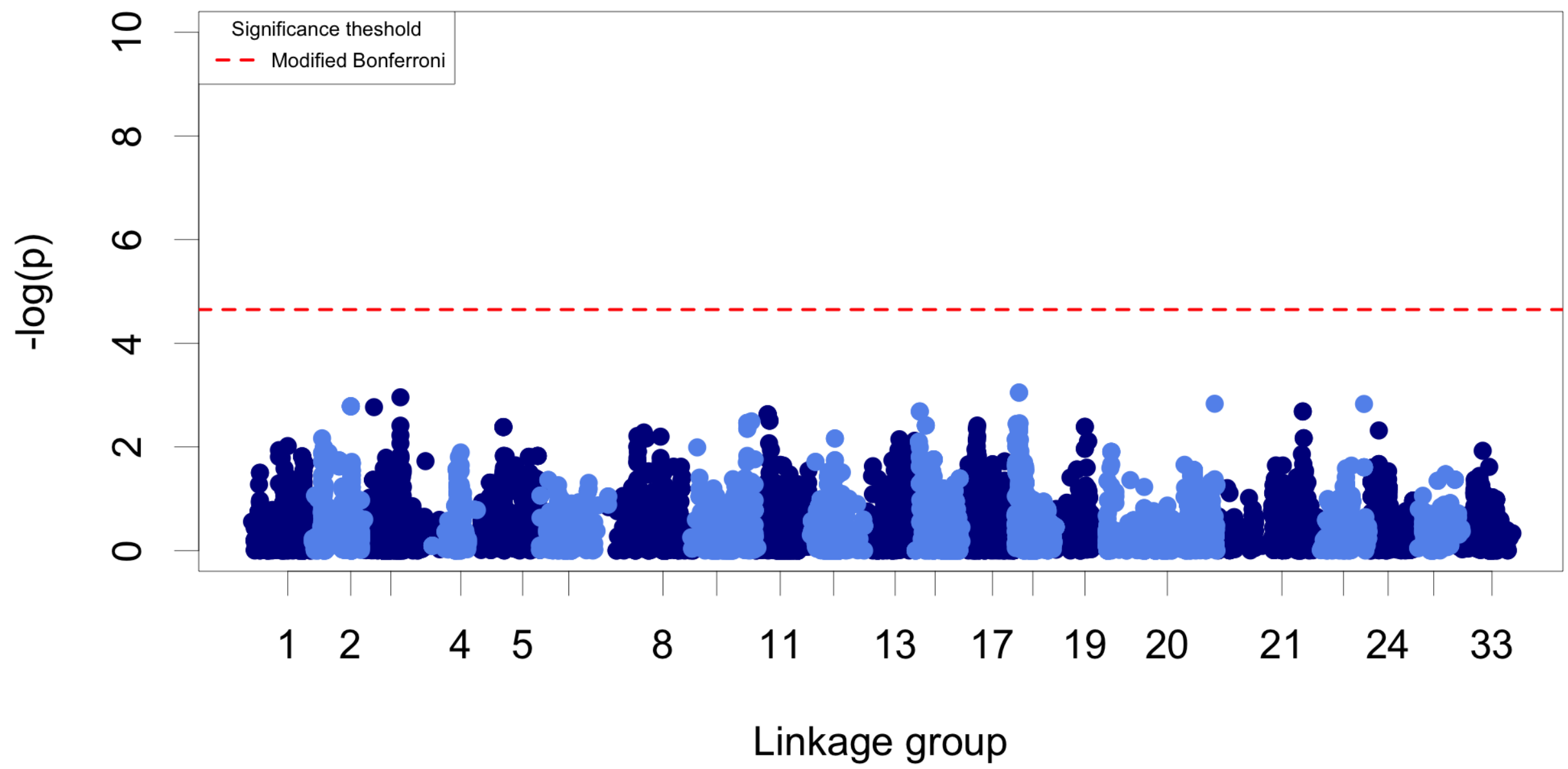

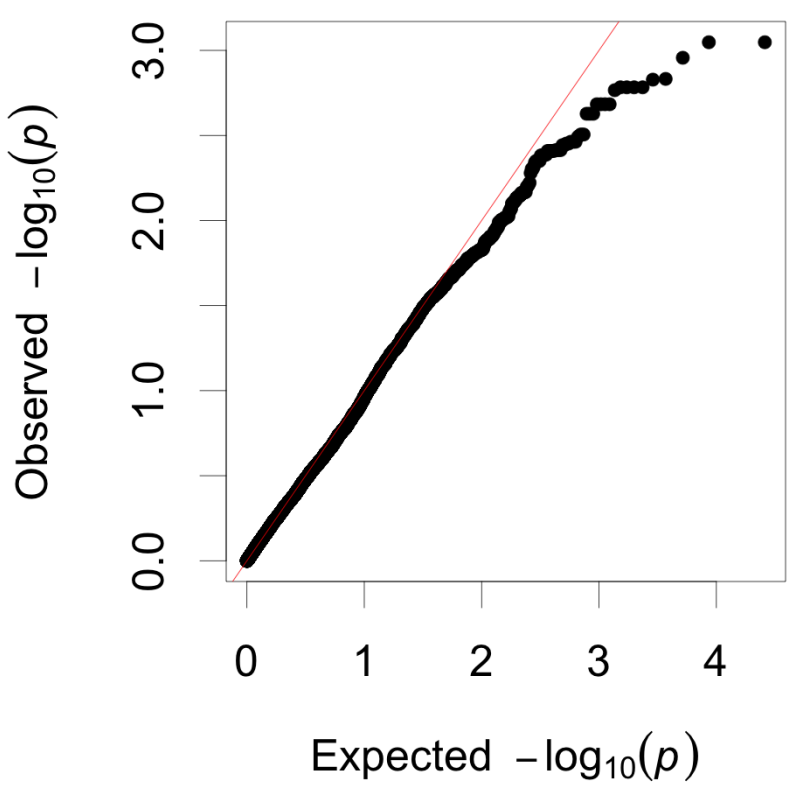

Multi-environmental AUDPC

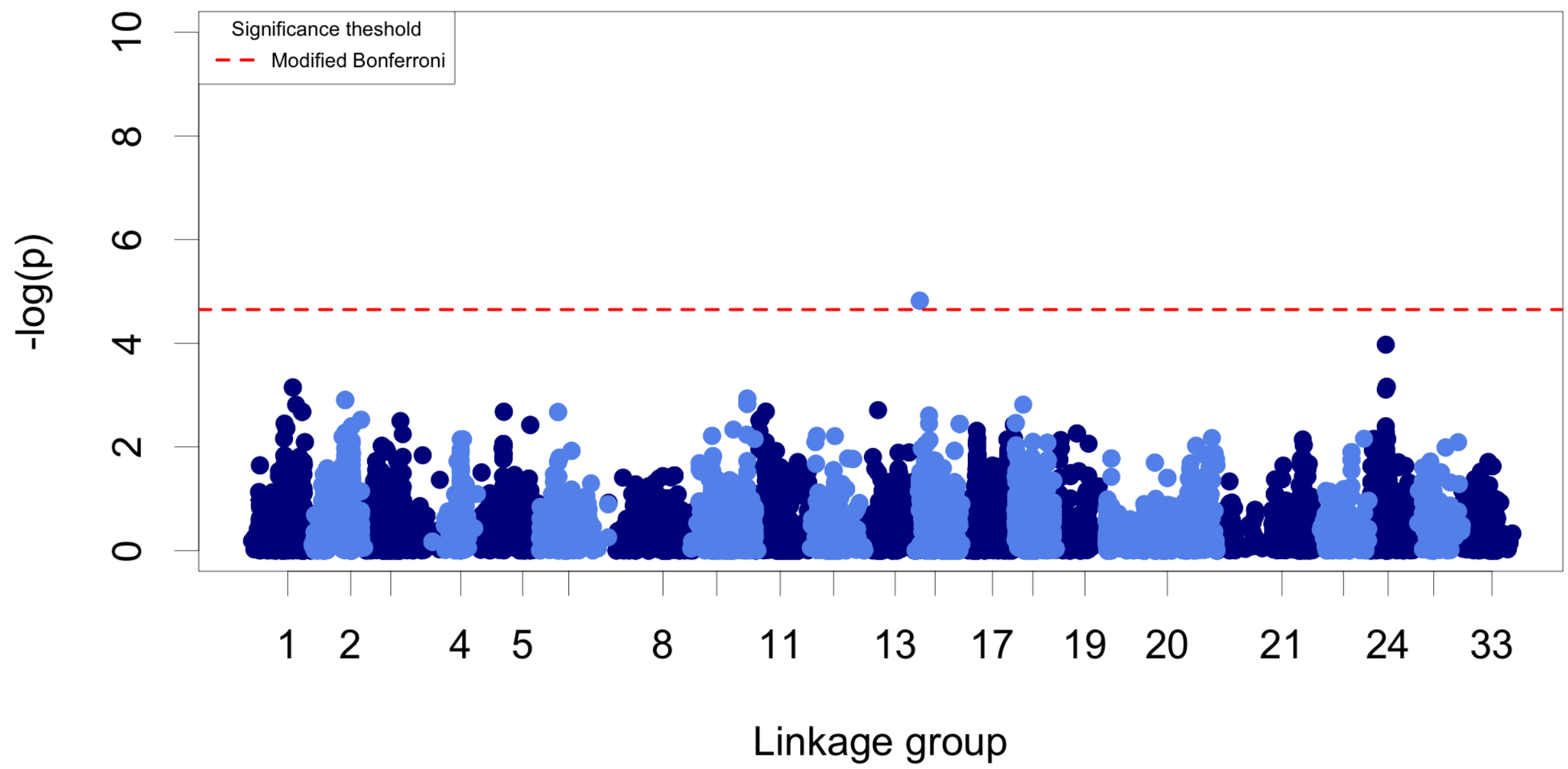

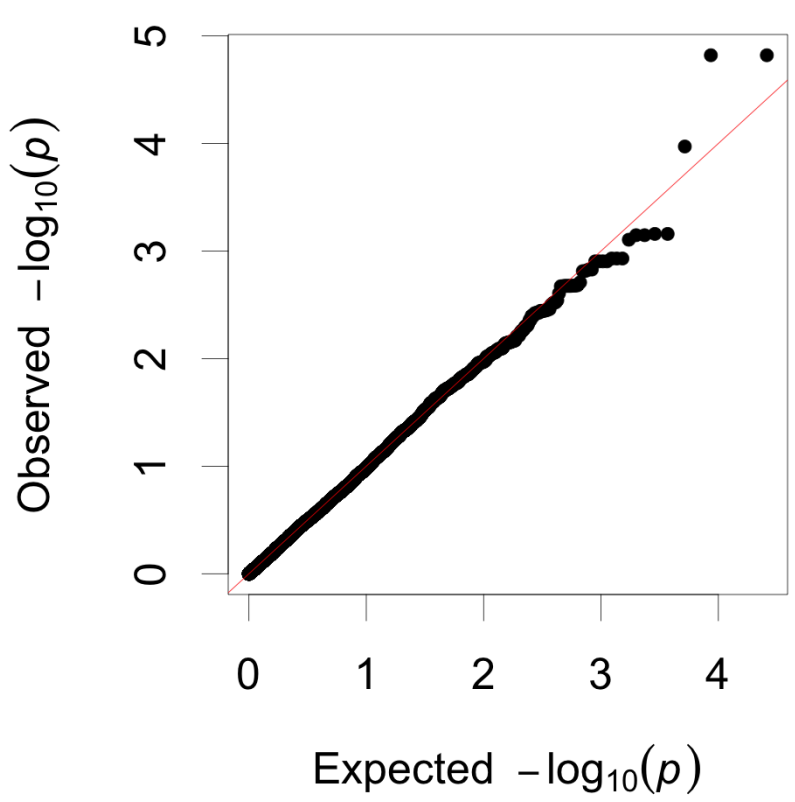

Supplement: Supplementary file 2 [file TPG2-13-e20007-s002.pdf]
