# Supplementary material for: Mapping crown rust resistance at multiple time points in elite oat germplasm
Source: Plant Genome. 2020 Apr 2;13(1):e20007. doi: 10.1002/tpg2.20007 (PMC12806915; doi:10.1002/tpg2.20007)
Supplement: Supplementary file 3 [file TPG2-13-e20007-s008.pdf]

Mrg01

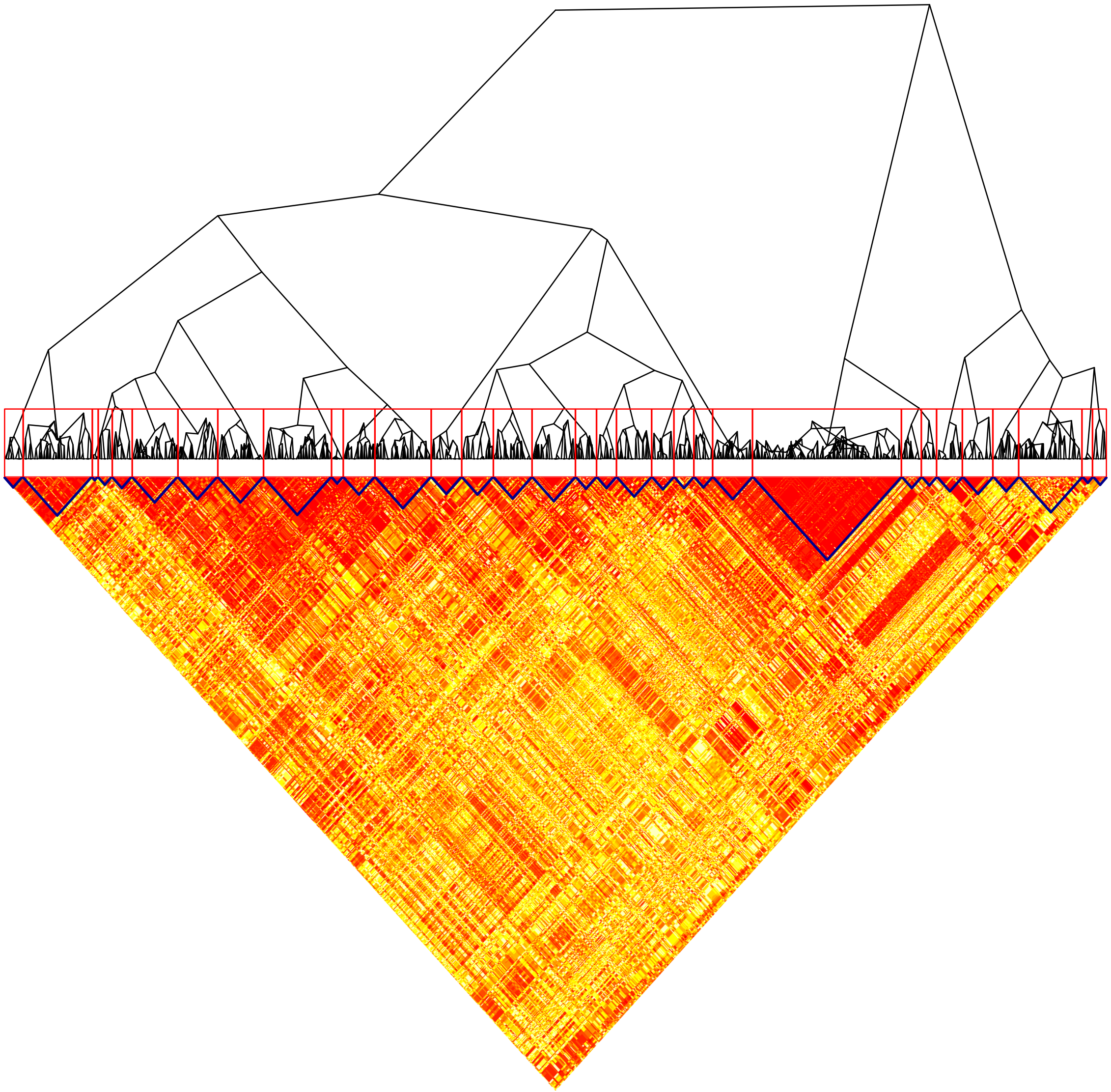

Figure 1:

Mrg02

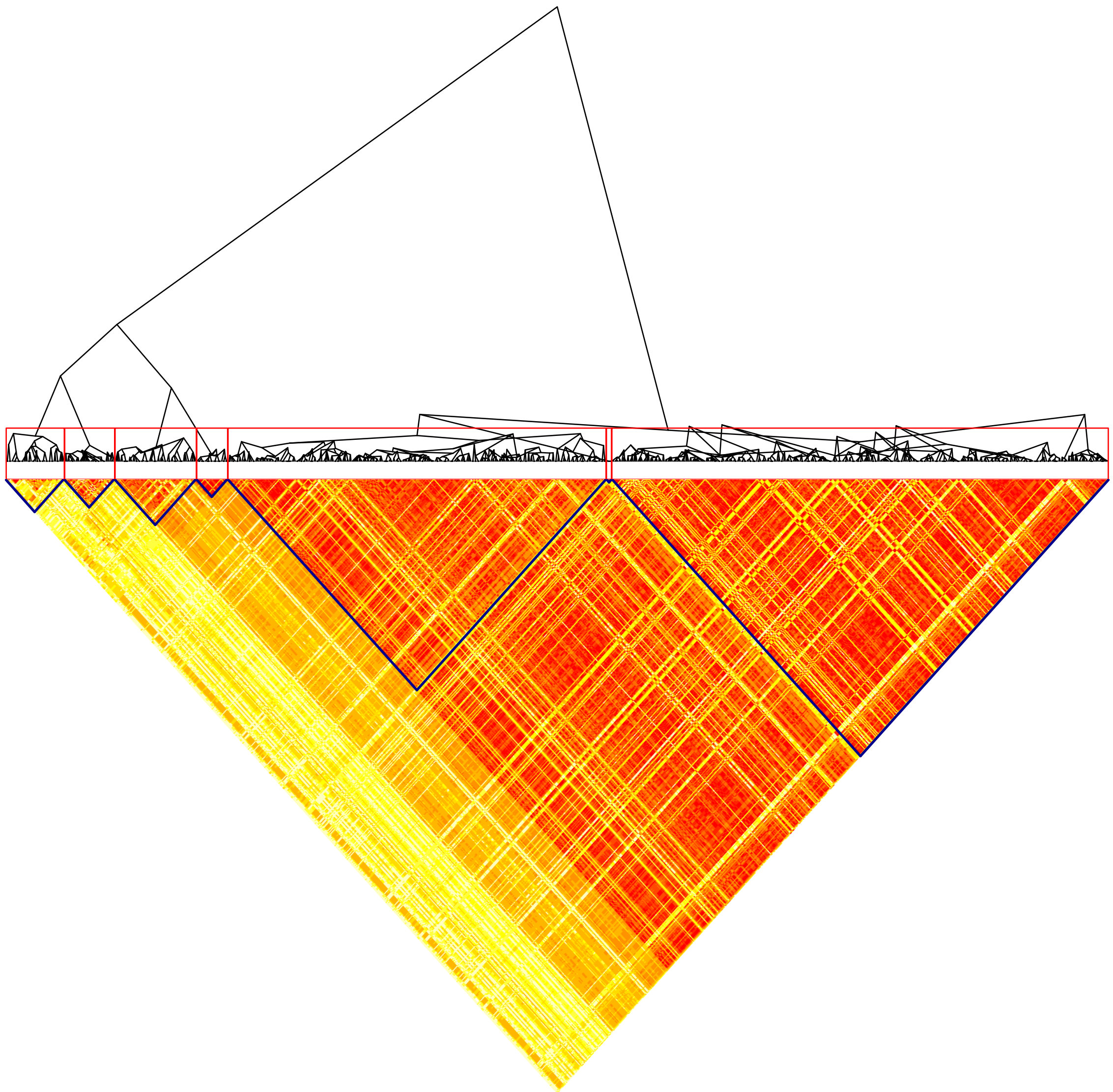

Figure 2:

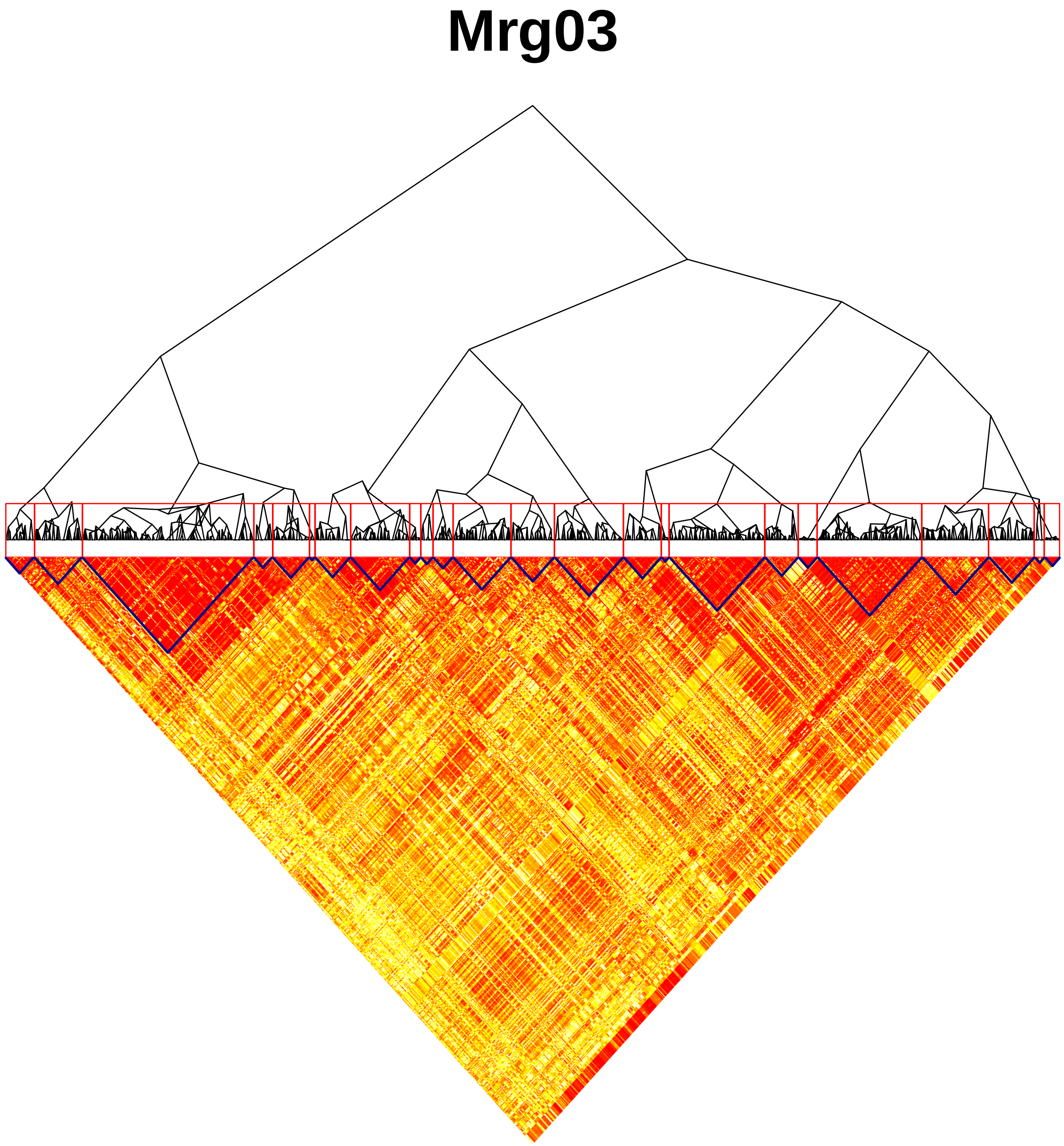

Figure 3:

Mrg04

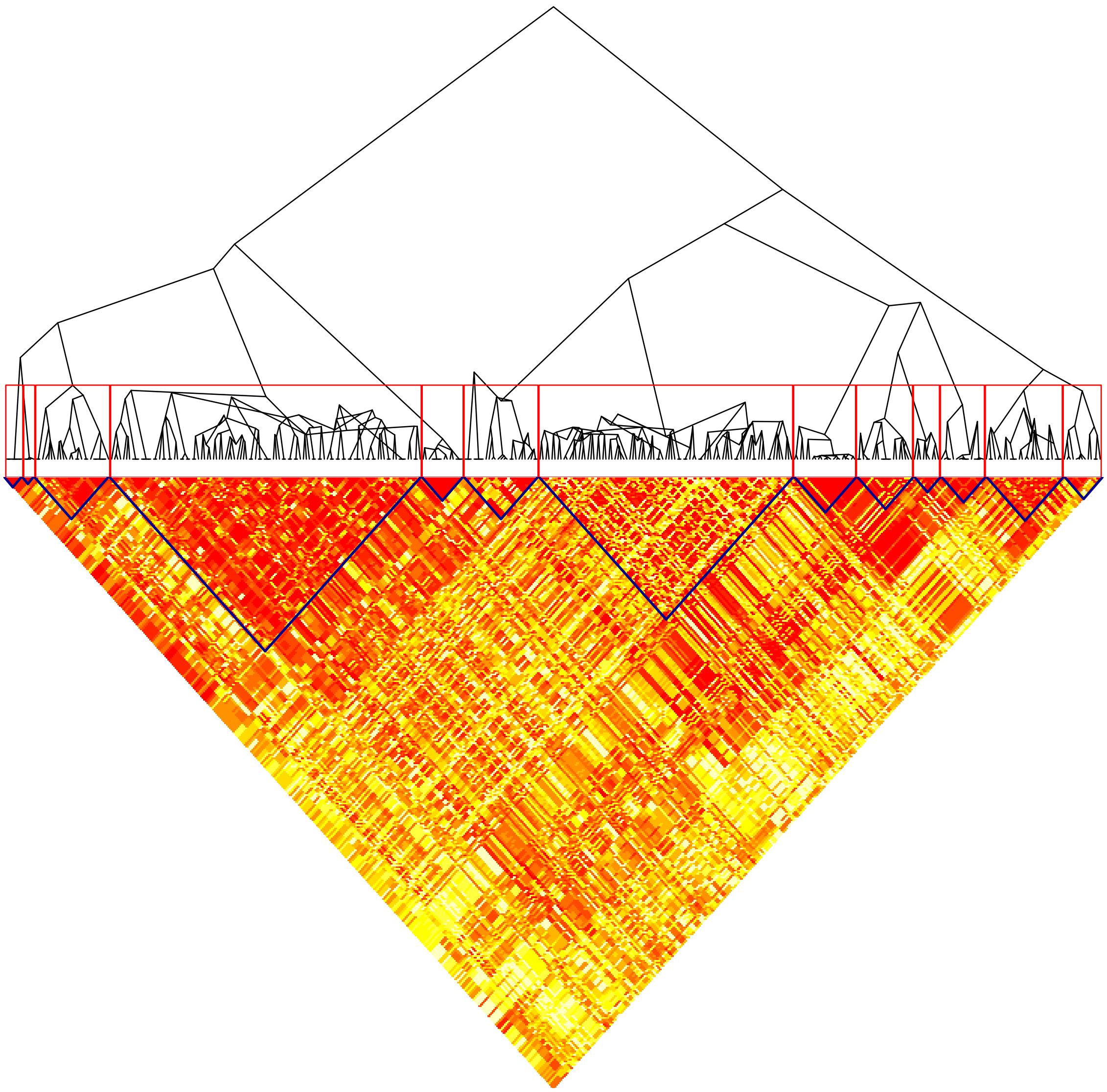

Figure 4:

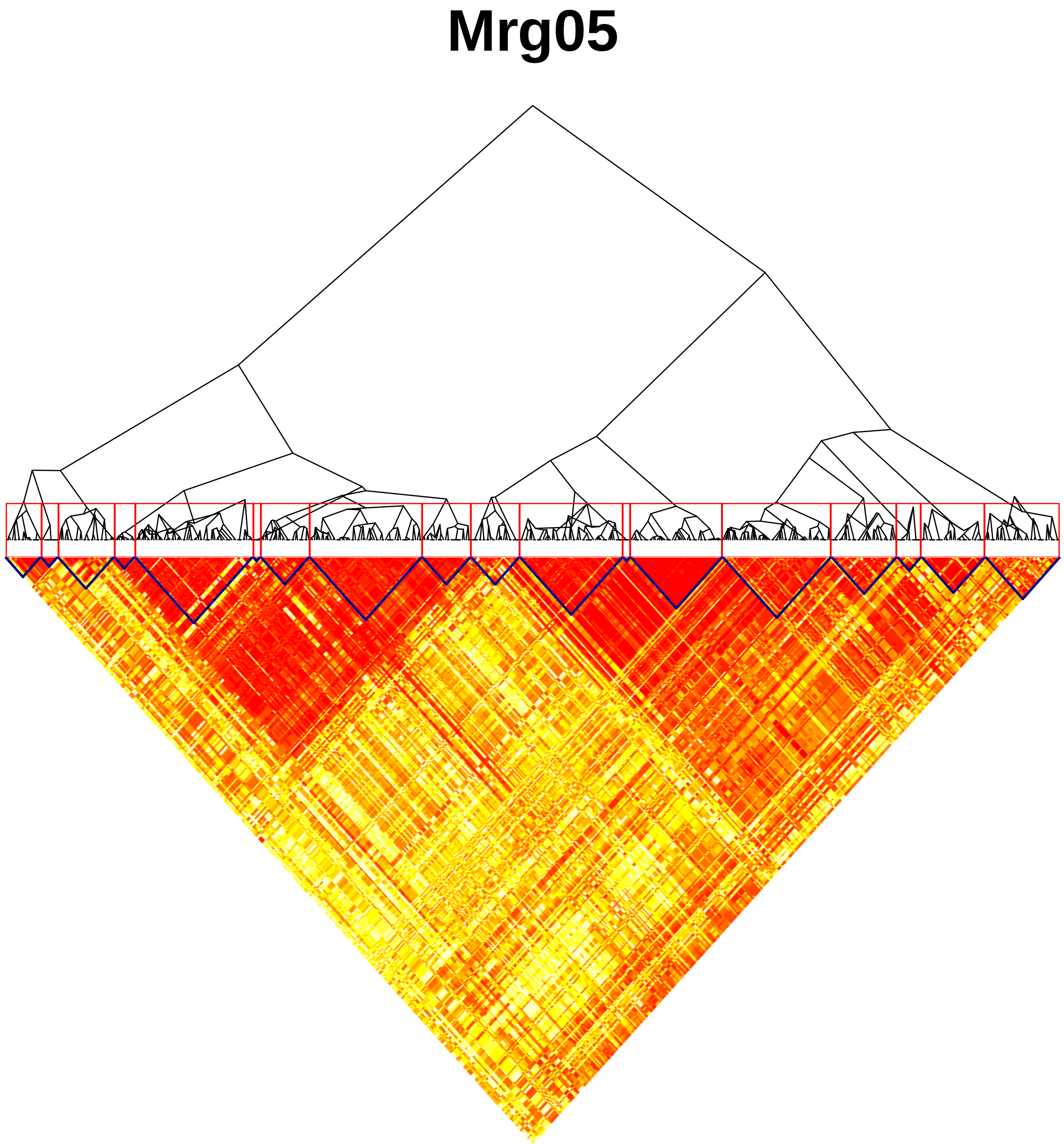

Figure 5:

Mrg06

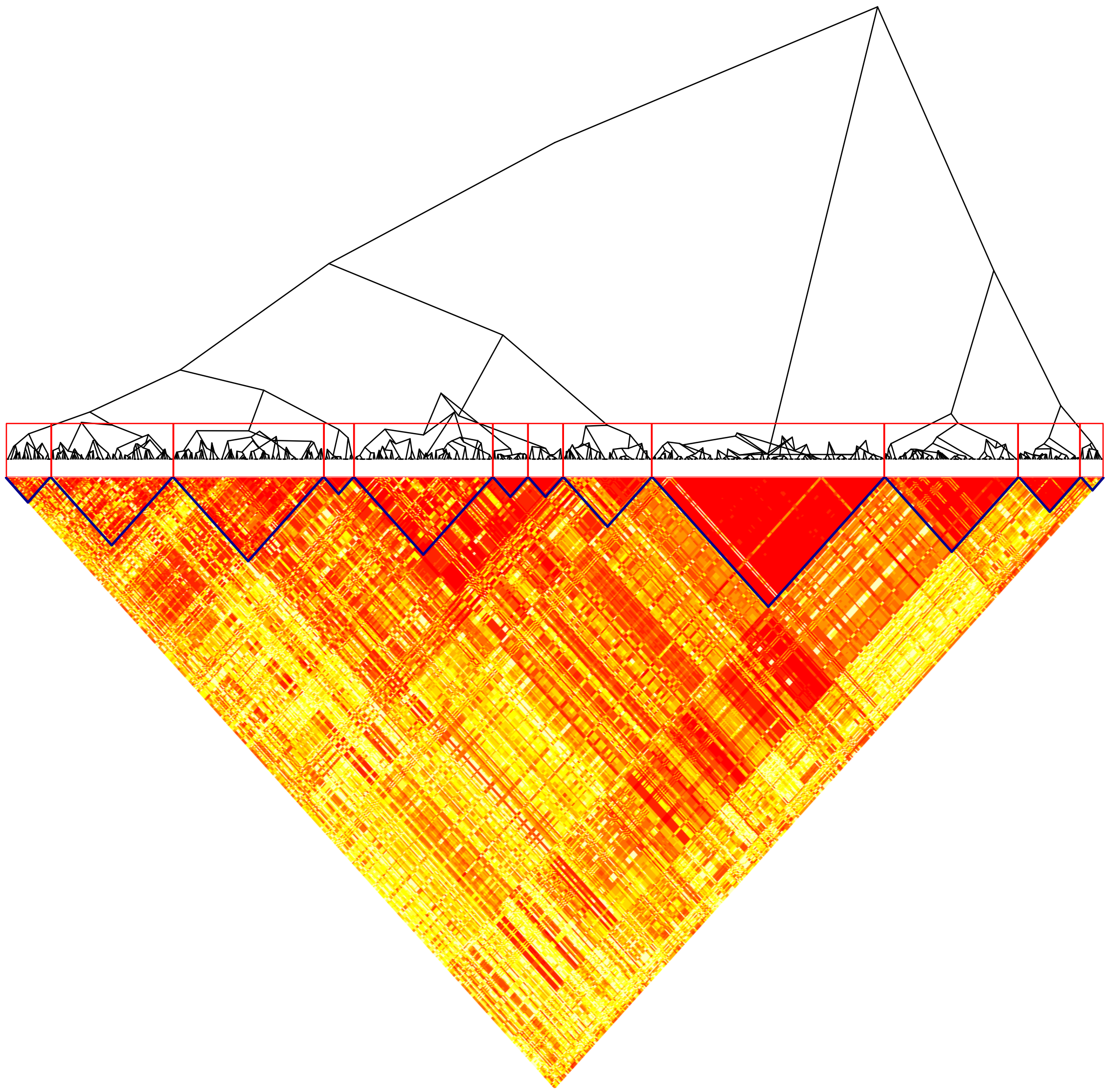

Figure 6:

Mrg08

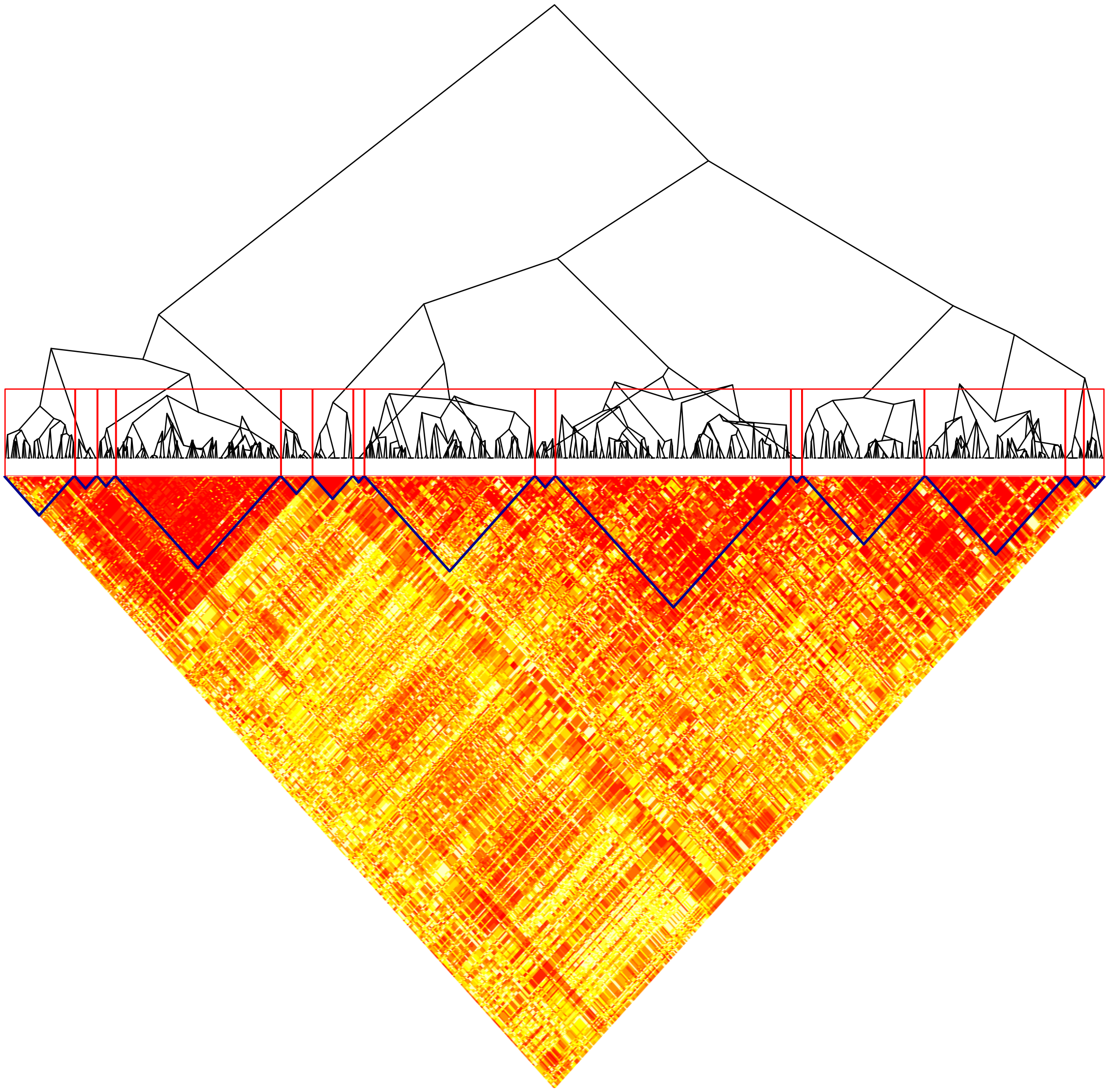

Figure 7:

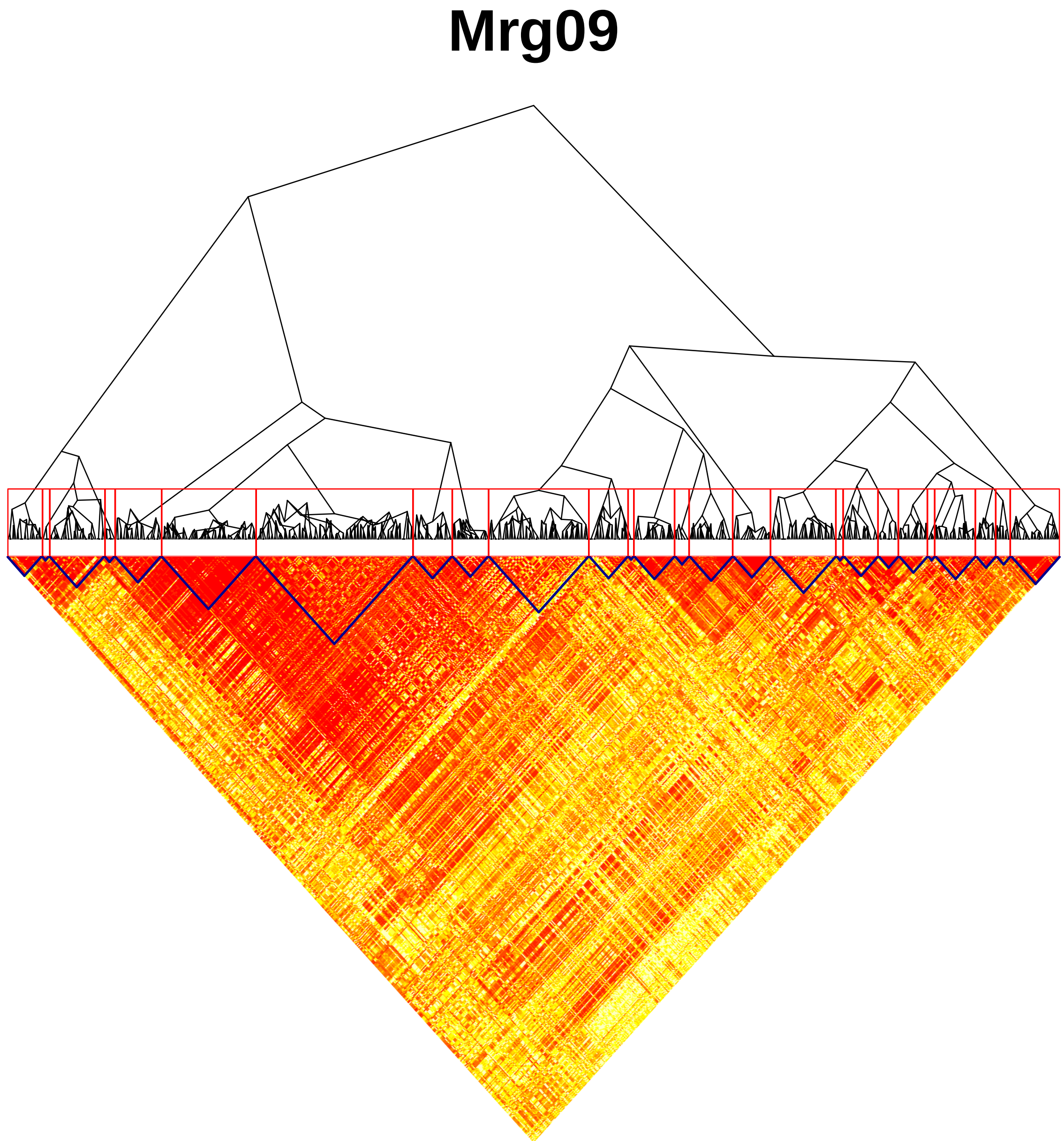

Figure 8:

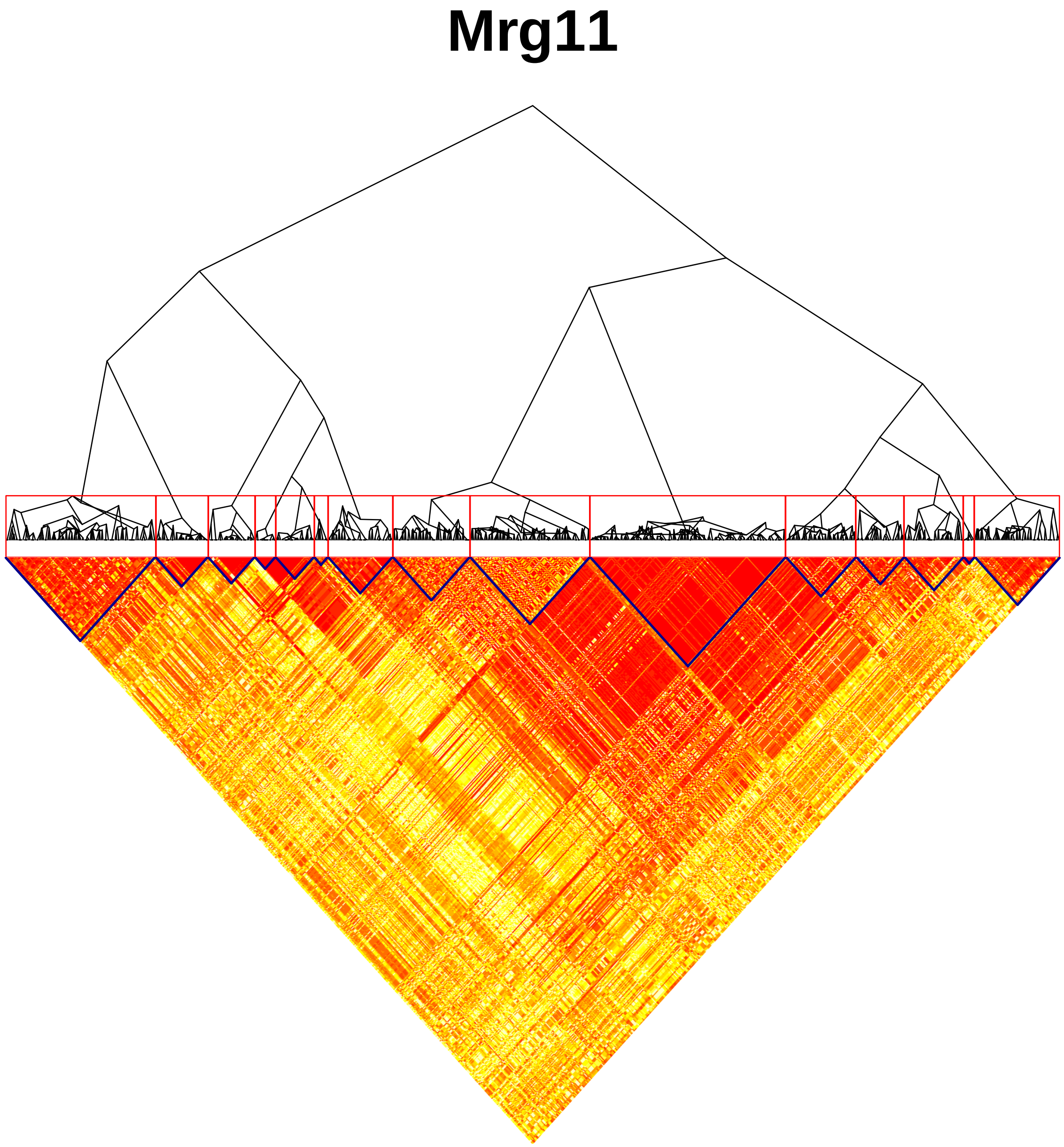

Figure 9:

Mrg12

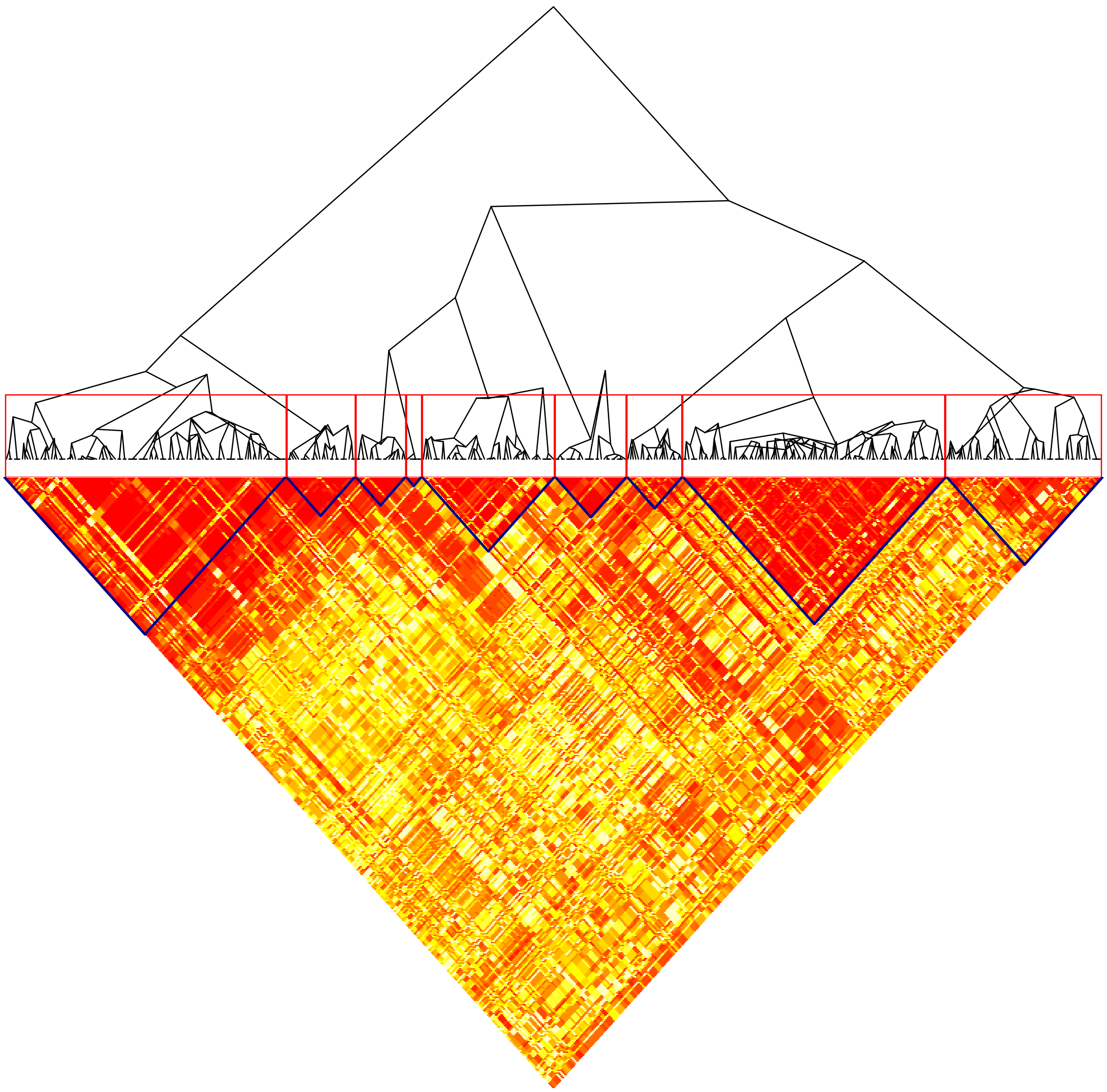

Figure 10:

Mrg13

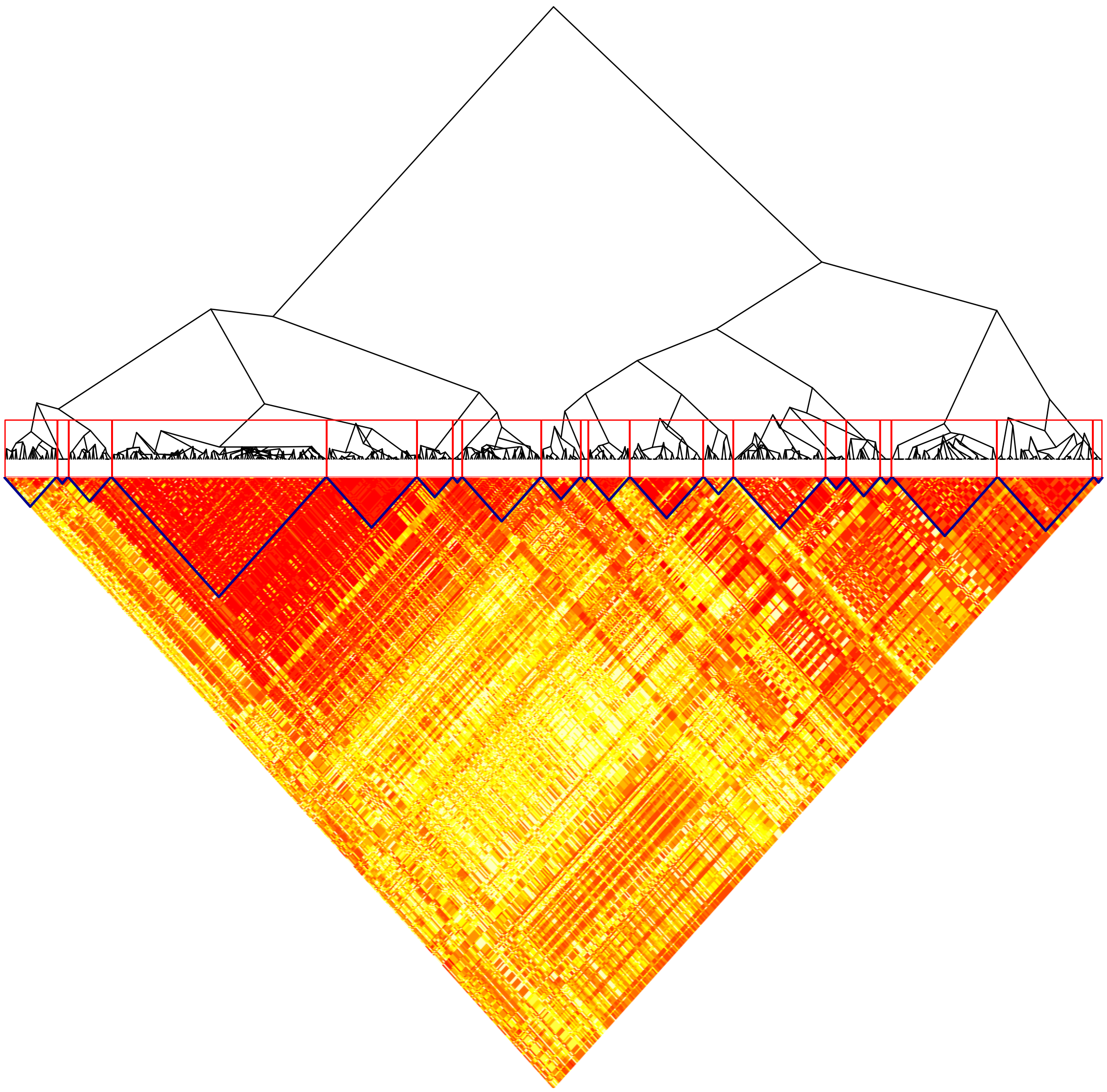

Figure 11:

Mrg15

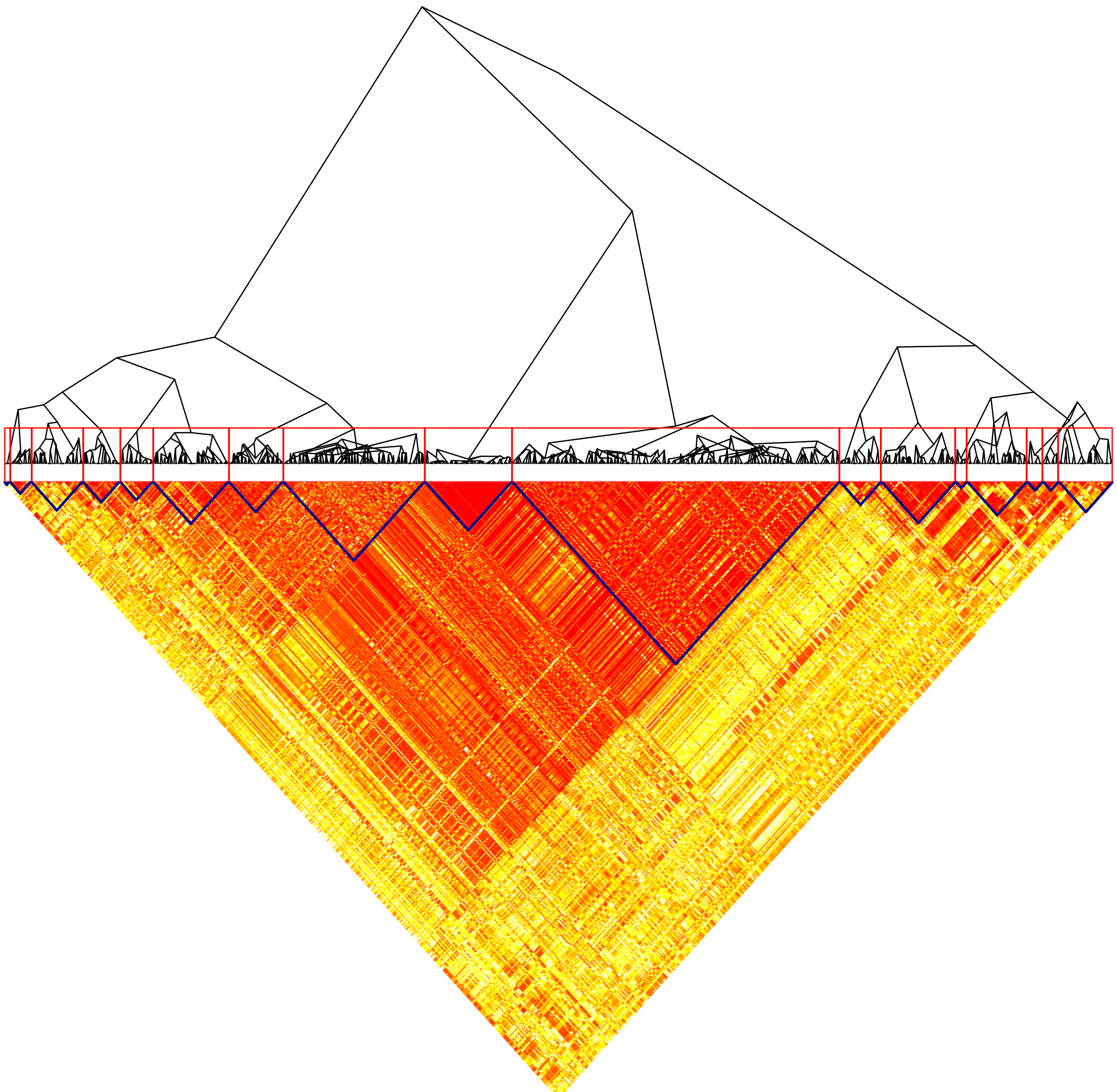

Figure 12:

Mrg17

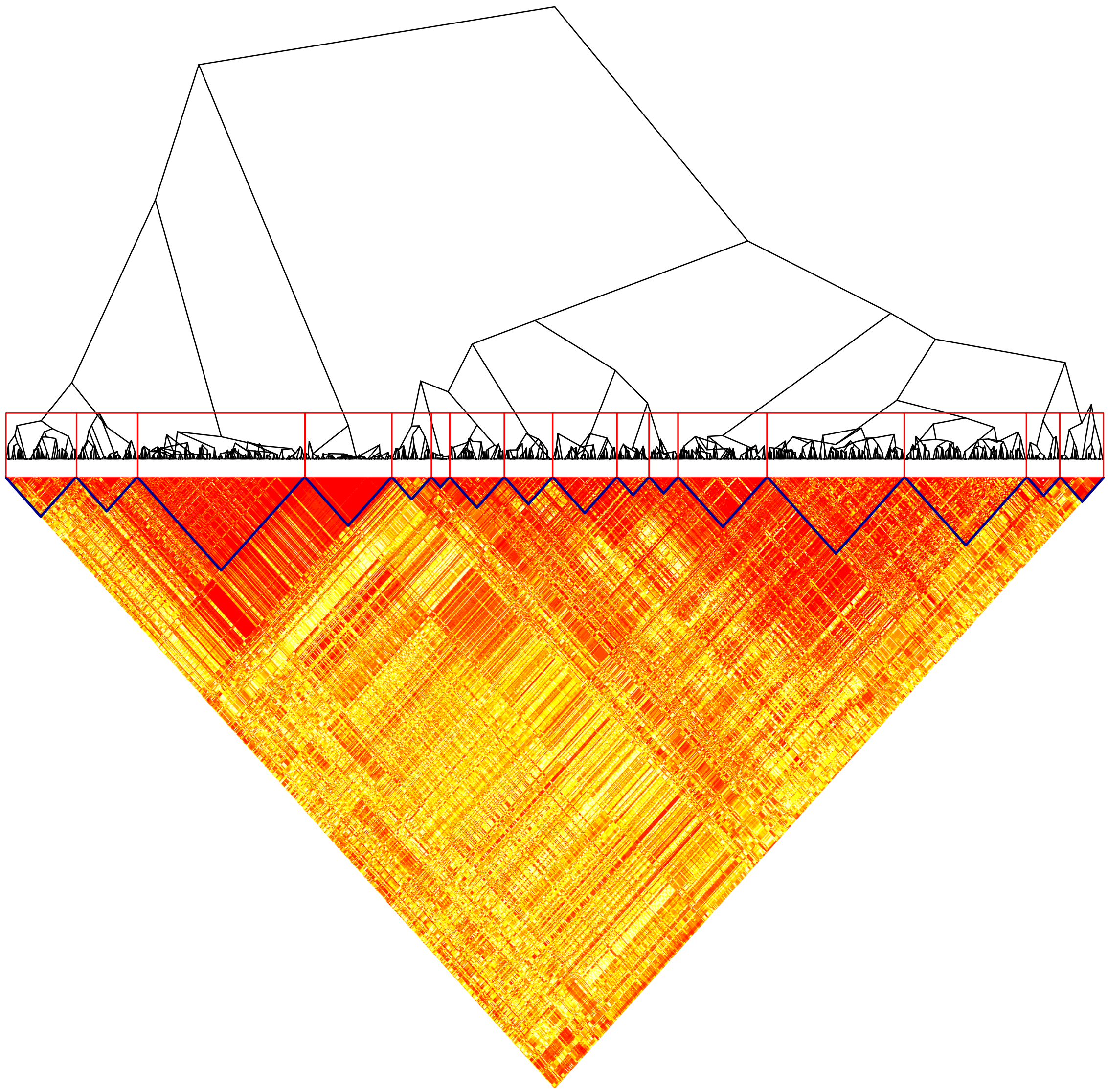

Figure 13:

Mrg18

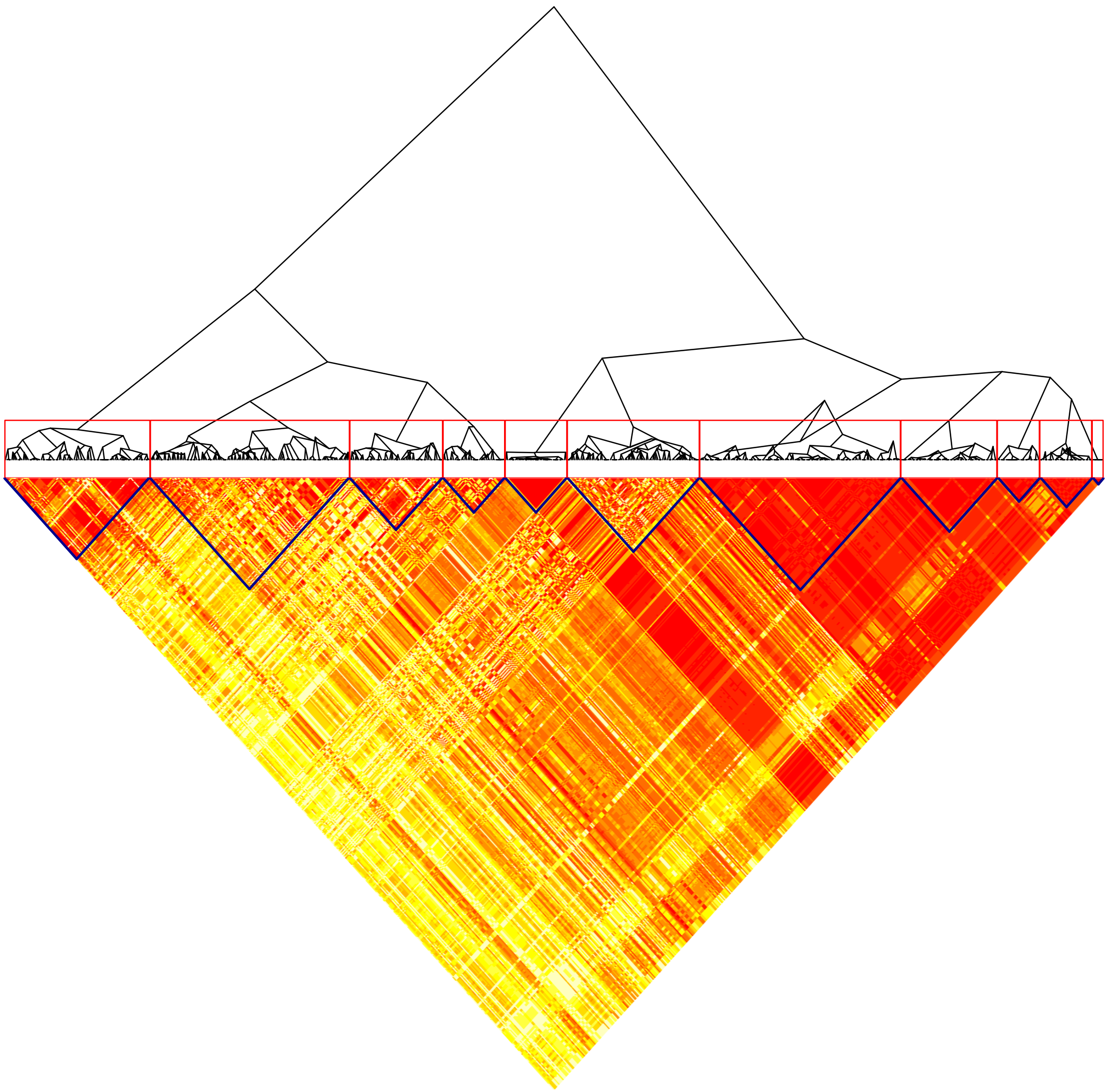

Figure 14:

Mrg19

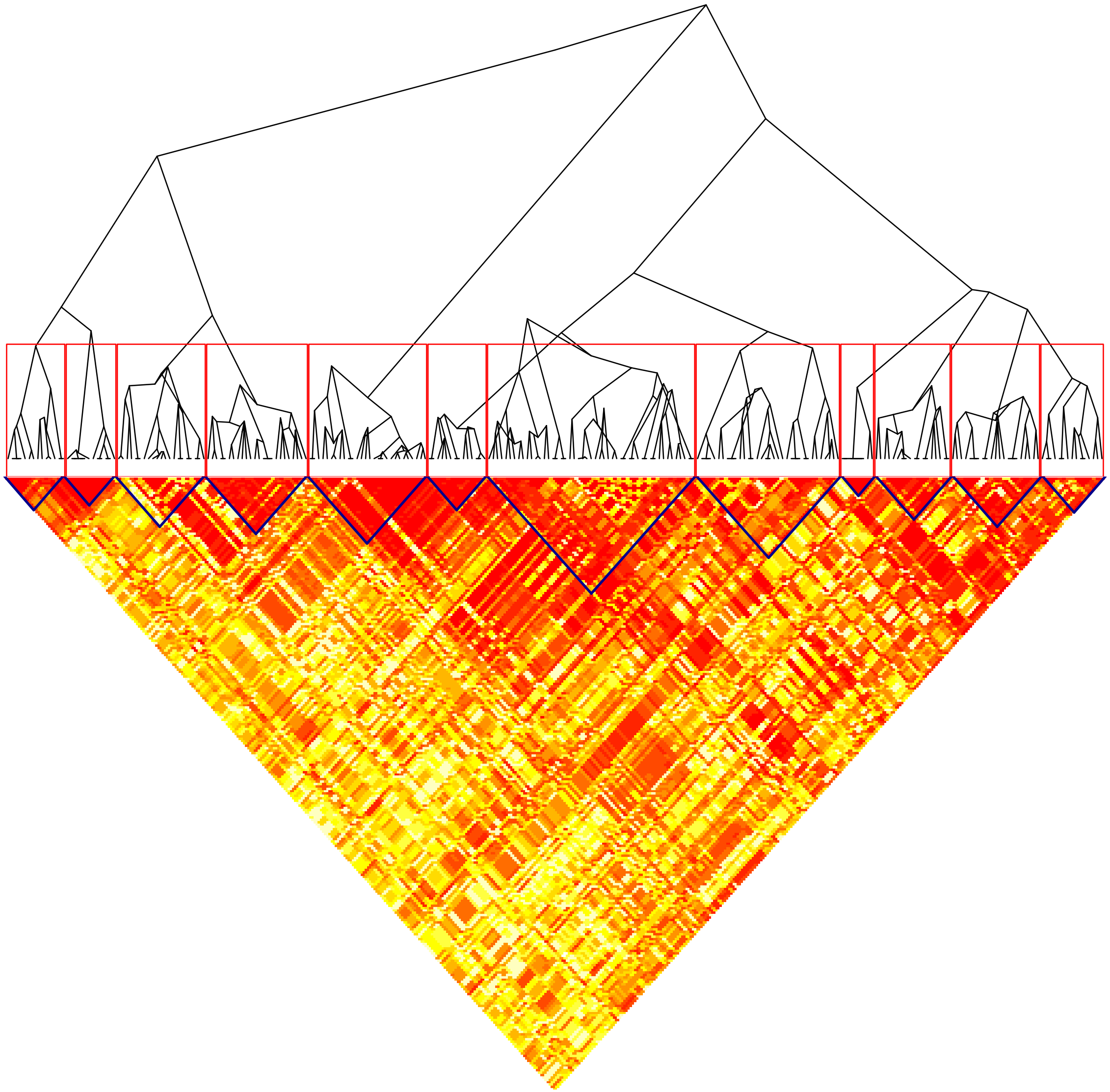

Figure 15:

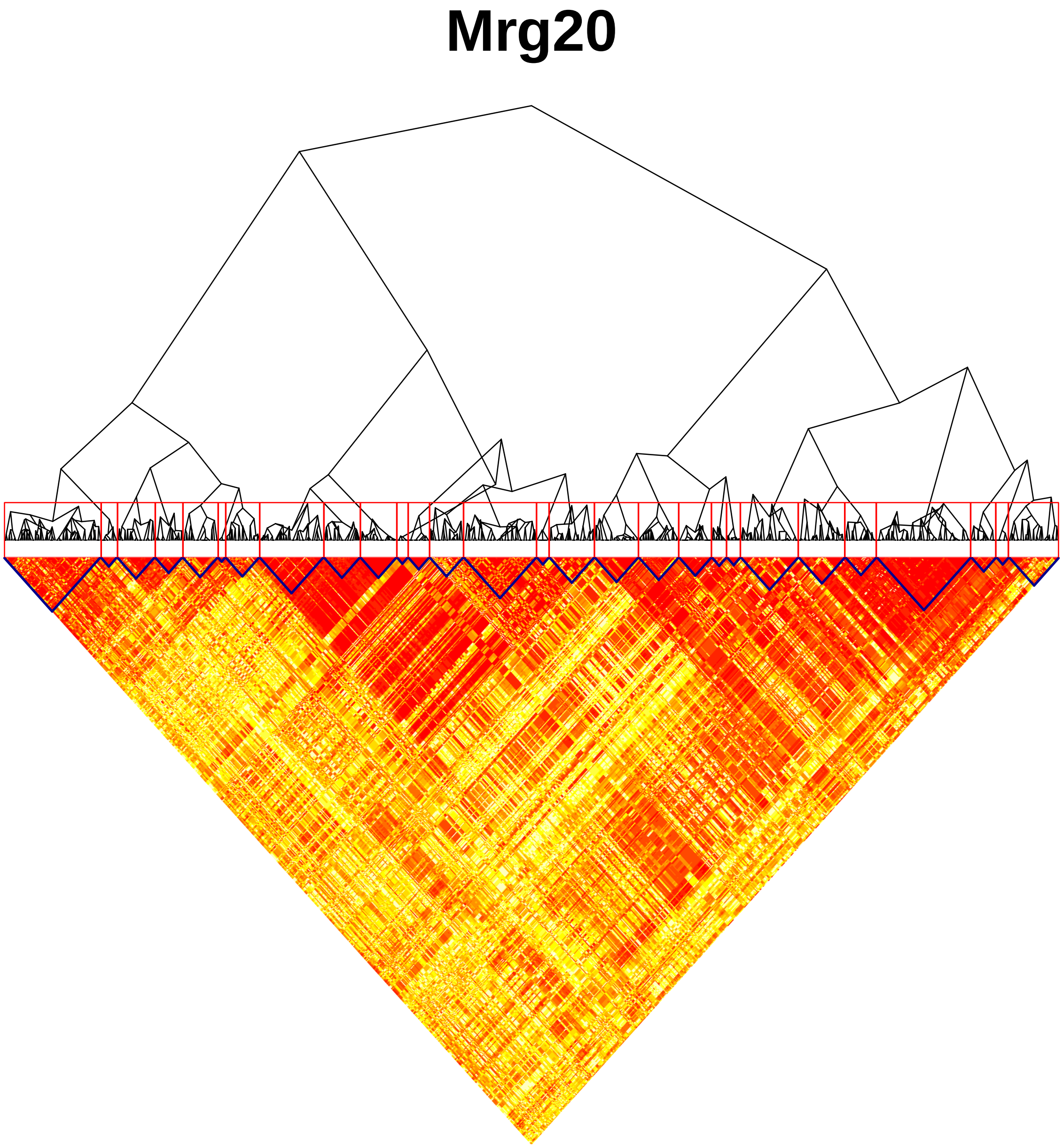

Figure 16:

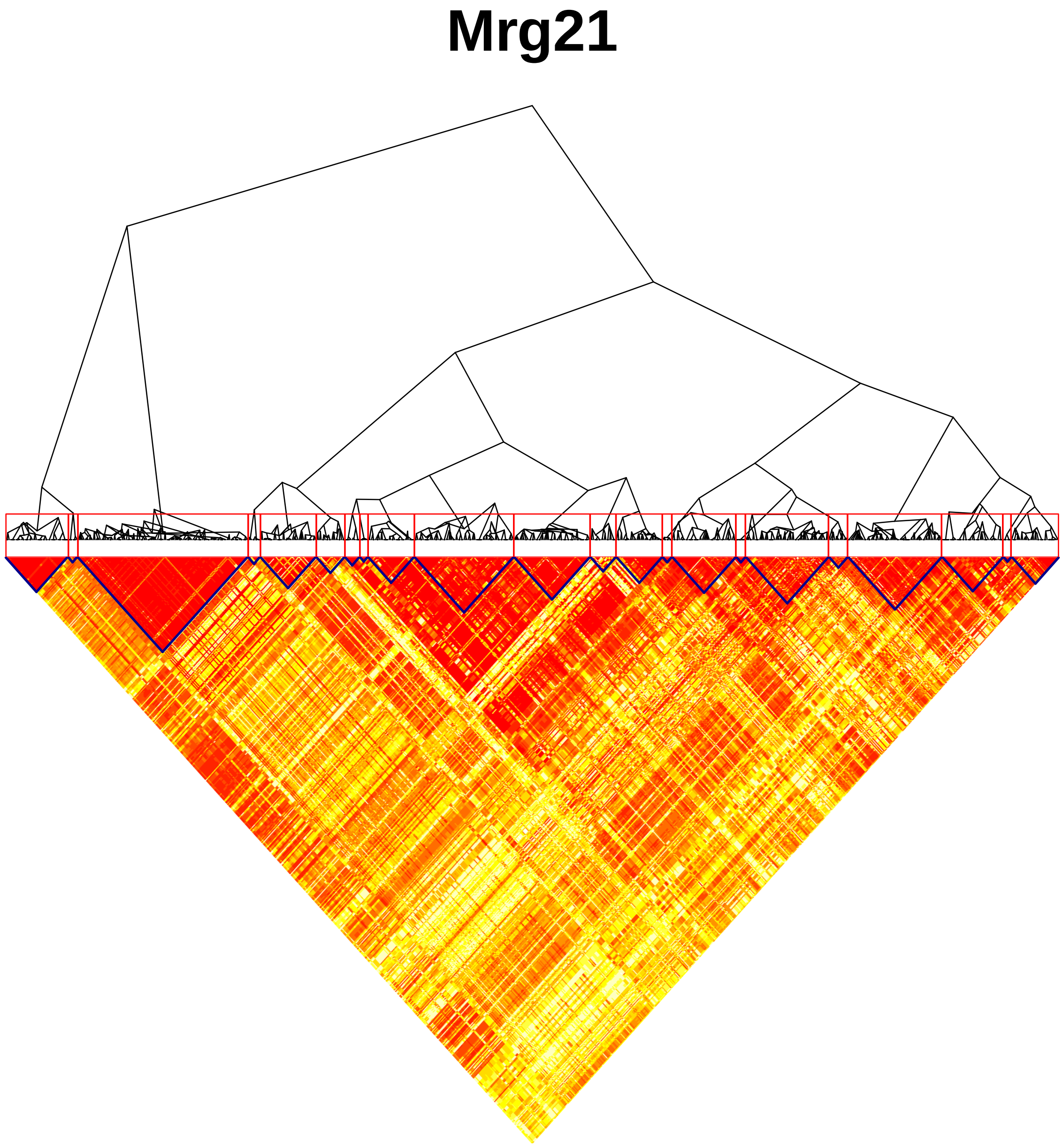

Figure 17:

Mrg23

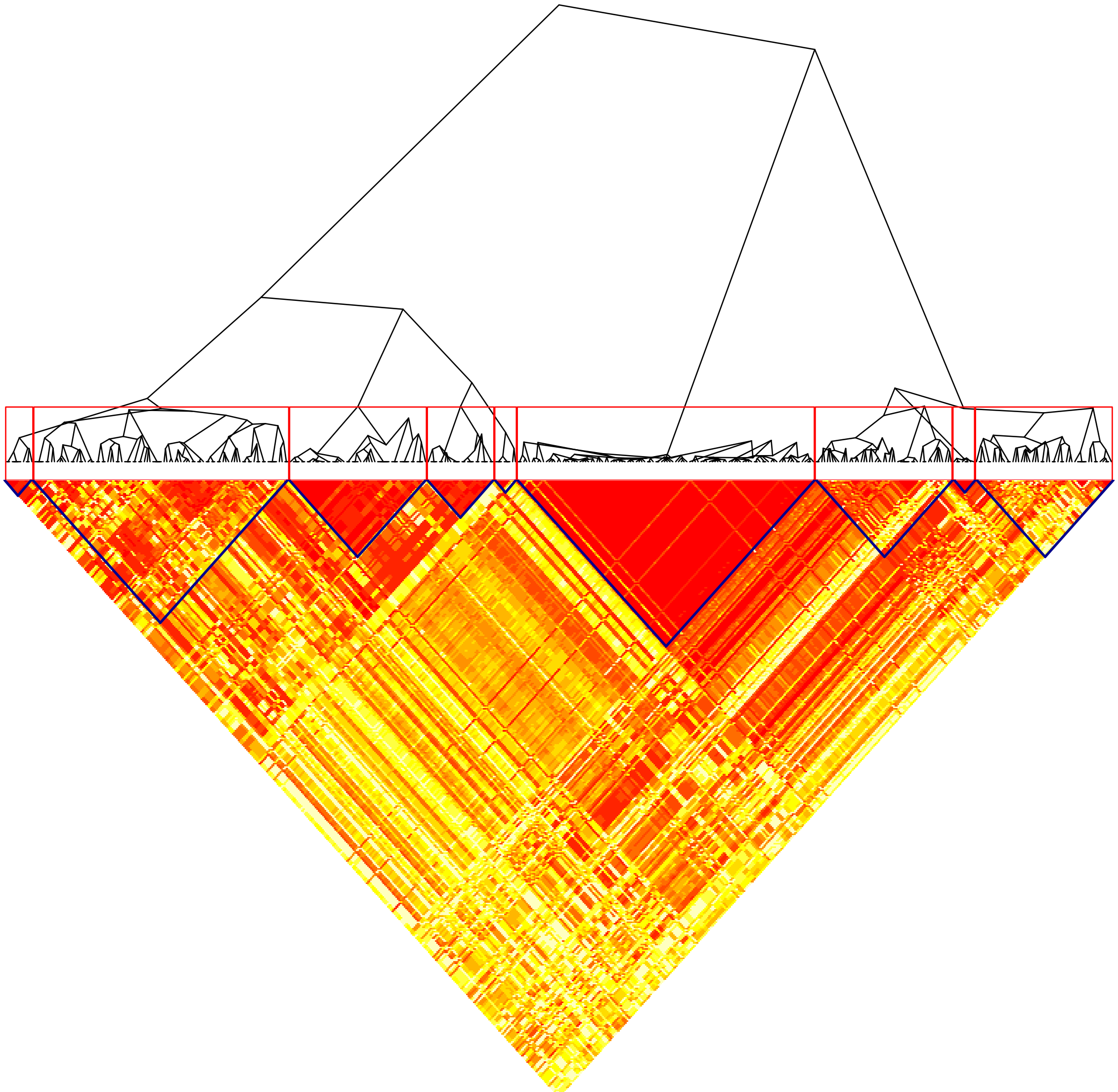

Figure 18:

Mrg24

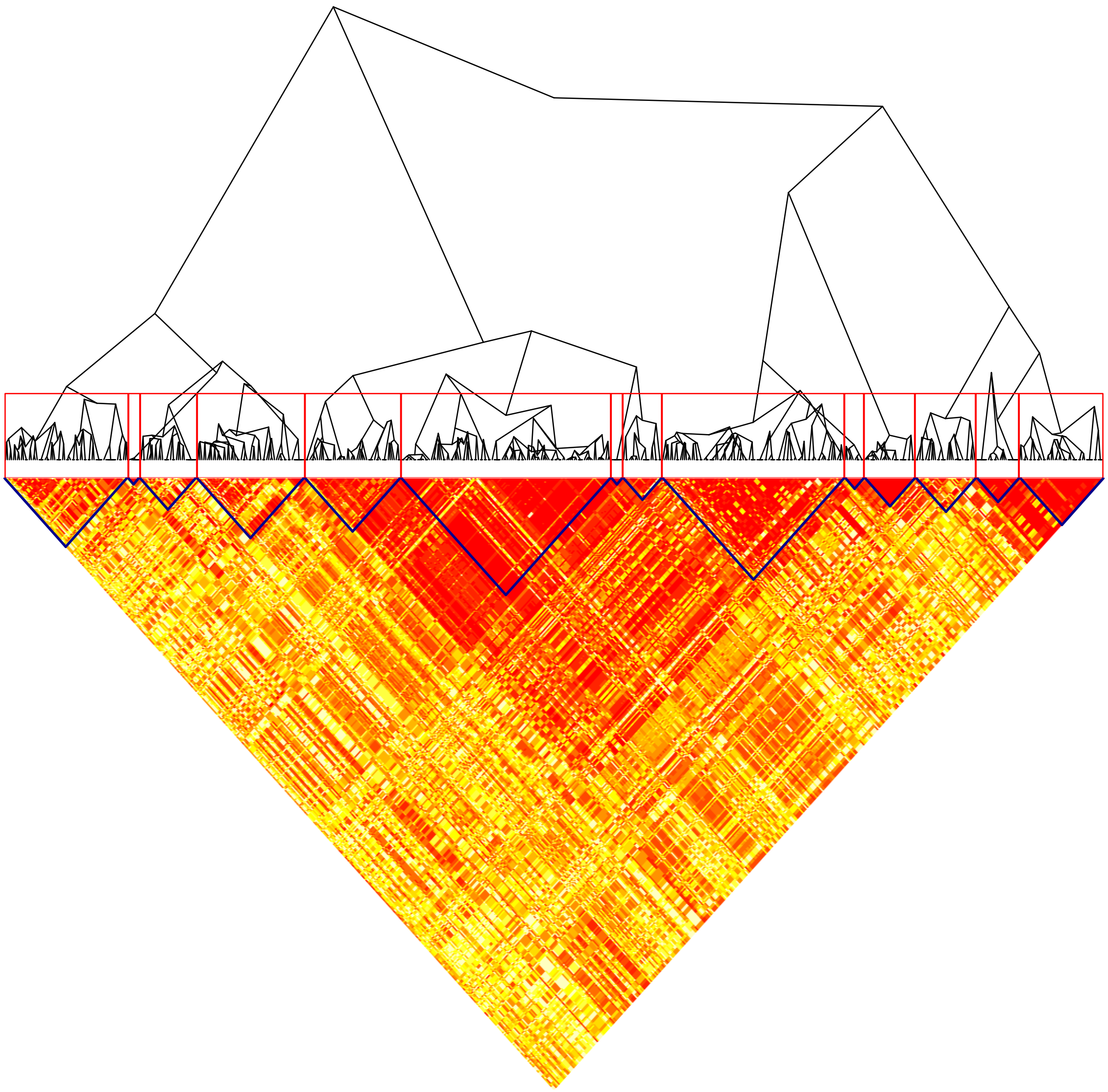

Figure 19:

Mrg28

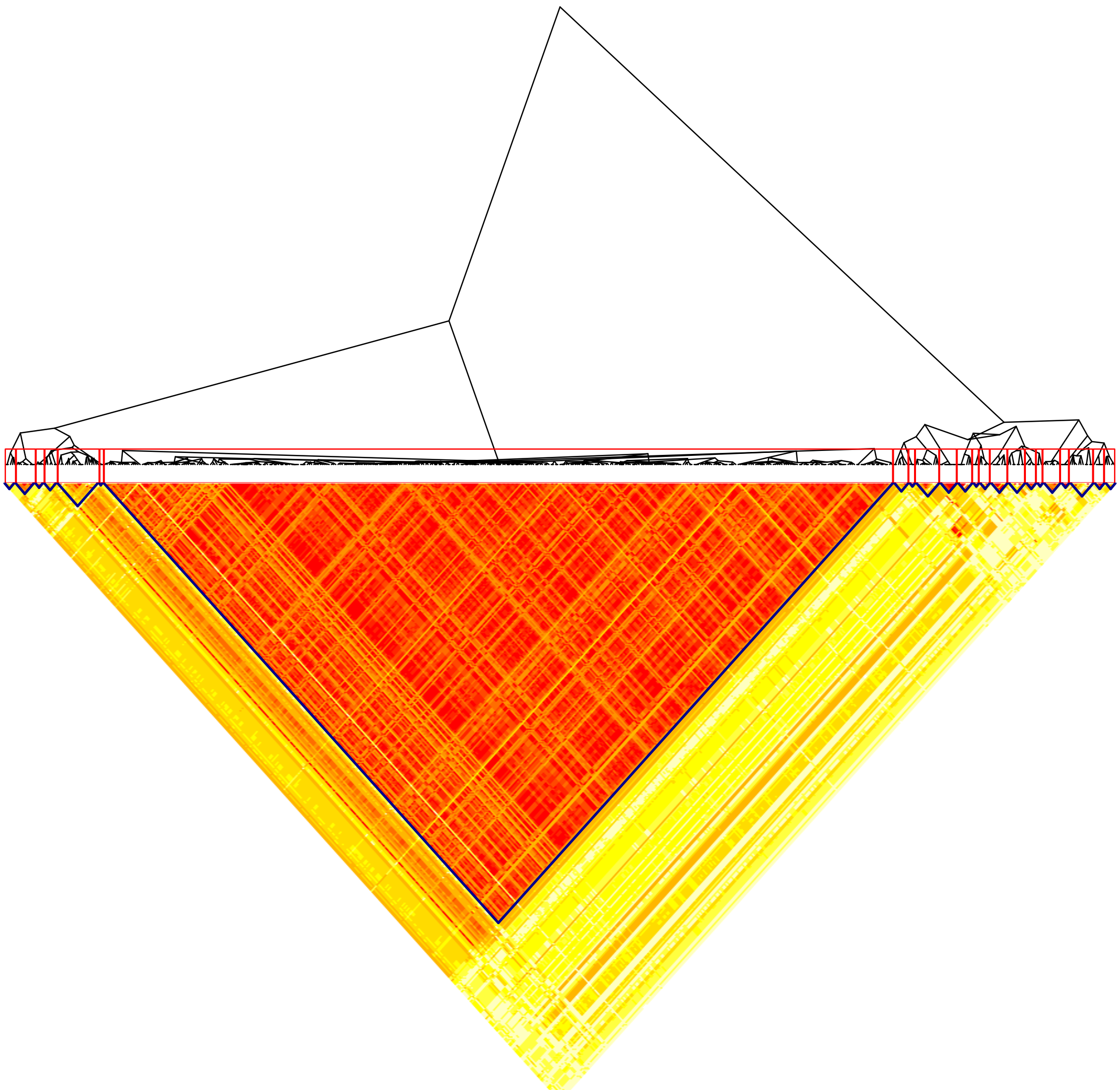

Figure 20:

Mrg33

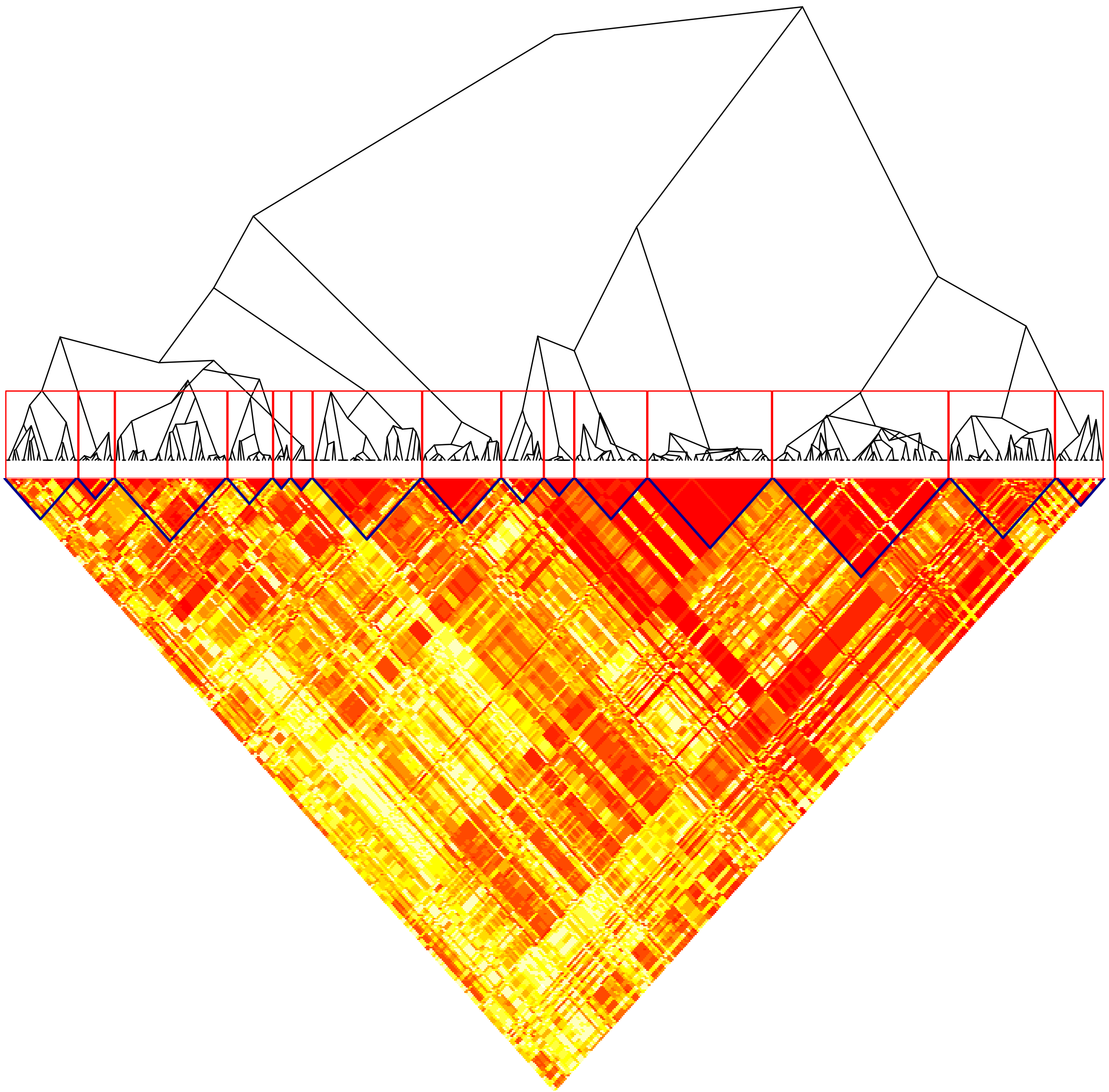

Figure 21:
