# Supplementary material for: Mapping crown rust resistance at multiple time points in elite oat germplasm
Source: Plant Genome. 2020 Apr 2;13(1):e20007. doi: 10.1002/tpg2.20007 (PMC12806915; doi:10.1002/tpg2.20007)
Supplement: Supplementary file 4 [file TPG2-13-e20007-s006.docx]

**Supplemental material for article “Mapping Crown Rust Resistance at Multiple Time Points in Elite Oat Germplasm” by Ian G. McNish, Cristiano M. Zimmer, Alexander Q. Susko, D. Jo Heuschele, Tyler Tiede, Austin J. Case, and Kevin P. Smith**

**doi:10.3835/plantgenome2019.08.0080**

Supplemental Figure S1: Founder population scree plot.

Supplemental Figure S2: plot of the k-means gap statistic of University of Minnesota Oat Founder population principal component analysis data from k=1 to k=10. Error bars around each point represent one standard error. The number of clusters in this data was determined using the ‘1-standard error rule’ (Tibshirani *et al*., 2001) which identified k=3 clusters (dashed vertical line).

(PDF file) Supplemental Figure S3: Heatmaps of pairwise r^2^ and linkage block cluster analysis of each linkage group from Bekele *et al.* (2018).

(PDF file) Supplemental Figure S4: Correlation plots for each trial. Scatter plots for each pair of variables are below the axis. Histograms are on the axis. Pearson correlations (r) are above the axis. Stars indicate level of statistical significance (H_0_: ⍴ = 0): * significant at the 0.05 level, ** significant at the 0.01 level, and *** significant at the 0.001 level.

(PDF file) Supplemental Figure S5: Manhattan and quantile-quantile (Q-Q) plots for all genome-wide association models. Linkage group and genetic positions are from Bekele *et al.* (2018).

(Excel file) Supplemental Table S1: Pairwise r^2^ matrices for each Bekele *et al.* (2018) linkage group. R^2^ values are above the diagonal. Zeros placed below the diagonal have no meaning.

(Excel file) Supplemental Table S2: Linkage block assignments for each marker determined by adjacency-constrained hierarchical agglomerative clustering. Linkage groups are from Bekele *et al.* (2018) and positions are in centiMorgans (cM).

(Excel file) Supplemental Table S3: Full genome-wide association mapping results for each genome-wide association model. Marker sequences are from the T3/Oat database. Linkage group and genetic positions are from Bekele *et al.* (2018). Values reported for each trait are -log_10_(p) values.
